# A revision of the Chinese Gasteruptiidae (Hymenoptera, Evanioidea)

**DOI:** 10.3897/zookeys.237.3956

**Published:** 2012-11-02

**Authors:** Ke-xin Zhao, Cornelis van Achterberg, Zai-fu Xu

**Affiliations:** 1 Department of Entomology, College of Natural Resources and Environment, South China Agricultural University, Guangzhou 510640, P. R. China; 2 Department of Terrestrial Zoology, Naturalis Biodiversity Center, Postbus 9517, 2300 RA Leiden, the Netherlands

**Keywords:** Revision, Gasteruptiidae, *Gasteruption*, keys, new species, new synonyms, lectotypes, China

## Abstract

The Chinese fauna of the family Gasteruptiidae is revised, keyed and fully illustrated for the first time. Only one genus of this family, *Gasteruption* Latreille, 1796, is recorded from China. In total 28 valid species of the genus *Gasteruption* are recognized. Six species are new to science (*Gasteruption angulatum* **sp. n.**, *Gasteruption assectoides* **sp. n.**, *Gasteruption coloratum* **sp. n.**, *Gasteruption latitibia* **sp. n.**, *Gasteruption sinepunctatum* **sp. n.** and *Gasteruption strigosum* **sp. n.)** and eight species are reported new for China (*Gasteruption bimaculatum* Pasteels, 1958, *Gasteruption birmanense* Pasteels, 1958, *Gasteruption dimidiatum* Semenov, 1892, *Gasteruption formilis* Alekseev, 1995, *Gasteruption subhamatum* Pasteels, 1958, *Gasteruption tonkinense* Pasteels, 1958, *Gasteruption tournieri* Schletterer, 1885, *Gasteruption transversiceps* Pasteels, 1958). Three new synonyms are proposed: *Gasteruption curiosum* Pasteels, 1958, of *Gasteruption amoyense* Pasteels, 1958; *Gasteruption sinense* var. *minus* Kieffer, 1924, with *Gasteruption japonicum* Cameron, 1888,and *Gasteruption sinense* Kieffer, 1924, of *Gasteruption sinarum* Kieffer, 1911. Lectotypes are designated for *Gasteruption corniculigerum* Enderlein, 1913, *Gasteruption sinense* Kieffer, 1924, and *Gasteruption transversiceps* Pasteels, 1958. *Gasteruption bihamatum* Kieffer, 1911, previously reported from South China, is a South American species.

## Introduction

Gasteruptiidae is a family in Evanioidea of Hymenoptera with a worldwide distribution, including two extant subfamilies, Hyptiogastrinae and Gasteruptiinae, and one extinct subfamily, Kutujellitinae, with about 500 nominal species (Crosskey 1962; Rasnitsyn 1991; Macedo 2011). Most species of this family belong to Gasteruptiinae, which consists of more than 400 species worldwide. The subfamily Hyptiogastrinae occurs almost entirely in the Australian region (but with two Neotropical species) (Jennings and Austin 2002), with about 89 described species (Jennings 2010). Biologically, species of Gasteruptiidae are predator-inquilines (sometimes referred to as kleptoparasites) on larvae of bees nesting in soil, stems and in tunnels in wood, including Apinae, Colletinae, Halictinae and Megachilinae. They first feed on the bee larva and proceed to consume the stored pollen; if this is not sufficient, another cell’s contents may be used (Malyshev 1966). There is only indirect evidence for wasps (Crabronidae, Sphecidae and Vespidae-Eumeninae) being hosts of Gasteruptiidae (Crosskey 1951; Gauld and Hanson 1995; Jennings and Austin 1997a, 1997b, 2004). Partly, it may concern re-used nests by bees; without future investigations it is not clear if gasteruptiid wasps oviposit in the nests of wasps. The mature larva makes a cocoon in the host's nest to hibernate until the next summer, when the larvae pupate (He 2004; Jennings and Austin 2004). Adult Gasteruptiidae are usually collected around dead tree trunks, clay banks, other nesting sites of their hosts, and on flowers (Gupta 1979). They are easily recognized from other wasps by the elongate neck-like propleuron, very high insertion of the metasoma on the propodeum, clavate hind tibia, 14-segmented antenna in females (13-segmented in males) and the relatively long eye, extending almost to the mandible (Crosskey 1951; Jennings and Austin 2004; Smith 2006).

In a recent phylogenetic analysis of South American Gasteruptiinae (Macedo 2009), four genera of the subfamily were proposed: *Gasteruption* Latreille, 1796, *Plutofoenus* Kieffer, 1911, *Spinolafoenus* Macedo, 2009 and *Trilobitofoenus* Macedo, 2009. Among them, *Gasteruption* is predominant and the only worldwide genus in this subfamily, while the other three genera are restricted to the Neotropical region and include seven species in total (Macedo 2009).

In the subfamily Hyptiogastrinae two genera are known: *Hyptiogaster* Kieffer, 1903, and *Pseudofoenus* Kieffer, 1902. Ten species of *Hyptiogaster* are known from Australia. About 79 species of *Pseudofoenus* are known, 77 species from the Australian region and two from South America (Jennings and Austin 2002, 2005).

The Chinese fauna of the family Gasteruptiidae has never been revised, keyed or fully illustrated. Pasteels (1958) reported ten species of *Gasteruption* from China, but he overlooked the paper by Kieffer (1924) with three additional species from the Shanghai area. To date, 16 species are known from China (Table 1), of which 14 species are valid; three are synonyms and one species is misidentified. Kieffer (1912) reported *Gasteruption bihamatum* Kieffer, 1911, from South China (Amoy= Xiamen), but it concerns a South American species; Macedo (2011) synonymized it with the widespread *Gasteruption bispinosum* Kieffer, 1904. Kieffer (1924) reported three species and a variety from China; one species (*Gasteruption sinense* Kieffer, 1924) is a new synonym of *Gasteruption sinarum* Kieffer, 1911, *Gasteruption abeillei* Kieffer, 1912, is a synonym of *Gasteruption assectator* (Linnaeus, 1758) (Madl 1989) and the variety *minus* of the first species is a new synonym of *Gasteruption japonicum* Cameron, 1888. In total, 28 valid species of the genus *Gasteruption* Latreille have been examined during this study; six are new species and described in this paper and eight species are new for China (Table 2). The actual number will be higher because several species from neighbouring countries can be expected to occur also in China. All known species of *Gasteruption* from China are revised, illustrated and keyed. Unfortunately, several of the species have males as holotypes and the association with the female is sometimes problematic. We compared all available females with these types and made the best fit, but this is provisional until both sexes are known from the type localities and their DNA shows that they are conspecific.

**Table 1.** List of the Chinese species of *Gasteruption* before this study.

**Species**	**Distribution**
*Gasteruption amoyense* Pasteels, 1958	Fujian (Oriental)
*Gasteruption bihamatum* Kieffer, 1911	Fujian (Oriental)
*Gasteruption corniculigerum* Enderlein, 1913	Taiwan (Oriental)
*Gasteruption curiosum* Pasteels, 1958	Hong Kong (Oriental)
*Gasteruption formosanum* Enderlein, 1913	Taiwan (Oriental)
*Gasteruption obscuripenne* Pasteels, 1958	Hainan (Oriental)
*Gasteruption oriplanum* Kieffer, 1911	Tibet (Palaearctic)
*Gasteruption parvicollarium* Enderlein, 1913	Taiwan (Oriental)
*Gasteruption poecilothecum* Kieffer, 1911	Heilongjiang (Palaearctic)
*Gasteruption rufescenticorne* Enderlein, 1913	Taiwan (Oriental)
*Gasteruption sinarum* Kieffer, 1911	Beijing (Palaearctic)
*Gasteruption sinense* Kieffer, 1924	Shanghai (Palaearctic)
*Gasteruption sinense* var. *minus* Kieffer, 1924	Shanghai (Palaearctic)
*Gasteruption sinicola* (Kieffer, 1924)	Jiangsu (Palaearctic)
*Gasteruption terebrelligerum* Enderlein, 1913	Taiwan (Oriental)
*Gasteruption varipes* (Westwood, 1851)	Hainan (Oriental)

**Table 2.** List of the Chinese species of *Gasteruption* after this study.

**Species**	**Distribution in China**
*Gasteruption amoyense* Pasteels, 1958 (*Gasteruption curiosum* Pasteels, 1958, syn. n.)	Zhejiang, Fujian, Hong Kong, Hunan (Oriental)
*Gasteruption angulatum* sp. n.	Henan, Shaanxi, Hubei (Palaearctic), Zhejiang (Oriental)
*Gasteruption assectator* (Linnaeus, 1758)	Heilongjiang, Jilin, Inner Mongolia, Xinjiang, Beijing, Hebei, Henan, Shanxi, Ningxia, Qinghai, Hubei, Tibet (Palaearctic), Hunan, Sichuan (Oriental)
*Gasteruption assectoides* sp. n.	Hubei (Palaearctic)
*Gasteruption bimaculatum* Pasteels, 1958	Henan, Tibet (Palaearctic), Fujian, Hainan, Guangxi, Yunnan (Oriental)
*Gasteruption birmanense* Pasteels, 1958	Hunan, Sichuan (Oriental)
*Gasteruption coloratum* sp. n.	Xinjiang (Palaearctic)
*Gasteruption corniculigerum* Enderlein, 1913	Zhejiang, Fujian, Taiwan, Guangdong, Guangxi, Hainan, Hunan, Guizhou (Oriental)
*Gasteruption dilutum* Semenov, 1892	Xinjiang (Palaearctic)
*Gasteruption dimidiatum* Semenov, 1892	Xinjiang (Palaearctic)
*Gasteruption formilis* Alekseev, 1995	Gansu, Xinjiang (Palaearctic)
*Gasteruption formosanum* Enderlein, 1913	Hubei (Palaearctic), Zhejiang, Taiwan (Oriental)
*Gasteruption japonicum* Cameron, 1888 (*Gasteruption sinense* var. *minus* Kieffer, 1924 syn. n.)	Heilongjiang, Jilin, Inner Mongolia, Shaanxi, Ningxia, Gansu, Xinjiang, Hubei (Palaearctic), Zhejiang, Fujian, Taiwan, Hunan, Sichuan, Yunnan (Oriental)
*Gasteruption latitibia* sp. n.	Hubei (Palaearctic), Fujian, Hunan, Guizhou (Oriental)
*Gasteruption oriplanum* Kieffer, 1911	Tibet (Palaearctic)
*Gasteruption parvicollarium* Enderlein, 1913	Ningxia, Shanxi, Hebei, Hubei, Jiangsu (Palaearctic), Zhejiang, Fujian, Taiwan, Hunan, Guangxi, Guizhou (Oriental)
*Gasteruption poecilothecum* Kieffer, 1911	Heilongjiang, Jilin, Hebei (Palaearctic)
*Gasteruption rufescenticorne* Enderlein, 1913	Ningxia, Hubei, Jiangsu (Palaearctic), Zhejiang, Fujian, Taiwan, Hunan, Guangxi, Hainan (Oriental)
*Gasteruption sinarum* Kieffer, 1911 (*Gasteruption sinense* Kieffer, 1924, syn. n.)	Liaoning, Inner Mongolia, Beijing, Tianjin, Jiangsu, Shanghai, Ningxia, Henan, Anhui, Hubei (Palaearctic), Zhejiang, Hunan, Guizhou, Guangxi, Guangdong (Oriental)
*Gasteruption sinepunctatum* sp. n.	Jilin, Tibet (Palaearctic), Zhejiang, Taiwan (Oriental)
*Gasteruption sinicola* (Kieffer, 1924)	Ningxia, Jiangsu (Palaearctic), Fujian, Guangdong, Hainan, Hunan (Oriental)
*Gasteruption strigosum* sp. n.	Fujian, Hainan, Yunnan (Oriental)
*Gasteruption subhamatum* Pasteels, 1958	Hainan (Oriental)
*Gasteruption terebrelligerum* Enderlein, 1913	Shanxi, Hubei (Palaearctic), Fujian, Taiwan, Hunan, Yunnan (Oriental)
*Gasteruption tonkinense* Pasteels, 1958	Jiangsu (Palaearctic), Zhejiang, Fujian, Guangxi, Guizhou (Oriental)
*Gasteruption tournieri* Schletterer, 1885	Jilin, Henan (Palaearctic), Hunan, Guizhou (Oriental)
*Gasteruption transversiceps* Pasteels, 1958	Hainan (Oriental)
*Gasteruption varipes* (Westwood, 1851)	Fujian, Taiwan, Hainan, Yunnan (Oriental)

## Material and methods

Descriptions of the species are a combination of comparative illustrations and a short diagnosis and description, followed by a summary of the distribution (only based on examined specimens unless otherwise indicated) and biology (as far as known with at least the phenology given). Descriptions were made under an Olympus SZ61 stereoscope, in combination with a 40W LED lamp. Photographic images were made with an Olympus motorized stereomicroscope SZX12 and Stemi2000-CS; the images were processed with both Image-Pro Plus and AnalySIS Extended Focal Imaging software, and plates were finished with ACDSee10.0 and Photoshop CS 8.0.1, mostly to adjust the size and background.

**Figure 1.**
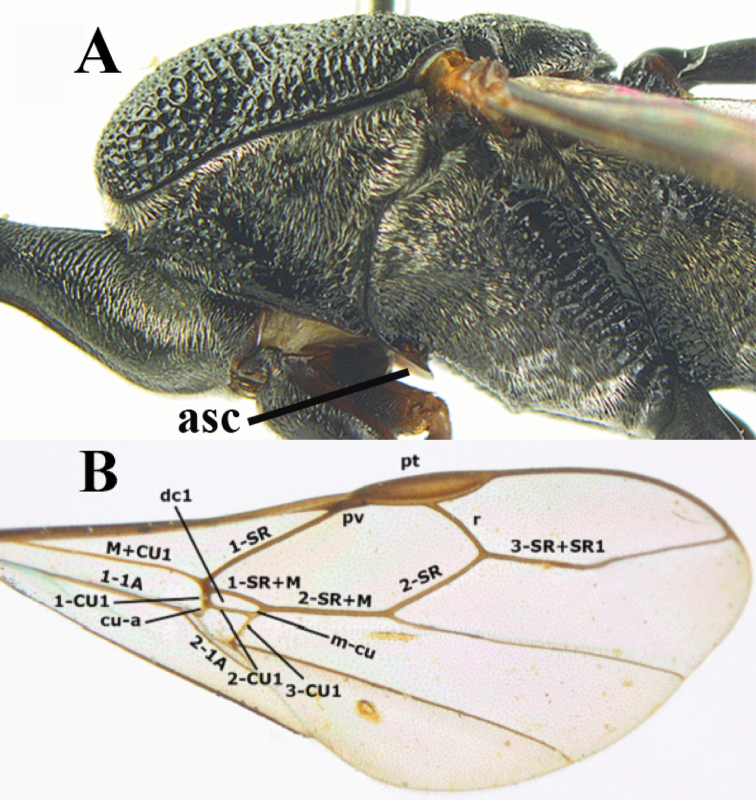
Mesosoma (**A**, from van Achterberg) and fore wing (**B**) of Gasteruptiidae. **asc** = antesternal carina; **pt** = pterostigma; **pv** = parastigmal vein; **dc1** = first discal cell.

Morphology. The term “antesternal carina” is coined for the more or less upcurved anterior border of the mesosternum *sensu lato* (Fig. 1A; Fig. 3). It is situated above the fore coxae and varies from a narrow non-lamelliform carina to a very wide and upcurved lamella. For other terminology used in this paper, see van Achterberg (1988, 1993) and Hong et al. (2011). Measurements are taken as indicated by van Achterberg (1988). Additional non-exclusive characters in the key are between brackets.

Material. Types and other specimens have been examined from the following institutions:

BMNHNatural History Museum, London (Mr D. Notton).


CAUInsect Collection, China Agricultural University, Beijing (Prof. Xin-li Wang, Prof. Dr Wan-zhi Cai and Prof. Dr Ding Yang).


CSCSCentral South University of Forestry and Technology, Changsha (Prof. Dr Mei-cai Wei).


DEIDeutsche Entomologische Institut Senckenberg, Müncheberg (Dr A. Taeger, Mr A. Liston).


ECUSEntomology Collection of University of Sapporo, Sapporo (Dr M. Ohara).


KBINKoninklijk Belgisch Instituut voor Natuurwetenschappen, Brussels (Mr Y. Gerard).


MNHNMuseum National d’Histoire Naturelle, Paris (Dr C. Villemant, Mrs A. Touret-Alby).


NHMSNatural History Museum, Stockholm (Dr H. Vårdal, Dr S. Klopfstein).


NMNSNational Museum of Natural Science, Taichung, Taiwan (Dr M.L. Chan).


RMNHNaturalis Biodiversity Center, Leiden, Netherlands (including collections from the Entomological Institute, Wageningen and former National Museum of Natural History, Leiden and Zoological Museum, Amsterdam).


SCAUHymenoptera Collection, South China Agricultural University, Guangzhou.


TARITaiwan Agricultural Research Institute-Entomology, Taichung (Dr C.F. Lee).


USNMU. S. Natural Museum (Natural History), Smithsonian Institution, Washington D.C. (Dr D. Smith).


ZISPZoological Institute, Academia NAUK, St. Petersburg (Dr S.A. Belokobylskij).


ZJUHInstitute of Insect Sciences, University of Zhejiang, Hangzhou (Prof. Jun-hua He and Prof. Dr Xue-xin Chen).


ZMIZZoological Museum, Museum and Institute of Zoology, Polish Academy of Sciences, Warsaw (Prof. Dr W. Bogdanowicz, Mrs D. Mierzwa-Szymkowiak).


## Systematics

### Gasteruptiidae

Ashmead, 1900

http://species-id.net/wiki/Gasteruptiidae

#### Diagnosis.

Body length between 5.0–20.0 mm; female antenna 14-segmented, male 13-segmented, very rarely 14-segmented; eye relatively long, extending almost to mandible; mandibles short and not broadly overlapping when closed; maxillary palp 6-segmented, labial palp 4-segmented; propleuron neck-like, more or less as long as mesoscutum in front of tegulae; propodeum with or without longitudinal carina; fore wing with discal cell small or absent; trochantellus of hind leg distinctly differentiated, hind tibia (or metatibia) clavate; metasoma inserted very high on propodeum; ovipositor varies from not exposed (in *Pseudofoenus*) to more than twice as long as the body (Jennings and Austin 2002; Macedo 2009; 2011).

Gasteruptiidae includes two extant subfamilies, Hyptiogastrinae and Gasteruptiinae. Gasteruptiinae is distinguished from Hyptiogastrinae mainly by mandible short, prefemur present, female subgenital sternite notched or slit (Jennings and Austin 2002).

In China only the genus *Gasteruption* belonging to the subfamily Gasteruptiinae occurs.

### Gasteruption

Latreille, 1796

http://species-id.net/wiki/Gasteruption

Gasteruption Latreille, 1796: 113; Pagliano 2008: 202. Type-species (designated by Latreille 1810): *Ichneumon assectator* Linnaeus, 1758.
Gasteruptron Westwood, 1840: 56. Invalid emendation.
Gastryptium Agassiz, 1846: 159. Invalid emendation.
Gasteryption Schletterer, 1890: 375. Invalid emendation.
Gasteruptia Dominique, 1893: 198. Invalid emendation.
Gasteruptium Schulz, 1906: 133. Invalid emendation.
Gastrhyptium Schulz, 1911: 55. Invalid emendation.
Foenus Fabricius, 1798: 240; Pagliano 2008: 197. Type-species (designated by Latreille 1810): *Ichneumon jaculator* Linnaeus, 1758. Curtis (1832) designated *Ichneumon assectator* Linnaeus, 1758, as type-species and this was considered valid by Pagliano (2008: 197). Westwood (1840) considered correctly *Ichneumon jaculator* Linnaeus to be the type-species as designated by Latreille (1810).
Faenus Abeille de Perrin, 1879: 260, 262. Invalid emendation.
Phoenus Schletterer, 1890: 375. Invalid emendation.
Rhydinofoenus Bradley, 1909: 38; Pagliano 2008: 388. Type-species (by original designation): *Rhydinofoenus kaweahensis* Bradley, 1909.
Dolichofoenus Kieffer, 1910: 77; Pagliano 2008: 162. Type-species (by original designation): *Foenus raphidioides* Westwood, 1851.
Trichofoenus Kieffer, 1910: 77; Pagliano 2008: 436. Type-species (by original designation): *Gasteruption merceti* Kieffer, 1904.

#### Description.

Body length 5.0–20.0 mm.

*Head*. Head directly or gradually narrowed behind eyes and curved laterally; vertex convex posteriorly (but flat in e.g., *Gasteruption bimaculatum*); occipital carina developed to absent; antenna of female 14-segmented, male 13-segmented; eye large, glabrous or setose; clypeus without longitudinal striae or ridge; mandible short and robust, overlapping slightly when closed, with a large laterally directed basal tooth, subapical tooth small or absent; malar space present; maxillary palpi 6-segmented, labial palpi 4-segmented.

*Mesosoma*. Propleuron more or less elongate; pronotum divided in three lobes separated by curved crenulate notauli, antero-lateral pronotal tooth present or absent; anteriorly mesonotum in lateral view rounded or truncate (*Gasteruption varipes*); lateral lobes of mesoscutum usually less sculptured than middle lobe; propodeum sculptured, medio-longitudinal carina or elevation distinct or indistinct.

*Legs*. Trochantellus only present on hind leg, but usually incompletely differentiated; hind trochanter with oblique subapical groove; hind tibia clavate.

*Wings*. Fore wing (Fig. 1B): jugal lobe absent, vein r-m absent; discal cell generally present (rarely absent, see Fig 271), formed by junction of 1-SR+M, 1-CU, 2-CU and 1m-cu, vein 1-SR+M arising from junction of M+CU1 and 1-SR; basal third of vein 2-M tubular and remainder only pigmented. Hind wing with 3–4 hamuli; vein M+CU, 1-CU and r-m usually partly visible.

*Metasoma*. Metasoma inserted very high on propodeum; first metasomal tergite ventrally almost closed, concealing most of first sternum, except posteriorly; second tergite immovably joined to first tergite and widely open ventrally; female hypopygium with a deep slit-like Y-shaped or a shallow V-shaped notch; ovipositor from shorter than metasoma to longer than body length, apex of ovipositor sheath black, dark brown, pale yellow, ivory or white.

##### Key to species of the genus *Gasteruption* Latreille from China

**Table:** 

1	First discal cell of fore wing absent (Fig. 271); third antennal segment of both sexes about 3 times as long as wide; hind tibia weakly inflated (Fig. 268)	*Gasteruption subhamatum* Pasteels, 1958
–	First discal cell of fore wing present (Figs 16, 77, 117, 159, 180); third antennal segment of both sexes about 1.3–2.5 times as long as wide; hind tibia variable (Figs 13, 27, 74, 114, 156, 177, 203)	2
2	Ovipositor present; antenna with 14 segments; (females, species of which no females have been examined are provisionally inserted)	3
–	Ovipositor absent; antenna with 13 segments; (males; species of which no males have been examined are provisionally included in the key)	29
3	Apex of ovipositor sheath black or dark brown, if rather yellow or brown apically then pale apical part less than 0.4 times as long as hind basitarsus	4
–	Apex of ovipositor sheath distinctly white or ivory (but rarely pale brown), 0.3–8.0 times as long as hind basitarsus, but brown-yellow or brown and 0.3–1.0 times as long as hind basitarsus in *Gasteruption sinarum*	17
4	Ovipositor sheath 0.3–1.7 times as long as hind tibia and tarsus combined, at most 0.8 times as long as metasoma and 0.6–2.7 times as long as hind tibia; hypopygium comparatively shallowly emarginate posteriorly; occipital carina obsolescent to narrowly lamelliform medio-dorsally (Figs 29, 116, 129, 158, 170, 179, 246, 257, 278)	5
–	Ovipositor sheath 2.0–3.9 times as long as hind tibia and tarsus combined, 1.0–1.9 times as long as metasoma and 3.0–7.0 times as long as hind tibia; hypopygium more or less deeply incised, often slit-like; occipital carina variable, obsolescent or lamelliform medio-dorsally (Figs 53, 76, 108, 322)	13
5	Malar space about as long as second antennal segment (= pedicel); posteriorly propodeum with median carina distinctly stronger developed than surrounding rugulosity, but carina may be largely absent; first discal cell of fore wing slightly sinuate posteriorly (♂ Figs 171, 172)	*Gasteruption oriplanum* Kieffer, 1911
–	Malar space 0.1–0.3 times as long as second antennal segment (= pedicellus); median carina of propodeum absent (but sometimes a slightly elevated smooth median line), if present then surrounding reticulate-rugose and carina similarly developed	6
6	Interspaces of mesoscutum very finely and densely coriaceous and usually with isolated punctures (but sometimes entirely coriaceous in *Gasteruption parvicollarium* and *latitibia*); vertex and frons more or less shiny and largely smooth (Figs 129, 158, 246) or very finely punctulate; propleuron 0.8–1.0 times as long as mesoscutum in front of tegulae and moderately slender (Figs 125, 154, 175, 242); hind basitarsus comparatively short and black (Figs 127, 156, 244)	7
–	Interspaces of mesoscutum more or less irregularly coriaceous and without punctures or densely transversely rugulose or rugose; vertex and frons matt and more or less coriaceous (Figs 29, 116, 257, 278); propleuron 0.7–1.6 times as long as mesoscutum in front of tegulae, robust; head transverse (Figs 29, 116, 278); hind basitarsus usually longer and colour variable (Figs 255, 276)	10
7	Hind tibia comparatively slender (Fig. 177); side of pronotum with narrow and weakly crenulate grooves (Fig. 175); propleuron granulate antero-dorsally; hind basitarsus slenderer (Fig. 177); vertex distinctly punctulate or very finely coriaceous	*Gasteruption parvicollarium* Enderlein, 1913
–	Hind tibia inflated (Figs 127, 156, 244); side of pronotum with moderately wide to wide and distinctly crenulate grooves (Figs 125, 154, 242); propleuron rugulose antero-dorsally; hind basitarsus less slender (Figs 127, 156, 244); vertex virtually smooth	8
8	Mesoscutum predominantly finely coriaceous, at most with some shallow punctures (Fig. 155); head more protruding in lateral and anterior view (Figs 153, 157); head truncate medio-posteriorly or nearly so (Fig. 158); third antennal segment 1.7–1.9 times as long as second segment *Gasteruption latitibia* sp. n.
–	Mesoscutum coarsely (often “crater”-like) punctate (Figs 126, 243); head less protruding in lateral and anterior view (Figs 124, 128, 241, 245); head distinctly emarginate medio-posteriorly (Figs 129, 246); third antennal segment 1.5–2.0 times as long as second segment	9
9	Hind tibia about as long as hind femur and trochanter or slightly longer (Fig. 127); head somewhat longer in dorsal (Fig. 129) and lateral (Fig. 124) view; head directly narrowed behind eyes in dorsal view (Fig. 129)	*Gasteruption formosanum* Enderlein, 1913
–	Hind tibia 1.1–1.2 times as long as hind femur and trochanter (Fig. 244); head somewhat shorter in dorsal (Fig. 246) and lateral (Fig. 241) view; head roundly narrowed behind eyes in dorsal view (Fig. 246)	*Gasteruption sinicola* (Kieffer, 1924)
10	Clypeus with large shallow depression up to dorsal third (Fig. 115); apical antennal segment 1.4–1.5 times as long as third antennal segment; hind basitarsus comparatively short and wide (Fig. 114); mesoscutum rather finely and very irregularly rugulose or rugose (Fig. 113; males have rugose, females anteriorly rugulose)	*Gasteruption formilis* Alekseev, 1995
–	Clypeus at most with a small depression; apical antennal segment at most 1.2 times as long as third antennal segment and its colour similar to colour of medial segments; hind basitarsus comparatively long and narrow (Figs 27, 255, 276)	11
11	Propleuron slender and 1.3–1.6 times as long as mesoscutum in front of tegulae (Fig. 253); head conically narrowed behind eyes (Fig. 257); vertex very superficially punctulate and with satin sheen (Fig. 257); ovipositor at least 0.6 times as long as metasoma	*Gasteruption strigosum* sp. n.
–	Propleuron robust and 0.7–0.9 times as long as mesoscutum in front of tegulae (Figs 25, 274); head more gradually narrowed behind eyes (Figs 29, 278); vertex densely micro-sculptured and matt (Figs 29, 278); ovipositor 0.3–0.5 times as long as metasoma	12
12	Head comparatively wide behind eyes in dorsal view (Fig. 29); OOL 1.3–1.4 times as long as diameter of posterior ocellus (Fig. 29); third antennal segment 1.5–1.7 times as long as second segment; mesopleuron below precoxal sulcus more or less coriaceous or with fine rugulae (Fig. 25)	*Gasteruption assectator* (Linnaeus, 1758)
–	Head distinctly narrowed behind eyes in dorsal view (Fig. 278); OOL about as long as diameter of posterior ocellus (Fig. 278); third antennal segment 1.7–1.9 times as long as second segment; mesopleuron below precoxal sulcus more or less rugose-reticulate (Fig. 274), but sometimes mainly coriaceous	*Gasteruption terebrelligerum* Enderlein, 1913
13	Vertex of ♀ medio-posteriorly reversed V-shaped emarginate and flat (Fig. 53); vertex smooth, shiny dorsally and long (Fig. 53)	*Gasteruption bimaculatum* Pasteels, 1958
-	Vertex of ♀ truncate medio-posteriorly (Fig. 76) or reversed U-shaped emarginate, shorter and moderately convex (Figs 103, 108); if vertex more or less emarginate, then vertex finely sculptured, with satin sheen and shorter (Fig. 322)	14
14	Head reversed U-shaped emarginate in dorsal view (Fig. 108); first metasomal segment orange-brown or black; propodeum finely reticulate	*Gasteruption dimidiatum* Semenov, 1892
–	Head truncate medio-posteriorly or nearly so (Figs 76, 322; cf. ♂ Fig. 101); first metasomal segment black or dark brown; propodeum coarsely reticulate	15
15	Hind coxa and femur yellow- or orange-brown; metasoma yellow-brown; mesoscutum rather densely setose laterally (Fig. 73); mesopleuron black in general and reticulate (Fig. 72)	16
–	Hind coxa and femur dark brown or black (Fig. 320); brown in old bleached specimens; metasoma largely dark brown or black; mesoscutum less densely setose (Fig. 319); mesopleuron red-brown and coarsely vermiculate-reticulate (Fig. 318; but rarely only weakly so)	*Gasteruption varipes* (Westwood, 1851)
16	Hind tibia orange-brown, at most basally dark brown and more swollen (cf. ♂ Fig. 102); hind basitarsus and second segment entirely dark brown (cf. ♂ Fig. 102); head at least partly red-brown dorsally	*Gasteruption dilutum* Semenov, 1892
–	Hind tibia black and subbasally ivory and less swollen (Fig. 74); hind basitarsus mainly ivory and only basally dark brown and second segment medially ivory (Fig. 74); head black dorsally	*Gasteruption coloratum* sp. n.
17	Head with 1–3 depressions in front of occipital carina, but sometimes shallowly impressed or obsolescent (*Gasteruption rufescenticorne*) then occipital carina medium-sized and distinctly lamelliform and more or less depressed in front of carina (Figs 84, 207, 300)	18
–	Head flat or evenly convex in front of occipital carina, if with a shallow depression in front of occipital carina then occipital carina narrow and hardly or not lamelliform	20
18	Medial depression of vertex comparatively wide, with both lateral depressions in front of occipital carina distinctly impressed (Fig. 300); posterior half of mesoscutum transversely or obliquely rugulose (Fig. 297), in large specimens transversely rugose; propleuron 0.8–1.0 times as long as mesoscutum in front of tegulae; hind basitarsus partly ivory; ivory part of ovipositor sheath 1.8–2.5 times as long as hind basitarsus	*Gasteruption tournieri* Schletterer, 1885
–	Medial depressions of vertex medium-sized and lateral depressions in front of occipital carina not or superficially impressed (Figs 84, 207); posterior half of mesoscutum finely coriaceous or coarsely punctate-rugose (Figs 81, 202); propleuron 1.0–1.2 times as long as mesoscutum in front of tegulae; hind basitarsus partly white or ivory (Fig. 82) or entirely dark brown or black (Fig. 203); ivory part of ovipositor sheath 1.0–4.0 times as long as hind basitarsus	19
19	Head in dorsal view gradually roundly narrowed (Fig. 207); hind basitarsus entirely dark brown or black; medio-posteriorly mesoscutum matt and mainly finely coriaceous, at most with some large punctures; mandible largely dark brown or black; ivory or yellow part of ovipositor sheath 1.0–2.1 times as long as hind basitarsus	*Gasteruption rufescenticorne* Enderlein, 1913
–	Head in dorsal view linearly narrowed (Fig. 84); hind basitarsus with ivory band (Fig. 82), rarely, an ivory band absent, sometimes also second and third segments ivory; medio-posteriorly mesoscutum distinctly punctate-rugose or rugose; mandible usually brown-yellow or brown; ivory or yellow part of ovipositor sheath 2.8–4.0 times as long as hind basitarsus	*Gasteruption corniculigerum* Enderlein, 1913
20	Ovipositor sheath comparatively wide and about 0.9 times as long as hind tibia, 0.3 times as long as metasoma and 0.2 times as long as body; middle lobe rather protuberant	*Gasteruption assectoides* sp. n.
–	Ovipositor sheath comparatively narrow and 1.1–9.0 times as long as hind tibia, 0.6–2.8 times as long as metasoma and 0.4–2.0 times as long as body; middle lobe less protuberant (Figs 64, 143, 192, 218, 287)	21
21	Ovipositor sheath 1.8–2.0 times as long as body, 8–9 times as long as hind tibia and 2.7–2.8 times as long as metasoma; ivory apical part of ovipositor sheath 5–8 times as long as hind basitarsus; frons rugose	*Gasteruption transversiceps* Pasteels, 1958
–	Ovipositor sheath 0.4–1.3 times as long as body and 0.6–1.9 times as long as metasoma; pale apical part of ovipositor sheath 0.3–4.0 times as long as hind basitarsus; frons smooth or micro-sculptured	22
22	Ovipositor sheath about 0.4 times as long as body and 0.6 times as long as metasoma; ovipositor widened apically and more or less angularly upcurved apically in dead specimens (Fig. 18)	*Gasteruption angulatum* sp. n.
–	Ovipositor sheath 0.8–1.3 times as long as body and 1.2–1.9 times as long as metasoma; ovipositor slender and nearly straight or gradually upcurved apically	23
23	Pale apical part of ovipositor sheath 3.0–3.5 times as long as hind basitarsus; vertex shiny and largely smooth or finely punctulate; fourth antennal segment of ♀ 1.7–2.3 times as long as third antennal segment; mesoscutum more or less coarsely spaced punctate medio-posteriorly (Fig. 288)	*Gasteruption tonkinense* Pasteels, 1958
–	Pale apical part of ovipositor sheath 0.3–3.0 times as long as hind basitarsus; if 2.8–3.0 times then head dorsally with satin sheen and distinctly finely sculptured (Fig. 68); fourth antennal segment of ♀ 1.2–1.9 times as long as third antennal segment; mesoscutum coriaceous with punctures and medio-posteriorly some transverse rugae (Fig. 230), punctate-rugose or partly reticulate (Figs 4, 144, 193, 219)	24
24	Brown-yellow or brown apical part of ovipositor sheath 0.3–1.0 times as long as hind basitarsus; subbasal pale part of hind tibia hardly differentiated (Fig. 220); vertex distinctly convex medio-posteriorly in lateral view and occipital carina non-lamelliform medio-dorsally (Fig. 217); mesoscutum coarsely punctate (Fig. 219)	*Gasteruption sinarum* Kieffer, 1911
–	Pale apical part of ovipositor sheath 1.3–3.0 times as long as hind basitarsus; subbasal pale patch of hind tibia distinctly differentiated; vertex slightly convex medio-posteriorly in lateral view or occipital carina narrow lamelliform medio-dorsally (Figs 2, 63, 228); mesoscutum less coarsely punctate (Figs 144, 230) or rugulose	25
25	Anterior half of mesoscutum very finely coriaceous, rarely very finely transversely rugulose, and with at most some shallow punctures; head dorsally more or less shiny and smooth; head directly narrowed posteriorly in dorsal view (Figs 147, 233)	26
–	Anterior half of mesoscutum distinctly less finely coriaceous and with distinct punctures; head dorsally with satin sheen and micro-sculptured; head less narrowed in dorsal view (Figs 7, 68, 196)	27
26	Fourth antennal segment of ♀ 1.3–1.5 times as long as third antennal segment (cf. ♂ Fig. 151); head somewhat less narrowed in dorsal view, resulting in a less transverse head (Fig. 147); mesoscutum usually more punctate (Fig. 144)	*Gasteruption japonicum* Cameron, 1888
–	Fourth antennal segment of ♀ 1.7–1.9 times as long as third antennal segment (Fig. 236); head more narrowed in dorsal view, resulting in a more transverse head (Fig. 233); mesoscutum usually hardly or not punctate (Fig. 230)	*Gasteruption sinepunctatum* sp. n.
27	Pale apical part of ovipositor sheath 2.8–3.0 times as long as hind basitarsus; occipital carina medio-dorsally narrow and non-lamelliform (Fig. 63); OOL about 1.6 times diameter of posterior ocellus (Fig. 68)	*Gasteruption birmanense* Pasteels, 1958
–	Pale apical part of ovipositor sheath 1.3–2.3 times as long as hind basitarsus; occipital carina medio-dorsally narrowly to medium-sized lamelliform (Figs 2, 191); OOL 1.1–1.4 times diameter of posterior ocellus (Figs 7, 196), but 1.6 times in *Gasteruption amoyense*	28
28	Head elliptical in dorsal view (cf. Fig. 7); eyes more elongate elliptical in anterior view (cf. Fig. 6)	*Gasteruption amoyense* Pasteels, 1958
–	Head trapezoid in dorsal view (Fig. 196); eyes somewhat less elongate in anterior view (Fig. 195)	*Gasteruption poecilothecum* Kieffer, 1911
29	Head with 1–3 distinct depressions or a groove with two minute tubercles in front of distinctly lamelliform occipital carina (Figs 92, 216, 304), but depression may be small (*Gasteruption tonkinense*, *Gasteruption rufescenticorne*)	30
–	Head flat or evenly convex in front of occipital carina, without any depression; occipital carina usually narrow (Figs 47, 260; cf. ♀ Fig. 265)	33
30	Medio-dorsal depression in front of occipital carina absent or nearly so; occipital carina comparatively narrow (cf. Figs 286, 292)	*Gasteruption tonkinense* Pasteels, 1958
–	Medio-dorsal depression in front of occipital carina shallow and medium-sized to deep and large; occipital carina comparatively wide (Figs 92, 216, 304)	31
31	Medial depressions comparatively deep, large and lateral depressions in front of occipital carina distinctly impressed (Fig. 304); posterior half of mesoscutum transversely or obliquely rugulose (Fig. 305), in large specimens transversely rugose	*Gasteruption tournieri* Schletterer, 1885
–	Medial depressions of vertex comparatively shallow, medium-sized and lateral depressions in front of occipital carina not or superficially impressed (Figs 92, 216); posterior half of mesoscutum finely coriaceous or coarsely punctate-rugose (Figs 89, 211)	32
32	Head gradually roundly narrowed behind eyes and medio-posteriorly semi-circularly emarginate (Fig. 215); anterior half of mesoscutum matt and mainly coriaceous, with some minute punctures; occipital carina medium-sized (Fig. 209); lateral depressions in front of occipital carina absent (Fig. 216)	*Gasteruption rufescenticorne* Enderlein, 1913
–	Head linearly narrowed behind eyes, trapezoid and medio-posteriorly truncate (Fig. 92); anterior half of mesoscutum with satin sheen and densely finely rugulose, punctate (Fig. 89) or punctate-rugose; occipital carina comparatively wide (Fig. 87); lateral depressions in front of occipital carina shallowly impressed (Fig. 92) or obsolescent	*Gasteruption corniculigerum* Enderlein, 1913
33	First discal cell of fore wing absent (cf. ♀ Fig. 271)	*Gasteruption subhamatum* Pasteels, 1958
–	First discal cell of fore wing present (Figs 58, 98, 140, 171, 328)	34
34	Head distinctly emarginate medio-posteriorly (Figs 61, 139); posteriorly vertex flat in lateral view (but less so in *Gasteruption formosanum* and *Gasteruption dimidiatum*), smooth or finely sculptured, shiny and comparatively long (Figs 55, 61)	35
–	Head subtruncate medio-posteriorly; posteriorly vertex near occipital carina moderately convex in lateral view (Figs 166, 182, 325; cf. ♀ Figs 10, 63, 111, 142, 153, 217, 228, 241, 273, 309); if more or less emarginate, then vertex finely sculptured, with satin sheen, and shorter (Figs 19, 33, 101, 119, 251)	37
35	Hind tibia about as long as hind femur and trochanter and distinctly swollen (Fig. 136); mesoscutum densely and coarsely punctate and interspaces coriaceous (Fig. 135)	*Gasteruption formosanum* Enderlein, 1913
–	Hind tibia 1.1–1.2 times as long as hind femur and trochanter and less swollen (Fig. 62; cf. ♀ Fig. 106); mesoscutum regularly transversely rugose or rugulose, sometimes sculpture obsolescent or punctate and interspaces partly smooth	36
36.	Head comparatively short in dorsal view (cf. ♀ Fig. 108); mesoscutum coarsely punctate and no rugae (cf. ♀ Fig. 105); first metasomal segment orange-brown; posteriorly vertex rather convex in lateral view (cf. ♀ Fig. 103)	*Gasteruption dimidiatum* Semenov, 1892
–	Head long in dorsal view (Fig. 61); mesoscutum irregularly transversely rugose or rugulose, sometimes very superficially so; first metasomal segment black or dark brown; posteriorly vertex flat in lateral view (Fig. 55)	*Gasteruption bimaculatum* Pasteels, 1958
37	Pronotum and mesopleuron very coarsely vermiculate-reticulate and mesoscutum laterally more or less dark red-brown; temple nearly as long as eye in dorsal view or longer (Fig. 331)	*Gasteruption varipes* (Westwood, 1851)
–	Pronotum and at least dorsal half of mesopleuron largely coriaceous and usually black; temple shorter than eyes (Figs 119, 152, 239, 251) or subequal (Figs 187, 260)	38
38	Hind basitarsus comparatively short to medium-sized and somewhat wider (Figs 123, 189, 249)	39
–	Hind basitarsus comparatively long and narrow (Figs 23, 36, 102, 150, 169, 225, 240, 263)	41
39	Mesoscutum finely coriaceous or rugulose, without small punctures; head long in dorsal view (Fig. 187); notauli narrow (Fig. 184)	*Gasteruption parvicollarium* Enderlein, 1913
-	Mesoscutum distinctly punctate or irregularly rugose (Fig. 120; cf. ♀ Fig. 243); head shorter in dorsal view (Figs 119, 251); notauli moderately wide (Fig. 120; cf. ♀ Fig. 243)	40
40	Mesoscutum “crater-like” punctate (cf. ♀ Fig. 243); clypeus without distinct depression (cf. ♀ Fig. 245)	*Gasteruption sinicola* (Kieffer, 1924)
–	Mesoscutum irregularly rugose (Fig. 120); clypeus more or less triangularly depressed ventrally (Fig. 122)	*Gasteruption formilis* Alekseev, 1995
41	Mesoscutum very finely and densely coriaceous, elongate (similar to*Gasteruption parvicollarium* but more evenly convex)and propleuron 0.9–1.0 times as long as mesoscutum in front of tegulae	42
–	Mesoscutum more coarsely sculptured or largely smooth, if densely coriaceous then propleuron 0.7–0.8 times as long as mesoscutum in front of tegulae (Figs 167, 168)	43
42	Third antennal segment normal, slender and distinctly longer than fourth segment (Fig. 151); head some less narrowed in dorsal view, resulting in a less transverse head (Fig. 152); mesoscutum usually more punctate (cf. ♀ Fig. 144)	*Gasteruption japonicum* Cameron, 1888
–	Third antennal segment shortened, wide and hardly longer than fourth segment (Fig. 238); head more narrowed in dorsal view, resulting in a more transverse head (Fig. 239); mesoscutum usually hardly or not punctate (cf. ♀ Fig. 230)	*Gasteruption sinepunctatum* sp. n.
43	Propleuron 1.3–1.6 times as long as mesoscutum in front of tegulae (cf. ♀ Fig. 253)	*Gasteruption strigosum* sp. n.
–	Propleuron 0.7–1.1 times as long as mesoscutum in front of tegulae (Fig. 167)	44
44	Malar space about as long as second antennal segment (= pedicellus), head in anterior view protruding below lower level of eyes by about basal width of mandible and mandibular condylus distinctly below lower level of eyes, in lateral viewcondylarincision of malar space remains far removed from eye (Fig. 166); vertex matt, micro-sculptured	*Gasteruption oriplanum* Kieffer, 1914
–	Malar space distinctly shorter than second antennal segment, head in anterior view slightly protruding below lower level of eyes by less than basal width of mandible and mandibular condylus near lower level of eyes (Figs 35, 283), in lateral viewcondylarincision of malar space close to eye (cf. ♀ Figs 24, 63, 191, 217, 273); vertex variable	45
45	Mesoscutum evenly coriaceous, without punctures of rugulae; head comparatively transverse and weakly narrowed posteriorly (Figs 19, 33)	46
–	Mesoscutum at least with some punctures or rugae posteriorly; head variable, if transverse then more or less narrowed posteriorly in dorsal view (Figs 227, 281; cf. ♀ Figs 4, 68, 196, 315)	47
46	Hind tibia distinctly swollen and at least partly dark brown (Fig. 36)	*Gasteruption assectator* (Linnaeus, 1758)
–	Hind tibia slender and largely brown (Fig. 23)	*Gasteruption angulatum* sp. n.
47	Mesoscutum largely punctate or rugose (Fig. 97; cf. ♀ Figs 65, 73, 219, 311); mesoscutum interspaces between punctures smooth or punctulate-coriaceous; mesosoma laterally and ventrally brown or red-brown	48
–	Mesoscutum finely coriaceous or densely rugulose (Figs 162, 282; cf. ♀ Figs 4, 193), at most with some shallow punctures or rugae posteriorly; mesosoma laterally and ventrally mainly black or dark brown	52
48	Frons distinctly rugulose (cf. ♀ Fig. 315)	*Gasteruption transversiceps* Pasteels, 1958
–	Frons coriaceous or largely smooth	49
49	Hind coxa and femur yellow- or orange-brown; metasoma yellow-brown; mesoscutum rather densely setose laterally (Fig. 97; cf. ♀ Fig. 73)	50
–	Hind coxa and femur dark brown or black (Fig. 225; cf. ♀ Fig. 66); metasoma largely dark brown or black; mesoscutum less densely setose (cf. ♀ Figs 65, 219)	51
50	Hind tibia more swollen, orange-brown and basally widely ivory (Fig. 102); hind basitarsus and second segment dark brown or brown (Fig. 102); head at least partly red-brown or dark brown dorsally	*Gasteruption dilutum* Semenov, 1892
–	Hind tibia less swollen, black and subbasally ivory (cf. ♀ Fig. 74); hind basitarsus mainly ivory and only basally dark brown and second segment medially ivory (cf. ♀ Fig. 74); head black dorsally	*Gasteruption coloratum* sp. n.
51	Eyes comparatively elongate (cf. ♀ Fig. 217)	*Gasteruption sinarum* Kieffer, 1911
–	Eyes less elongate (cf. ♀ Fig. 63)	*Gasteruption birmanense* Pasteels, 1958
52	Frons and vertex shiny and smooth; mesoscutum with some punctures medio-posteriorly (Fig. 162)	*Gasteruption latitibia* sp. n.
–	Frons and vertex matt or with satin sheen, micro-sculptured; mesoscutum with some rugae medio-posteriorly (cf. ♀ Figs 4, 193), but punctate in *Gasteruption terebrelligerum*	53
53	Mesoscutum coriaceous and only posteriorly some punctures (Fig. 282); head directly narrowed (Fig. 281)	*Gasteruption terebrelligerum* Enderlein, 1913
–	Mesoscutum distinctly transversely rugose medio-posteriorly (cf. ♀ Figs 4, 193); head gradually narrowed (cf. ♀ Figs 7, 196)	54
54	Head elliptical in dorsal view (cf. ♀ Fig. 7); eyes more elongate elliptical (cf. ♀ Fig. 2)	*Gasteruption amoyense* Pasteels, 1958
–	Head trapezoid in dorsal view (cf. ♀ Fig. 196); eyes somewhat less elongate (cf. ♀ Fig. 191)	*Gasteruption poecilothecum* Kieffer, 1911

### Gasteruption amoyense

Pasteels, 1958

http://species-id.net/wiki/Gasteruption_amoyense

Figs 2–9

Gasteruption amoyense Pasteels, 1958: 178–179, fig. 4.
Gasteruption curiosum Pasteels, 1958: 177–178, fig. 3. syn. n.

#### Type material.

Holotype of *Gasteruption amoyense*, ♀ (USNM), “[China:] Amoy [= Xiamen]”, “S.F. Light coll.”, “Holotype”, “*Gasteruption amoyense* n. sp., J. Pasteels det., 1955”; Holotype of *Gasteruption curiosum*, ♀ (BMNH), “Type”, “[China:], Hongkong, F.W. Terry, 1911–359”, “*Gasteruption curiosum* n. sp., J. Pasteels det., 1954”, “Holotype”, “B.M. Type Hym. 3a.365”.

#### Additional material.

1 ♀ (ZJUH), “[China:] Zhejiang, Hangzhou, Ru-zuo Zhu”; 1 ♀ (CSCS) “[China:] Fujian, Mt. Wuyi, Guadun, 18.V.2004, 1000–1500 m, Hu Zhou”; 1 ?♀ (CSCS), “[China:] Hunan, Huaihua, Xupu, Dajiangkou, 18.V.1982”; 1 ♀ (CSCS) “[China:], Hunan, Taoyuandong, 10.VI.1995, B. Zheng”.

#### Diagnosis.

**Figures 2–9.**
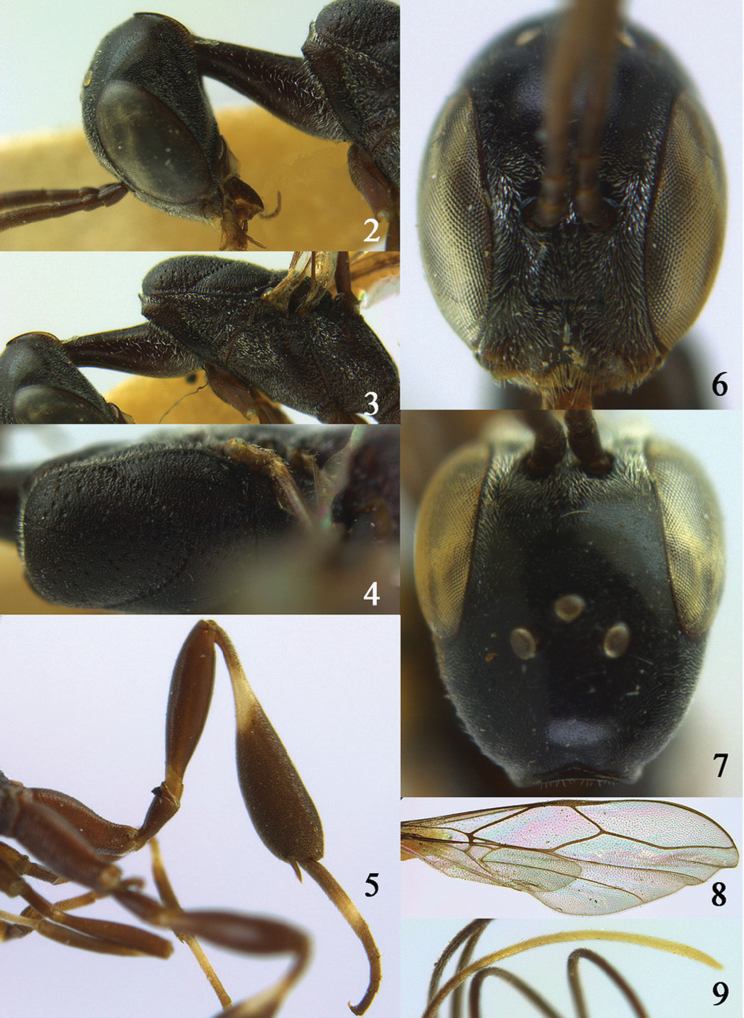
*Gasteruption amoyense* Pasteels, 1958, holotype, female. **2** head lateral **3** mesosoma lateral **4** mesoscutum dorsal **5** hind leg **6** head anterior **7** head dorsal **8** fore wing **9** apex of ovipositor sheath.

Head shiny and comparatively elongate and narrow (especially frons) in dorsal view, gradually narrowed behind eyes (Fig. 7); vertex slightly flattened medio-posteriorly, without a depression; occipital carina moderately to narrowly lamelliform medio-dorsally (Fig. 2); mesosoma 2.1–2.3 times as long as wide; propleuron slender and 1.1–1.2 times as long as mesoscutum in front of tegulae and resulting in a long neck (Fig. 3); antesternal carina medium-sized and lamelliform; mesoscutum matt, coriaceous and sparsely coarsely punctate, punctures medially well separated and without distinct transverse rugae except some rugae medially (Fig. 4); hind coxa and femur brown; apical half of hind tarsus of ♀ largely ivory or black; hind tibia distinctly inflated; ovipositor sheath 0.8–1.1 times as long as body and 3.8–5.3 times as long as hind tibia; mesoscutum mainly coriaceous or rugose, with coarsely punctate or reticulate-punctate (Fig. 4); mesosoma and metasoma black-brown or dark brown; pterostigma dark brown; pale apical part of ovipositor sheath (Fig. 9) about 1.7 times as long as hind basitarsus; body length 9.5–14.0 mm.

#### Description.

Female holotype of *Gasteruption amoyense*, body length 9.5 mm (of fore wing 4.2 mm).

*Head*. Head shiny, sparsely punctulate, comparatively elongate and narrow in dorsal view and gradually narrowed behind eyes (Fig. 7); vertex slightly flattened medio-posteriorly (Fig. 2), without a depression (Fig. 7); occipital carina moderately lamelliform medio-dorsally (Fig. 2); third and fourth antennal segments 1.5 times and twice as long as second segment; face comparatively narrow and rather short setose (Fig. 2); frons narrow, shiny and finely punctulate; OOL 1.6 times diameter of posterior ocellus; ventrally head not enlarged in anterior view (Fig. 6), malar space 0.3 times length of pedicellus.

*Mesosoma*. Length of mesosoma 2.1 times its height; propleuron 1.2 times as long as mesoscutum in front of tegulae, slender (Fig. 3); pronotum laterally coarsely crenulate medially and subposteriorly, coriaceous dorsally and ventrally largely smooth and shiny and with coarse punctures, densely pilose except ventrally; side of pronotum with a distinct tooth antero-ventrally; antesternal carina medium-sized, lamelliform and upcurved; middle and lateral lobe of mesoscutum mainly coarsely punctate and with shiny smooth interspaces (Fig. 4), medially with some transverse rugae and medio-posteriorly coarsely reticulate-punctate; scutellum largely smooth and with some fine punctures; mesosoma conspicuously white pilose laterally (Fig. 3); middle lobe slightly protuberant.

*Legs*. Length of hind femur, tibia and basitarsus 4.8, 4.4 and 5.1 times their width, respectively; hind tibia rather swollen and ventrally curved (Fig. 5); fore coxa distinctly removed from mesopleuron (Fig. 5); hind coxa transversely striate dorsally (except basally) and remainder coriaceous; hind basitarsus moderately slender (Fig. 5).

*Metasoma*. Ovipositor sheath as long as body, 1.3 times as long as metasoma, 3.1 times as long as hind tibia and tarsus combined and 5.3 times hind tibia; white apical part of ovipositor sheath 1.7 times as long as hind basitarsus; hypopygium v-shaped incised.

*Colour*. Black-brown or dark brown; mandible (including base), tegulae, trochantelli, apices and bases of femora narrowly yellow-brown; bases of fore and middle tibiae and a stripe anteriorly, middle basitarsus (except apically); subbasal ring of hind femur, hind basitarsus (except quarter) and apex of ovipositor sheath white; metasoma brown, but base and apex dark brown; remainder of fore and middle legs, and pterostigma dark brown; wing membrane subhyaline.

*Male*. Unknown.

*Variation*.Ovipositor sheath 0.8–1.1 times as long as body, 1.2–1.7 times as long as metasoma, 2.4–4.0 times as long as hind tibia and tarsus combined and 3.8–5.3 times as long as hind tibia; pale apical part of ovipositor sheath pale brown or white; length of fore wing 4.2–6.6 mm, of body 9.5–14.0 mm; head gradually narrowed behind eyes and weakly curved laterally; temple about 0.7 times as long as eye in dorsal view; third and fourth antennal segments 1.7 times and 2.2 times as long as second segment; fourth antennal segment 1.3 times as long as third segment, fifth antennal segment 1.2 times as long as third segment; minimum of malar space 0.1 times as long as second antennal segment; length of mesosoma 2.3 times as long as its height; propleuron slender; side of pronotum mainly coriaceous, ventrally slight rugulose-lacunose; mesoscutum mainly coriaceous, spaced punctate, middle lobe with coarsely punctures; scutellum mainly coriaceous; propodeum shiny and reticulate-rugose, longitudinal carina indistinct; hind coxa coriaceous to transversely rugose in dorsal view; length of hind femur, tibia and basitarsus 3.5, 4.3 and 5.4 times their width, respectively. Specimen from Zhejiang red-brown; mandible yellow-brown or dark brown; legs red-brown to orange brown, subbasal of hind tibia yellow, hind basitarsus without ivory patch.

#### Distribution.

China (Zhejiang, Fujian, Hunan, Hong Kong).

#### Biology. 

Unknown. Collected in May to June.

### Gasteruption angulatum sp. n.

urn:lsid:zoobank.org:act:47434E5A-CAF4-43A1-AB0D-870A57E124A1

http://species-id.net/wiki/Gasteruption_angulatum

Figs 10 23

#### Type material.

Holotype, ♀ (ZJUH), “[China:] Zhejiang, West Mt. Tianmu, Laodian–Xianrending, 1250–1547 m, 17–18.V.1988”. Paratypes: 1 ♀ (ZJUH), “[China:] Henan, Luoshan, Mt. Ling, 22.V.2000, Ping Cai”; 1 ♂ (SCAU), “[China:] Shaanxi, Ningshaan, Xunyangba, 1371m, 6.V.2011, Hua-yan Chen”; 2 ♀ (SCAU), “[China:] Hubei, Shennongjia, 15–19.V.2012, Kai Shi”; 2 ♀ + 1 ♂ (ZJUH), “[China:] Zhejiang, West Mt. Tianmu, VII.1981 & 2–4.VI.1990 & 16–18.V.1988”; 1 ♀ (RMNH), “[China:] Zhejiang, West Mt. Tianmu, Laodian–Xianrending, 1250–1547, 7.VI.1989, Jun-hua He”; 1 ♂ (ZJUH), “[China:] Zhejiang, West Mt. Tianmu, Xianrending, 30.VII.1998, Ming-shui Zhao”; 1 ♂ (SCAU), “[China:] Zhejiang, West Mt. Tianmu, Chantan temple, 350 m, 16.V.1988, Jin-jiang Fan”.

#### Diagnosis.

**Figures 10–18.**
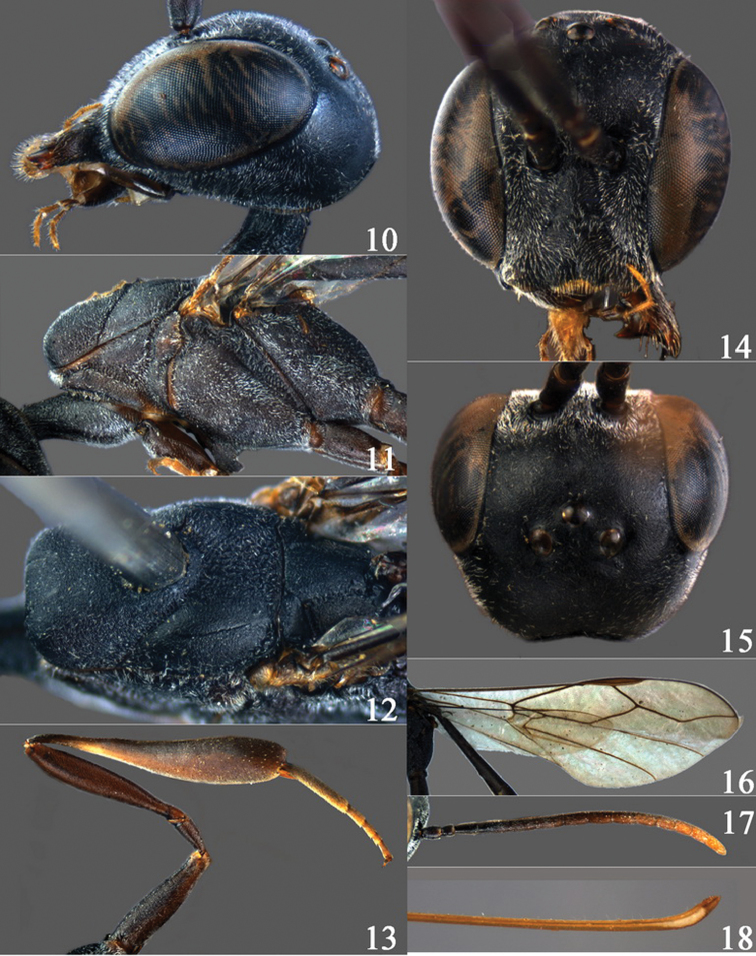
*Gasteruption angulatum* sp. n., holotype, female. **10** head lateral **11** mesosoma lateral **12** mesoscutum dorsal **13** hind leg **14** head anterior **15** head dorsal **16** fore wing **17** antenna **18** ovipositor and sheath.

Head evenly convex in front of occipital carina and without any depression medio-posteriorly; occipital carina narrow and non-lamelliform medio-dorsally (Fig. 10); length of mesosoma about twice its height; propleuron 0.7–0.8 times as long as mesoscutum in front of tegulae (Fig. 11); mesoscutum coriaceous and matt, medio-posteriorly slightly rugulose (Figs 12, 20); hind coxa extremely slender and coriaceous; ovipositor widened apically and more or less angularly upcurved apically in dead specimens (Fig. 18); ovipositor sheath about 0.3–0.5 times as long as body and 0.4–0.6 times as long as metasoma; pale apical part of ovipositor sheath 1.9–2.3 times as long as hind basitarsus.

#### Description.

Holotype, female, body length 14.5 mm.

*Head*. Head directly narrowed behind eyes and distinct curved laterally (Fig. 15), emarginated distinctly medio-posteriorly; temple 0.5–0.6 times as long as eye in dorsal view (Fig. 15); vertex and frons matt and coriaceous; vertex moderately convex posteriorly (Fig. 10) and without any depression medio-posteriorly (Fig. 15); occipital carina narrow and non-lamelliform medio-dorsally (Fig. 10); third antennal segment 1.5 times as long as second segment; fourth antennal segment 1.3 times as long as third segment; fifth antennal segment 1.2 times as long as third segment (Fig. 17); eye glabrous; OOL 1.8 times as long as diameter of posterior ocellus; minimum width of malar space 0.2 times as long as second antennal segment; clypeus without depression (Fig. 14; cf. ♂ Fig. 22).

*Mesosoma*. Length of mesosoma twice its height; propleuron robust (Fig. 11), 0.7–0.8 times as long as mesoscutum in front of tegulae; side of pronotum mainly coriaceous, but dorsally a few rugulose, with a distinct antero-lateral tooth; mesoscutum coriaceous and matt (Fig. 12); medio-posteriorly slightly rugulose; scutellum coriaceous and matt; mesopleuron coriaceous; propodeum reticulate-rugose, medio-longitudinal carina distinct.

*Wings*. Fore wing: first discal cell parallel-sided and with outer posterior corner rounded (Fig. 16).

*Legs*. Hind coxa matt, extremely slender and coriaceous (Fig. 13); length of hind femur, tibia and basitarsus 4.9, 5.5 and 5.5 times their width, respectively; middle tarsus 1.2 times as long as middle tibia.

*Metasoma*. Apical of ovipositor width and more or less angularly upcurved (Fig. 18); ovipositor sheath 0.5 times as long as body, 0.6 times as long as metasoma, twice as long as hind tibia and 1.2 times combined hind tarsus and tibia; its ivory part 2.1 times as long as hind basitarsus and 0.3 times as long as ovipositor sheath; hypopygium shallow v-shaped apically.

*Colour*. Black-brown; mandible dark brown; antenna dark brown, gradually shallow; legs dark brown, fore and middle tarsi brown; metasoma dark brown.

**Figures 19–23.**
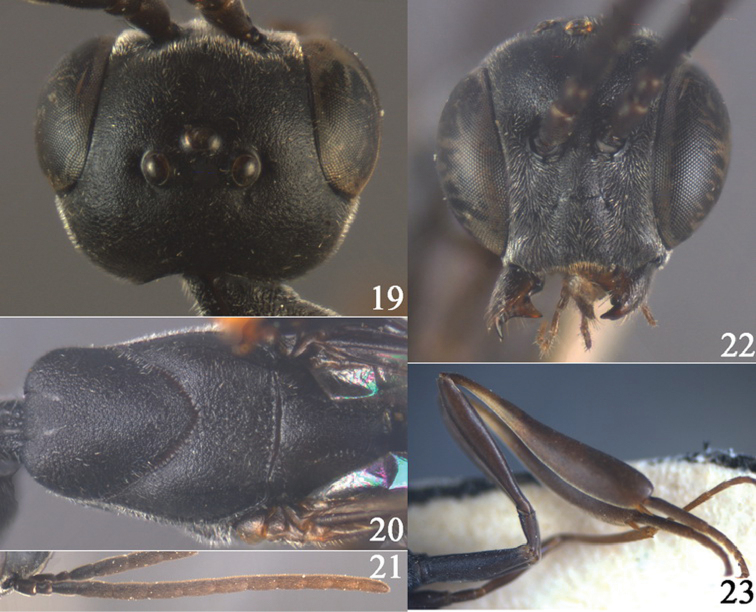
*Gasteruption angulatum* sp. n., paratype, male. **19** head dorsal **20** mesoscutum dorsal **21** antenna **22** head anterior **23** hind legs.

*Male* (described after a male from China: Zhejiang). Body length 11.5 mm; head strongly curved laterally; temple 0.6 times as long as eye in dorsal view (Fig. 19); third antennal segment 1.5 times as long as second segment, fourth 1.4 times as long as third segment, fifth antennal segment as long as fourth segment and 1.4 times as long as third segment (Fig. 21); OOL 1.6 times as long as diameter of posterior ocellus; minimum width of malar space 0.4 times as long as second antennal segment; length of mesosoma 1.9 times as long as its height; propleuron 0.8 times as long as mesoscutum in front of tegulae; length of hind femur, tibia and basitarsus 5.6, 5.5 and 5.1 times their width, respectively (Fig. 23).

*Variation*. Female: body length 11.5–13.0 mm, ovipositor sheath 0.3–0.5 times as long as body, 0.4–0.6 times as long as metasoma, 1.6–2.0 times as long as hind tibia and 1.1–1.2 times combined hind tarsus and tibia; its ivory part 1.9–2.3 times as long as hind basitarsus and 0.3 times as long as ovipositor sheath; third antennal segment 1.5–1.6 times as long as second segment; fourth antennal segment 1.2–1.3 times as long as third segment; fifth antennal segment 1.1–1.2 times as long as third segment. Male: very similar to female in structure and color, but differs in size; body length 11.3–12.0 mm.

#### Distribution.

China (Shaanxi, Henan, Hubei, Zhejiang).

#### Biology.

Unknown. Collected in May to July.

#### Etymology.

Named after the more or less angulate apex of ovipositor in dead specimens; “angulus” is corner or bend in Latin.

### Gasteruption assectator

(Linnaeus, 1758)

http://species-id.net/wiki/Gasteruption_assectator

Figs 24 37

Ichneumon assectator Linnaeus, 1758: 566, 1761: 407, 1767: 937; Scopoli, 1763: 287; Fabricius 1775: 340, 1781: 435, 1787: 268; Gmelin, 1789: 2696; Villers, 1789: 174; Rossi, 1790: 90; Christ, 1791: 375; Petagna, 1792: 365; Cederhjelm, 1798: 163; Schrank, 1802: 263; Hentschius, 1804: 112; Illiger, 1807: 74; Roman 1932: 2; Hedqvist 1973: 182; Fitton 1978: 376.
Foenus assectator ; Fabricius 1798: 240; Walckenaer, 1802: 75; Latreille 1805: 195; Dahlbom, 1831: 77; Curtis, 1832: 423; Nees, 1834: 308; Stephens, 1835: 121; Labram, 1838: 1; Zetterstedt, 1840: 408; Westwood 1843: 255; Taschenberg, 1866: 93; Tournier, 1877: ix (as *affectator*); Thomson 1883: 849.
Faenus affectator ; Abeille de Perrin 1879: 265, 266, 277.
Gasteruption assectator ; Schletterer 1885: 276, 316, 1889: 384, 393, 395, 397; Dalla Torre 1902: 1063; Szépligeti 1903: 370 (as *affectator*); Kieffer 1912: 256 (id.); Lindemans, 1921: 298 (id.); Schmiedeknecht, 1930: 380, 383 (as *affectator*); Roman 1932: 2; Hedicke 1939: 5 (id.); Ferrière, 1946: 235, 238, 240 (id.); Leclercq, 1948: 75; Hellén, 1950: 4; Townes, 1950: 123-128; Šedivý 1958: 36, 37; Györfi and Bajári, 1962: 48, 51; Schmidt, 1969: 293; Hedqvist 1973: 181; Fitton 1978: 376; Dolfuss, 1982: 22; Oehlke 1984: 169, 171, 175; Ortega and Baez, 1985: 509, 515; Madl, 1987d: 401, 1987b: 21, 1988: 37, 1989a: 159, 1989b: 41, 1990a: 127, 1990b: 480; Kozlov 1988: 245, 247; Kofler and Madl 1990: 320; Narolsky and Shcherbal 1991: 23, 24; Wall 1994: 150; Scaramozzino, 1995: 3; Peeters, 1996: 134; Smith 1996: 492; Pagliano and Scaramozzino 2000: 11, 19; Saure, 2001: 29; Turrisi 2004: 84; Yildirim et al. 2004: 1350; van der Smissen, 2010: 372.
Ichneumon annularis Geoffroy, 1785: 398; Hedicke 1939: 7; Wall 1994: 148. Synonymized by with *Gasteruption assectator* (Linnaeus) by Olivier, 1792.
Foenus borealis Thomson, 1883: 849; Hedicke 1939: 7; Hedqvist 1973: 181, 182 (invalid lectotype designation); Wall 1994: 148. Synonymized with *Gasteruption assectator* (Linnaeus) by Schletterer 1889.
Gasteruption boreale ; Schletterer 1885: 303.
Foenus fumipennis Thomson, 1883: 848; Hedicke 1939: 7; Hedqvist 1973: 181, 182 (lectotype designation); Wall 1994: 148. Synonymized with *Gasteruption assectator* (Linnaeus) by Schletterer 1885.
Foenus nigritarsis Thomson, 1883: 849; Schletterer 1889: 398; Hedicke 1939: 7; Hedqvist 1973: 181, 182 lectotype designation); Wall 1994: 149. Synonymized with *Gasteruption assectator* (Linnaeus) by Schletterer 1889.
Gasteruption nigritarse ; Schletterer 1885: 310.
Gasteruption brevicauda Kieffer, 1904a: 648, 1904b: 18, 1912: 259; Hedicke 1939: 8; Madl 1987d: 401; Wall 1994: 148. Synonymized with *Gasteruption assectator* (Linnaeus) by Madl 1987a.
Gasteruption abeillei Kieffer, 1912: 228, 231, 251; Hedicke 1939: 5; Ferrière, 1946: 235, 240; Leclercq, 1948: 75; Wall 1994: 148. Synonymized with *Gasteruption assectator* (Linnaeus) by Madl 1989a.
Trichofoenus breviterebrae Watanabe, 1934: 285; Hedicke 1939: 45. Synonymized by Pagliano and Scaramozzino 2000: 11, 19.
Gasteruption rugulosum ; Malyshev 1965: 245.
Gasteruption margotae Madl, 1987a: 225, 1990b: 480; Wall 1994: 149. Synonymized by with *Gasteruption assectator* (Linnaeus) by Madl 1990b.
Gasteruption affectator auct.

#### Type material.

Holotype of *Gasteruption breviterebrae*, ♀ (Sapporo), “[Russia:] Saghalien [= Sakhalin Oblast], K. Tamanuki/ Konuma, 23.v.1931”, “Holotype *Trichofoenus breviterebrae* Watanabe, 1934, det. Konishi”. Paratypes: only 1 ♂ (Sapporo) examined, “[Russia:] Saghalien, K. Tamanuki/Nagahama, 28.vii.1927”, “Paratype (Allotype) *Trichofoenus breviterebrae* Watanabe, 1934”.

#### Additional material.

Japan (Hokkaido: Ashoro, Tokachi; Antaroma-Aizankeu. Honshu: Koike, Hakusan; Hirosaki, Aomori); China (Heilongjiang, Harbin (ZJUH); Jilin, Mt. Changbai, Daobai River, 740 m; Jilin, Daxinggou (ZJUH); Inner Mongolia, Bayannaoer (ZJUH); Ningxia, Jingyuan, Mt. Liupan (ZJUH); Beijing, Gongzhufen (ZJUH); Hebei, Mt. Xiaowutai (ZJUH); Henan, Xinxiang (ZJUH); Qinghai, Nangqian, 4288 m, N31°58.399', E96°30.757' and Mt. Qilian, Menyuan, 3300 m; Xinjiang, Hami; Xinjiang, Qihe, Buergen River, N46°09.006', E101°18.775', 1148 m; Xinjiang, Bole, Xiaerxili, N45°13.289', E82°04.533', 1863 m; Xinjiang, Yili, Gongnaisi, N43°10.948', E84°19.763', 2425 m; Shanxi, Fengxian, Mt. Jiantai, 1700 m; Hubei, Shennongjia Nature Reserve, Xiaolongtan, N31°15', E109°56', 1800 m; Hunan, Zhuzhou(CSCS); Sichuan, Wolong Nature Reserve; Far East Russia (Sakhalin).

#### Diagnosis.

**Figures 24–32.**
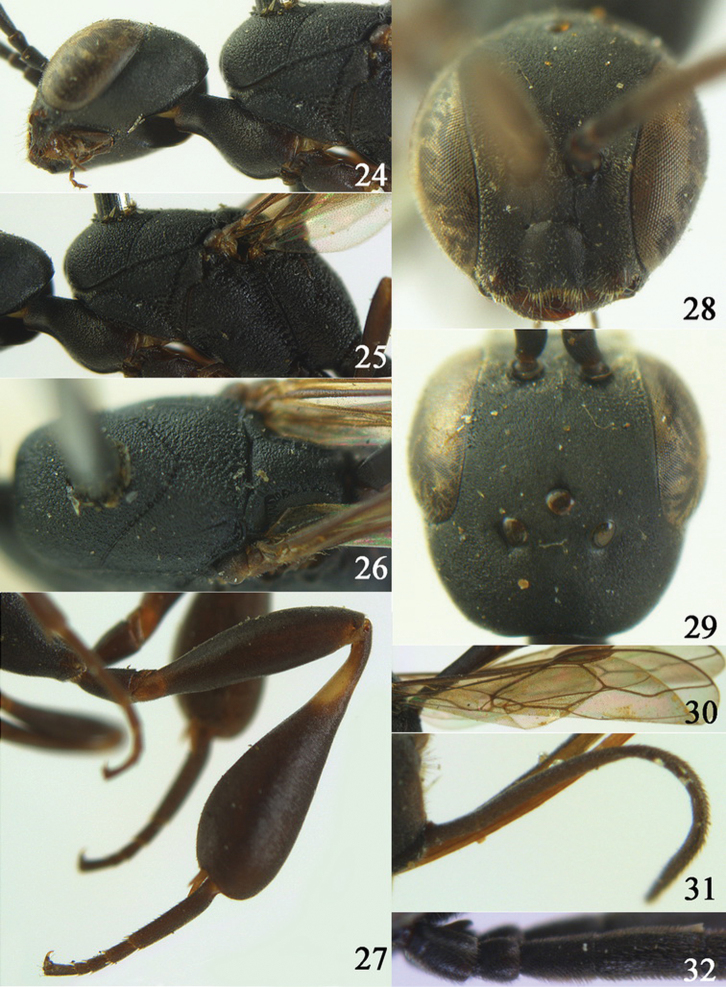
*Gasteruption assectator* (Linnaeus, 1758), holotype, female. **24** head lateral **25** mesosoma lateral **26** mesoscutum dorsal **27** hind leg **28** head anterior **29** head dorsal **30** fore wing **31** apex of ovipositor sheath **32** the first to fourth antennal segments.

**Figures 33–37.**
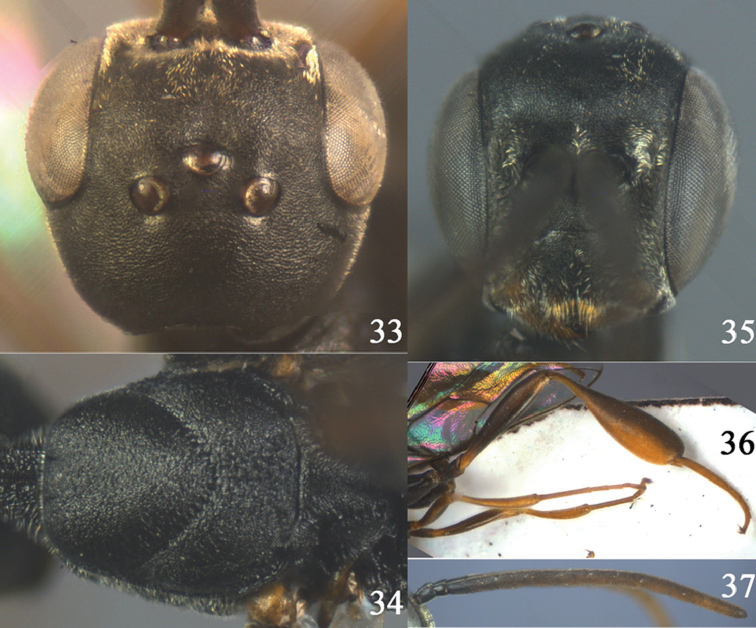
*Gasteruption assectator* (Linnaeus, 1758), male, Inner Mongolia. **33** head dorsal **34**  mesoscutum dorsal **35** head anterior **36** hind leg **37** antenna.

Apex of ovipositor sheath black or slightly brown (Fig. 31), if rather pale apically then pale part shorter than 0.3 times hind basitarsus; ovipositor sheath 0.8–1.3 times as long as hind tibia and 0.4–0.8 times as long as hind tibia and tarsus combined; occipital carina obsolescent medio-dorsally (Figs 29, 33) and rather protruding ventro-posteriorly (Fig. 24); antesternal carina narrow; head, laterally mesosoma and scape black; head in anterior view slightly protruding below lower level of eyes by less than basal width of mandible and mandibular condylus near lower level of eyes (Figs 28, 35); in lateral viewcondylarincision of malar space close to eye (Fig. 24); clypeus with small depression or depression obsolescent; eyes shortly setose; fourth and fifth antennal segment 1.1–1.3 (♀)–1.4 (♂) and 0.9–1.1 (♀)–1.4 (♂) times as long as third segment, respectively (Figs 32, 37); apical antennal segment at most 1.2 times as long as third antennal segment and its colour similar to colour of medial segments; antenna of female may be partly yellow-brown; mesoscutum and head similarly coriaceous, at most mesoscutum superficially rugulose (Figs 26, 34); propleuron robust and about 0.8 times as long as mesoscutum in front of tegulae (Fig. 25); hind coxa often transversely rugose dorsally, but sometimes mainly coriaceous; hind tibia robust, with a distinct subbasal ivory ring and swollen, resulting in a distinctly convex ventral border (Fig. 27, 36); hind basitarsus comparatively long (Fig. 27, 36); hind tibial spurs yellow-brown or brown; hind tarsus brown, dark brown or black; incision of hypopygium shallow.

#### Description.

Holotype of *Gasteruption breviterebrae*, female, body length 8.9 mm.

*Head*. Vertex and frons matt and very densely finely coriaceous, moderately convex (Fig. 24) and without a depression medio-posteriorly (Fig. 29); head gradually narrowed behind eyes; temple 0.6 times as long as eye in dorsal view (Fig. 29); fourth antennal segment 1.2 times as long as third segment and 0.7 times as long as second and third segments combined, fifth antennal segment 1.1 times as long as third segment (Fig. 32), third antennal segment long and 1.5 times as long as second segment; occipital carina narrow and non-lamelliform medio-dorsally (Fig. 29); ocelli comparatively small, OOL 1.4 times as long as diameter of posterior ocellus; face rather wide (Fig. 28); minimum width of malar space 0.5 times as long as second antennal segment (Fig. 24); clypeus with small triangular depression and slightly emarginate (Fig. 28); eye setose.

*Mesosoma*. Length of mesosoma 1.7 times its height; pronotal side moderately high and ventrally coriaceous, without distinct antero-lateral tooth; mesoscutum slightly protruding anteriorly; propleuron robust, 0.8 times as long as mesoscutum in front of tegulae (Fig. 25); antesternal carina narrow and hardly lamelliform; mesoscutum densely coriaceous and rather matt, posteriorly with some rugae (Fig. 26); scutellum coriaceous.

*Wings*. First discal cell parallel-sided and with posterior corners rounded (Fig. 30).

*Legs*. Hind coxa rather matt, robust, coriaceous; length of hind femur, tibia and basitarsus 3.9, 3.2 and 5.0 times their width, respectively; middle tarsus normal (Fig. 27), as long as middle tibia; middle femur subparallel-sided and slenderer than fore femur.

*Metasoma*. Ovipositor sheath 0.2 times as long as body, 0.3 times as long as metasoma and 0.9 times as long as hind tibia; sheath apically dark brown; hypopygium shallow v-shaped apically.

*Colour*. Black or black-brown; second–fourth metasomal tergites apically and antenna (except for four black basal segments) more or less brown, tegulae and legs largely dark brown, but hind tibial spurs pale brown and hind tibia with large ventral subbasal patch ivory; pterostigma brown.

*Male*. Paratype. Head behind eyes roundly narrowed in dorsal view (Fig. 33); occipital carina distinctly pigmented, narrow and non-lamelliform medio-dorsally (Fig. 33); face wide (Fig. 35); third antennal segment about 1.7 times as long as second segment (Fig. 37); fourth segment 1.4 times as long as third segment, as fifth segment, and 0.8 times as long as second and third segments combined; vertex coriaceous, matt; eye glabrous; frons rather convex and anterior ocellus above upper level of frons (Fig. 35); propleuron robust and 0.8 times as long as mesoscutum in front of tegulae; antesternal carina non-lamelliform and narrow; mesoscutum densely coriaceous, mixed with fine transverse rugulosity and posteriorly distinctly rugose (Fig. 34); hind coxa slender in dorsal view; hind basitarsus transversely rugose dorsally; hind leg coloured as in female but hind tarsus largely brown (Fig. 36); apical half of first metasomal tergite and second tergite black; paramere black apically; body length 9.7 mm. Very similar to female, but slightly more coarsely sculptured.

*Variation*. Female: body length 8.7–14.0 mm, ovipositor sheath 0.8–1.3 times as long as hind tibia. Temple 0.6–0.7 times as long as eye in dorsal view; occipital area towards inside concave more or less; third antennal segment 1.5–1.7 times as long as second segment, fourth antennal segment 1.1–1.3 times as long as third segment, fifth antennal segment 0.9–1.0 times as long as third segment; OOL 1.3–1.4 times as long as diameter of posterior ocellus; minimum width of malar space 0.3–0.5 times as long as second antennal segment; length of hind femur, tibia and basitarsus 4.1–4.3, 3.4–3.6 and 4.9–5.4 times their width, respectively; ovipositor sheath black, 1.0–1.5 times as long as hind tibia. Male: body length 8.0–12.0 mm, very similar to female. Third antennal segment 1.1–1.3 times as long as second segment, fourth antennal segment 1.3–1.4 times as long as third segment, fifth antennal segment as long as fourth segment.

#### Distribution.

China (Heilongjiang, Jilin, Inner Mongolia, Xinjiang, Beijing, Hebei, Henan, Shanxi, Ningxia, Qinghai, Hubei, Hunan, Sichuan, Tibet); Russia (Sakhalin Oblast); Japan.

#### Biology

. Unknown. Collected in May–August.

#### Notes.

There are no distinct morphological differences between the West and East Palaearctic populations.

### Gasteruption assectoides sp. n.

urn:lsid:zoobank.org:act:354D4503-A761-4CD6-BB0F-96AE579C6BA1

http://species-id.net/wiki/Gasteruption_assectoides

Figs 38–46

#### Type material.

Holotype, ♀ (CSCS), “[China:] Hubei, Shennongjia Nature Reserve, Banbiyan, 2600 m, 26.VI.2002, Yi-hai Zhong”.

#### Diagnosis.

**Figures 38–46.**
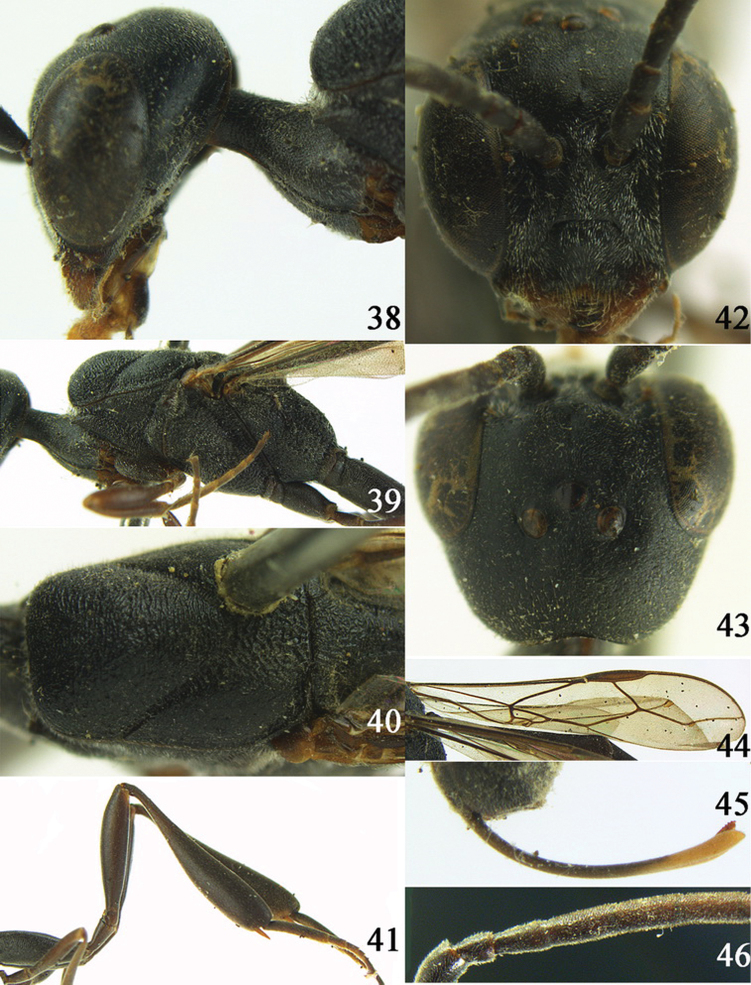
*Gasteruption assectoides* sp. n., holotype, female. **38** head lateral **39** mesosoma lateral **40** mesoscutum dorsal **41** hind legs **42** head anterior **43** head dorsal **44** fore wing **45** ovipositor sheath **46** the first to sixth antennal segments.

Apex of ovipositor sheath ivory, ivory part 0.8 times as long as hind basitarsus; ovipositor sheath comparatively wide (Fig. 45); ovipositor sheath 0.9 times as long as hind tibia and 0.6 times as long as hind tibia and tarsus combined; occipital carina obsolescent medio-dorsally (Fig. 43) and rather protruding ventro-posteriorly (Fig. 38); antesternal carina narrow; head, laterally mesosoma and scape black; head in anterior view slightly protruding below lower level of eyes by less than basal width of mandible and mandibular condylus near lower level of eyes (Fig. 42); in lateral viewcondylarincision of malar space close to eye (Fig. 38); clypeus with shallow triangular depression at ventral 0.8 (Fig. 42); eyes inconspicuously setose; fourth and fifth antennal segment 1.4 and 1.2 (♀) times as long as third segment, respectively (Fig. 46); apical antennal segment 1.2 times as long as third antennal segment and paler than medial segments; antenna of female yellow-brown apically; sculpture of mesoscutum and head dissimilar, matt (Fig. 40); head dorsally densely coriaceous, but mesoscutum densely rugulose and medio-posteriorly reticulate-rugose; propleuron robust and 0.8 times as long as mesoscutum in front of tegulae (Fig. 39); hind coxa finely transversely striate and rather slender; hind tibia narrow and elongate, without subbasal ivory patch and ventral border slightly convex (Fig. 41); hind basitarsus comparatively long (Fig. 41); hind tibial spurs yellow-brown; hind tarsus dark brown; apical sixth of hypopygium incised and hypopygium pale apically.

#### Description. 

Holotype, female, body length 17.6 mm, of fore wing 9.0 mm.

*Head*. Vertex and frons matt and very finely densely coriaceous, moderately convex (Fig. 38) and without a depression medio-posteriorly (Fig. 43); head gradually narrowed behind eyes and U-shaped excised medio-posteriorly (Fig. 43; more distinctly so if head in plane of vertex); temple 0.7 times as long as eye in dorsal view (Fig. 43); fourth antennal segment 1.4 times as long as third segment and 0.8 times as long as second and third segments combined, fifth antennal segment 1.2 times as long as third segment (Fig. 46), third antennal segment 1.4 times as long as second segment; occipital carina narrow and non-lamelliform medio-dorsally; ocelli comparatively small, OOL 1.8 times as long as diameter of posterior ocellus; face wide (Fig. 42); minimum width of malar space 0.3 times as long as second antennal segment (Fig. 38); clypeus with shallow triangular depression up to dorsal fifth and slightly emarginate (Fig. 42); eye inconspicuously setose.

*Mesosoma*. Length of mesosoma twice its height; pronotal side moderately high and ventrally largely rugulose, without distinct antero-lateral tooth, but angularly protruding (Fig. 39); mesoscutum slightly protruding anteriorly; propleuron robust and 0.8 times as long as mesoscutum in front of tegulae; antesternal carina narrow and hardly lamelliform; mesoscutum densely rugulose, medio-posteriorly reticulate-rugose and matt; scutellum irregularly rugose (Fig. 40).

*Wings*. First discal cell elongate triangular and with vein 3-CU1 near apical third (Fig. 44).

*Legs*. Hind coxa finely transversely striate, rather slender and with satin sheen; length of hind femur, tibia and basitarsus 4.5, 5.6 and 6.0 times their width, respectively; middle tarsus elongate (Fig. 41), 1.2 times as long as middle tibia; middle femur subparallel-sided and much slenderer than fore femur.

*Metasoma*. Ovipositor sheath 0.2 times as long as body, 0.3 times as long as metasoma and 0.9 times as long as hind tibia; ivory apical part of sheath 0.8 times as long as hind basitarsus; hypopygium shallow v-shaped apically.

*Colour*. Black or black-brown; mandible yellow-brown; antenna apically yellow-brown, tegulae and legs more or less dark brown, but hind tibial spurs yellow-brown; pterostigma dark brown; wing membrane subhyaline.

*Male*. Unknown.

#### Distribution.

China (Hubei).

#### Biology.

Unknown. Collected in June in montane zone (2600 m).

#### Etymology.

From the specific name “*assectator*” and “oides” (Latin for “resembling”), because of its superficial similarity to *Gasteruption assectator*.

### Gasteruption bimaculatum

Pasteels, 1958

http://species-id.net/wiki/Gasteruption_bimaculatum

Figs 47 62

Gasteruption bimaculatum Pasteels, 1958: 191–192, fig. 16 (only holotype ♂).
Gasteruption obscuripenne Pasteels, 1958: 189–190, fig. 14 (Chinese paratypes).

#### Type material.

Holotype, ♂ (NRS), “N.E. Burma, Sadon, 17.III.1934, R. Malaise”, “*Gasteruption bimaculatum* n. sp., J. Pasteels det. 1954”, “Holotype”, “NHRS-HEVA 000001977”. Paratypes from Sumba are excluded. Two paratype females of *Gasteruption obscuripenne* from Hainan (USNM: Chue Mo Ling (= Zhumuling), NE of Nodoa (= Nada), 21.VIII.1929 and NW of Nodoa (= Nada), 27.VIII.1929).

#### Additional material.

1 ♀ (ZJUH), “[China:] Henan, Xinxiang, 28.IV.1980, Cheng-zhong Xu”; 1 ♂ + 1 ♀ (CSCS) “[China:] Fujian, Mt. Wuyi, 7.VII.2007, 1000 m, Ji-gang Jiang & Xiao Wei”; 1 ♀ (ZJUH), “[China:] Fujian, Jiangle, Mt. Longqi, 1.VII.1991, Chang-ming Liu”; 1 ♀ (ZJUH), “[China:] Fujian, Fuzhou, 10.V.1993, Chang-ming Liu”; 2 ♀ (ZJUH, RMNH), “[China:] Fujian, Fuzhou, Jinshan, 3–9.V.1990, Xiu-fu Zhao”; 1 ♀ (ZJUH), “[China:] Fujian, Fuzhou, Meihua, 20.IV.1960, Bi-ying Lin”; 1 ♀ (ZJUH), “[China:] Hainan, Wanning, Mt. Jianfengling, 13.V.2008, Li Ma”; 1 ♀ (SCAU), “[China:] Hainan, Mt. Yinggeling, 17.XI.2008, Man-man Wang”; 1 ♂ (ZJUH), “[China:] Guangxi, Longzhou, Wudesanlian, 350–400 m, 13.VI.2000, Jun Chen”; 1 ♀ (CSCS) “[China:] Tibet, Motuo, 17.VI.2009, 2750 m, Mei-cai Wei & Geng-yun Niu”; 1 ♀ (CSCS) “[China:] Yunnan, Qujing, Malong, Malianhe, Zhonghe, 19.VII., He-sheng Wang”; 1 ♀ (SCAU), “[China:] Yunnan, Jinggu, Weiyuan, 4.X.2004, Jing-xian Liu” ; 1 ♀ (ZJUH), “[China:] Yunnan, Hekou, Nanxi, 13. VII.2012, Xiao-ling Ji”.

#### Diagnosis.

**Figures 47–54.**
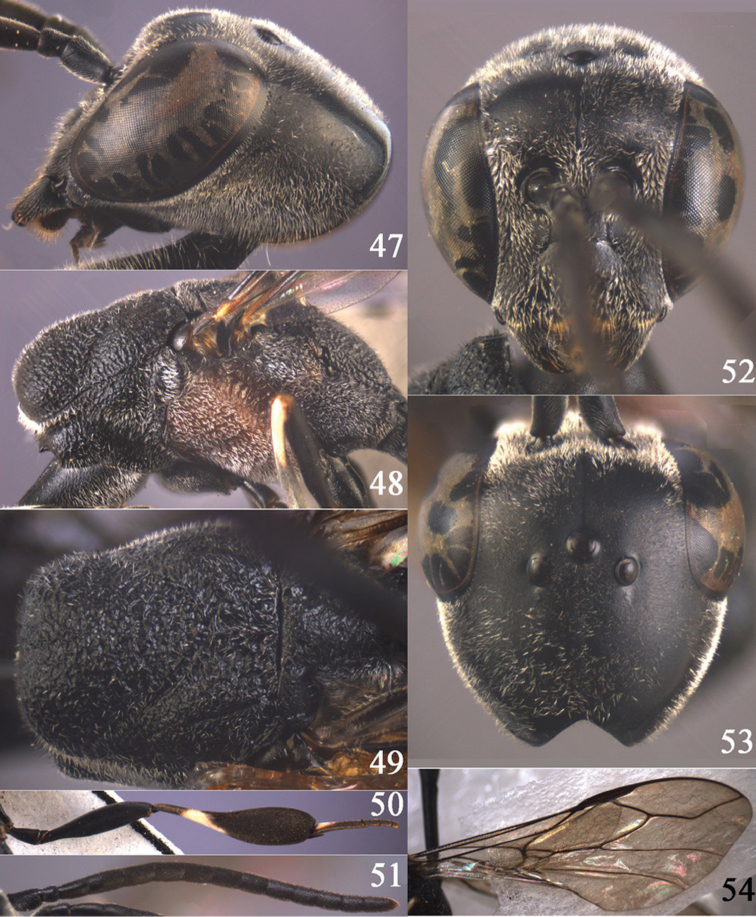
*Gasteruption bimaculatum* Pasteels, 1958, female, Hainan. **47** head lateral **48** mesosoma lateral **49** mesoscutum dorsal **50** hind leg **51** antenna **52** head anterior **53** head dorsal **54** fore wing.

**Figures 55–62.**
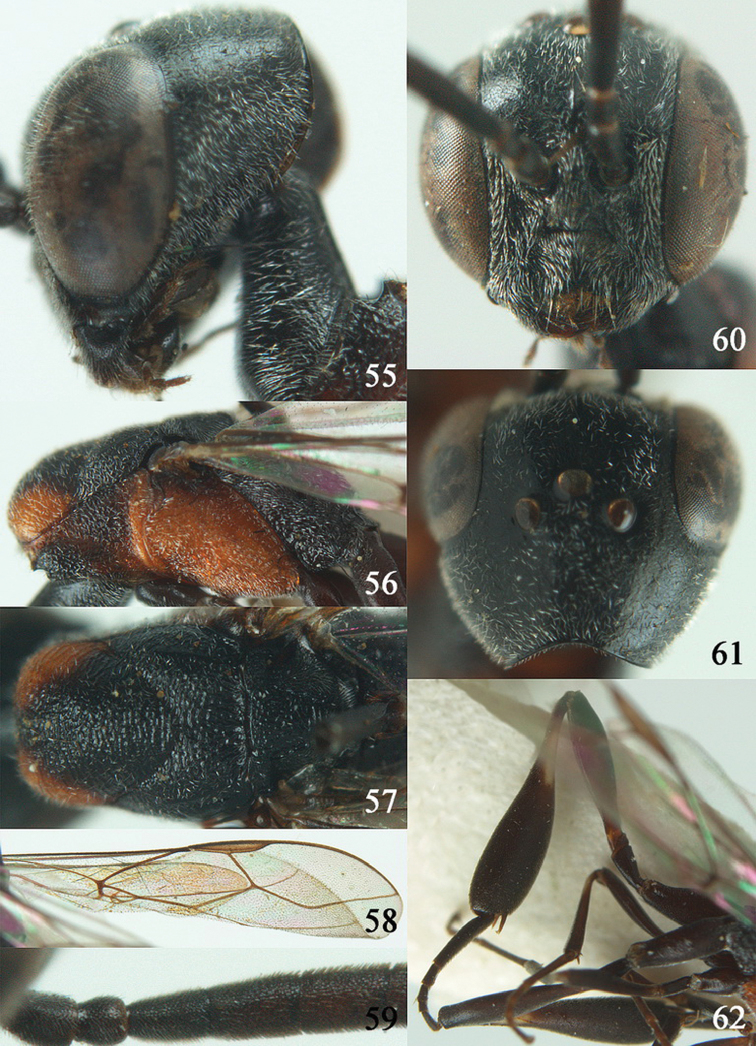
*Gasteruption bimaculatum* Pasteels, 1958, holotype, male. **55** head lateral **56** mesosoma lateral **57** mesoscutum dorsal **58** fore wing **59** the first to fifth antennal segments **60** head anterior **61** head dorsal **62** hind legs.

Head without a depression in front of occipital carina (Figs 53, 61); antesternal carina non-lamelliform and narrow (Fig. 48); propleuron 0.8 times as long as mesoscutum in front of tegulae and rather robust (Fig. 55); occipital carina obsolescent medio-dorsally (Figs 47, 55); head gradually narrowed behind eyes in dorsal view (Figs 53, 61); temple 0.7 times as long as eye in dorsal view; OOL (♂) 1.3 times as long as diameter of posterior ocellus; third antennal segment of Chinese female specimen 1.5 times as long as second segment, fourth antennal segment 1.1 times as long as third segment (Fig. 51), of male fourth segment 1.1 times as long as third segment and 0.7 times as long as second and third segments combined (Fig. 59); fifth antennal segment of female as long as third segment (Fig. 51), of male 1.1 times as long as third segment (Fig. 59) and penultimate segments unknown; third antennal segment of male normal, 1.6 times as long as second segment (Fig. 59); vertex largely smooth and shiny; malar space short (Figs 47, 55); antero-lateral teeth of pronotum medium-sized; mesoscutum robust (Fig. 55) and rather finely transversely rugose (medially somewhat reticulate), without separate punctures; marginal cell of fore wing elongate (Figs 54, 58); hind coxa rather slender and weakly irregularly transversely rugose; hind coxa dark brown; pronotal side mainly dark brown but medially and posteriorly brown or orange brown; hind basitarsus of male black-brown (but basally somewhat brown) and hind tibia bicoloured (dorsally dark brown, ventrally with large ivory subbasal patch) and comparatively wide (Fig. 62); female tibia distinctly clavate, apically swollen, subbasally with large ivory patch; tarsus entirely black; basitarsus slender and sometimes with ivory basal patch (Fig. 50); apical half of paramere black; ovipositor sheath black apically, sheath of Chinese specimens 0.6–0.9 times as long as body, 1.0–1.4 times as long as metasoma and 3.0–4.1 times as long as hind tibia and 1.9–2.4 times as long as hind tibia and tarsus combined; mesoscutum coarsely or finely transversely rugose or rugulose (Figs 49, 57).

#### Description.

Holotype, male (according to original description, metasoma missing), length of head + mesosoma 4.2 mm.

*Head*. Vertex shiny and largely smooth, densely setose, flat in lateral view (Fig. 55) and without depression medio-posteriorly (Fig. 61); head gradually narrowed behind eyes; temple 0.7 times as long as eye in dorsal view (Fig. 61); fourth antennal segment 1.1 times as long as third segment and 0.7 times longer than second and third segments combined, fifth antennal segment 1.1 times as long as third segment (Fig. 59), third antennal segment 1.6 times as long as second segment; occipital carina non-lamelliform and obsolescent medio-dorsally (Fig. 55); eyes short setose; ocelli large, OOL 1.3 times as long as diameter of posterior ocellus and POL 2.2 times; face narrow (Fig. 60); malar space 0.3 times as long as second antennal segment (= pedicellus).

*Mesosoma*. Length of mesosoma 1.7 times its height; pronotal side normal and mainly rather weakly reticulate-rugose, but postero-ventrally partly smooth; antero-lateral teeth of pronotum medium-sized; mesoscutum not protruding anteriorly (Fig. 57); propleuron 0.8 times as long as mesoscutum in front of tegulae; antesternal carina narrow and hardly lamelliform; middle and lateral lobes of mesoscutum shiny and moderately transversely rugose, medially somewhat reticulate-rugose, without separate punctures; scutellum weakly reticulate-rugose; propodeum coarsely reticulate-rugose, with a median smooth and somewhat raised area; nearly entire mesopleuron coarsely reticulate-rugose.

*Wings*. First discal cell elongate triangular, parallel-sided and no distinct distal posterior corner and its base subvertical (Fig. 58).

*Legs*. Hind coxa with satin sheen, weakly transversely rugose or rugulose dorsally; length of hind femur, tibia and basitarsus 4.5, 4.2 and 5.6 times their width, respectively; hind tibia comparatively wide (Fig. 62).

*Metasoma*. Missing in holotype; apical half of parameres of other specimens black.

*Colour*. Black or black-brown (including mandible); pronotal side mainly dark brown but medially and posteriorly brown or orange brown; mesoscutum antero-laterally and mesopleuron orange brown; base of fore and middle tibiae and ventrally hind tibia with large subbasal patch ivory; hind tibial spurs and pterostigma brown; wings subhyaline.

*Female* (described after a female from Fujian (Fuzhou, Jinshan). Body length 16 mm.

*Head*. Head deeply V-shaped emarginate medio-posteriorly, gradually narrowed behind eyes and weakly curved laterally (Fig. 53); temple 0.9 times as long as eye in dorsal view; vertex and frons with satin sheen, densely pubescence and coriaceous; vertex moderately flat posteriorly, occipital area towards inside concave, occipital carina non-lamelliform and obsolescent medio-dorsally (Fig. 47); eye setose; third antennal segment 1.5 times as long as second segment, fourth antennal segment 1.1 times as long as third segment, fifth antennal segment as long as third segment (Fig. 51); OOL twice as long as diameter of posterior ocellus; minimum width of malar space 0.2 times as long as second antennal segment; clypeus with an indistinct triangular depression (Fig. 52).

*Mesosoma*. Length of mesosoma 1.9 times as long as its height; propleuron with satin sheen, comparatively smooth except for just finely wrinkled and punctulate, as long as mesoscutum in front of tegulae; side of pronotum mainly coarsely rugose, dorso-laterally coriaceous, with a distinct antero-lateral tooth; mesoscutum with coarsely rugose, medio-posteriorly very similar but more coarsely sculptured than formerly (Fig. 49); scutellum weakly rugose; propodeum with densely transversely rugose, medio-longitudinal carina distinct.

*Wings*. Fore wing: first discal cell parallel-sided and with outer posterior corner rounded (Fig. 54).

*Legs*. Hind coxa with satin sheen, slender and dorsally transversely rugose; length of hind femur, tibia and basitarsus 4.0, 4.4 and 5.0 times as long as their width, respectively (Fig. 50); middle tarsus 1.1 times as long as middle tibia; hind tibia 3.5 mm.

*Metasoma*. Ovipositor sheath 0.7 times as long as body, as long as metasoma, 1.9 times as long as hind tibia and tarsus combined, and 3.1 times as long as hind tibia; hypopygium deep slit-shaped incised apically.

*Colour*. Black; mandible and antenna (except for three black basal segments) dark brown; wing membrane subhyaline, pterostigma and veins brown; mesopleuron red-brown; tegulae dark brown; fore and middle legs dark brown to brown, tibia and basitarsus basally ivory; hind leg black-brown, subbasal patch of hind tibia ivory, tarsus mainly dark brown, basal patch of hind basitarsus ivory in dorsal view; second and third metasomal tergites and basally fourth tergite red-brown.

*Variation*. Body length 12.7–17.0 mm; ovipositor sheath 0.6–0.9 times as long as body, 1.0–1.4 times as long as metasoma, 1.9–2.4 times as long as hind tibia and tarsus combined and 3.0–4.1 times as long as hind tibia. Both female paratypes from Hainan have the pronotum black, head with distinct V-shaped incision and comparatively narrow emargination medio-posteriorly; Other Chinese females have the mesosoma laterally largely red-brown and the incision may be more rounded. Chinese males differ from females by the normally convex vertex and the rounded incision of the head medio-posteriorly (Fig. 61); vertex flat behind ocelli (♀) or weakly convex (♂); pronotal side posteriorly and mesopleuron orange brown or black; mesoscutum coarsely or finely transversely rugose or rugulose (Figs 49, 57), sometimes sculpture obsolete; apical 0.4 of hypopygium of female incised.

#### Distribution.

China (Henan, Fujian, Hainan, Guangxi, Yunnan, Tibet); Burma.

#### Biology.

Unknown. Collected in March, June and July.

#### Notes. 

*Gasteruption bimaculatum* is very similar to *Gasteruption obscuripenne* Pasteels, 1958, a species described from Java (Figs 334–341), but *Gasteruption bimaculatum* has the mesosoma laterally often orange-brown (black in *Gasteruption obscuripenne*), the head less directly narrowed behind the eyes (more so in *Gasteruption obscuripenne*; Fig. 340 versus Fig. 53), the eyes conspicuously setose (but sometimes inconspicuously so; glabrous or inconspicuously setose in *Gasteruption obscuripenne*) and apical 0.4 of hypopygium of female incised (apical 0.3 in *Gasteruption obscuripenne*). Male paratypes of *Gasteruption obscuripenne* from Java have the vertex completely flat and more acutely incised and comparatively slender basal antennal segments. These differences may be clinal, but without studying material from the area between the Sunda region and China (including Himalayas) we refrain from formally synonymising both species.

Two paratypes of *Gasteruption bimaculatum* from Sumba (1 ♀ + 1 ♂ (KBIN) “C. Sumba, Lokojengo, 24.ix. (♀) or 25.ix.1949 (♂), Dr. Bühler, Dr. Sutter”, “*Gasteruption bimaculatum* n. sp., J. Pasteels det., 1955”, “Paratypus”, “R.I.Sc.N.B. I.G. 21280”) have been examined. They belong not to *Gasteruption bimaculatum* but are very close to *Gasteruption obscuripenne*. The Sumba specimens has similar ovipositor sheath (2.2 times as long as hind tibia and tarsus combined, 3.4 times hind tibia, 1.1 times metasoma and 0.7 times body), OOL long (1.4 times diameter of ocellus), brown mandible and coloured wings. However, the Sumba specimens has the head shorter in dorsal view, the propleuron slightly slenderer, the pronotal side distinctly slenderer, the face short and inconspicuously setose, hind femur slightly less slender and hind tarsus distinctly less slender, the pronotum with a distinct antero-lateral tooth and the mesosoma largely orange-brown laterally. The mesosoma is 1.9 times as long as high, 4^th^ and 5^th^ antennal segments of male slender and the propleuron 1.1 times as long as mesoscutum in front of tegulae and hypopygium not slit-shaped incised. The report of *Gasteruption obscuripenne* from the Philippines (Pasteels 1958) will remain doubtful till females are available.

### Gasteruption birmanense

Pasteels, 1958

http://species-id.net/wiki/Gasteruption_birmanense

Figs 63–70

Gasteruption birmanense Pasteels, 1958: 199, fig. 22.

#### Type material.

Holotype, ♀ (NRS), “N.E. Burma, Kambaiti, 7000 ft., 17.V.[1934], R. Malaise”, “*Gasteruption birmanense* n. sp., J. Pasteels det. 1954”, “Holotype”, “NHRS-HEVA 000001978”. Paratype, ♂ (NRS), same data but 18.V.1934, “Allotype” and “NHRS-HEVA 000001979”.

#### Additional material.

1 ♀ (ZJUH), “[China:] Hunan, Shimen, Mt. Huping, 14.VII.2009, Jie Zeng”; 1 ♀ (CSCS), “[China:] Sichuan, Wolong Nature Reserve, 27.VII.1981, Li”.

#### Diagnosis.

**Figures 63–70.**
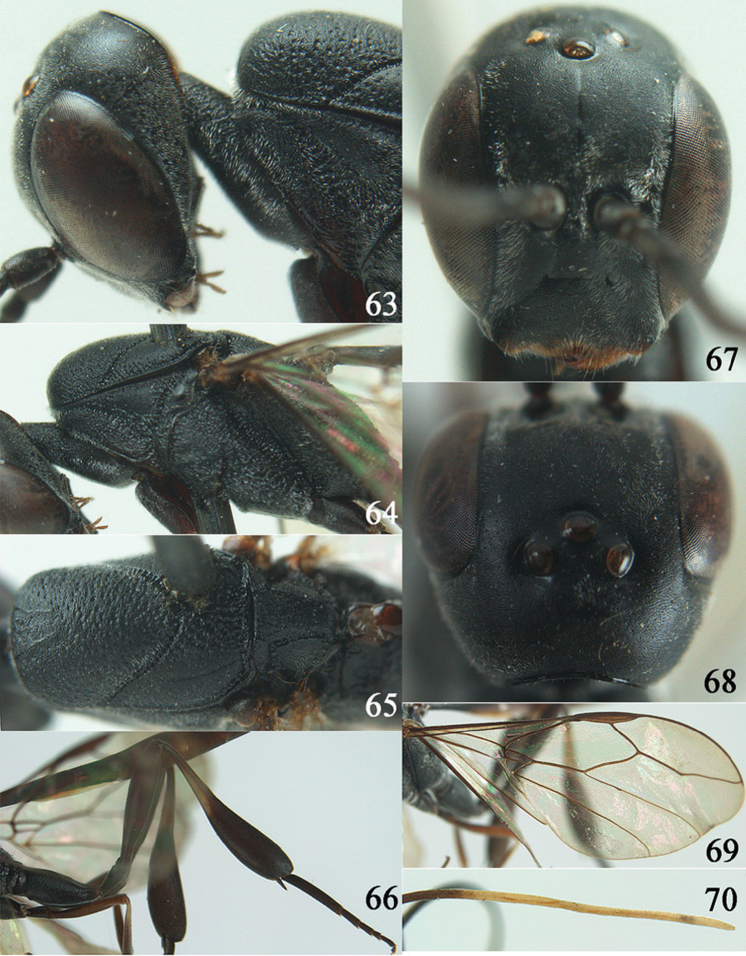
*Gasteruption birmanense* Pasteels, 1958, holotype, female. **63** head lateral **64** mesosoma lateral **65** mesoscutum dorsal **66** hind leg **67** head anterior **68** head dorsal **69** fore wing **70** apex of ovipositor sheath.

Apex of ovipositor sheath widely ivory (Fig. 70), about 2.8–3.1 times as long as hind basitarsus; ovipositor sheath about 0.8–1.1 times as long as body; occipital carina narrow and non-lamelliform medio-dorsally (Fig. 63) and slightly protruding laterally (Fig. 63); propleuron robust, 0.8–1.0 times as long as mesoscutum in front of tegulae (Fig. 64); antesternal carina narrow; head, laterally mesosoma and scape black; head in anterior view not protruding below lower level of eyes and mandibular condylus near lower level of eyes (Fig. 67); in lateral viewcondylarincision of malar space close to eye (Fig. 63); clypeal ventral depression obsolescent and lateral corners rather protruding forwards; eyes glabrous; fourth and fifth antennal segment 1.3–1.6 and 1.3 (♀) times as long as third segment, respectively, (of ♂ antenna missing, but according to original description fourth segment 3.2 times and third segment as long as second segment); apical antennal segment of ♀ 1.4 times as long as third antennal segment and brown, paler than middle segments; mesoscutum and head matt, head dorsally densely coriaceous and finely punctulate; mesoscutum coriaceous between medium-sized punctures, punctate-reticulate medio-posteriorly; hind coxa coriaceous and with some transverse rugulae dorsally; hind tibia rather slender, with a distinct subbasal ivory patch and ventral border convex (Fig. 66); hind basitarsus comparatively long and medially dark brown, as remainder of hind tarsus (Fig. 66); hind tibial spurs dark brown; incision of hypopygium deep, slit-shaped.

#### Description.

Holotype, female, body length 15.1 mm.

*Head*. Vertex and frons matt, densely coriaceous and finely punctulate (Fig. 68), convex medio-posteriorly (Fig. 63); head comparatively weakly narrowed behind eyes; temple 0.6 times as long as eye in dorsal view (Fig. 68); fourth antennal segment 1.6 times as long as third segment and as long as second and third segments combined, fifth antennal segment 1.3 times as long as third segment, third antennal segment 1.8 times as long as second segment; occipital carina narrow and non-lamelliform medio-dorsally; OOL 1.6 times as long as diameter of posterior ocellus; face rather wide (Fig. 67); minimum width of malar space 0.2 times as long as second antennal segment (Fig. 63); clypeus without distinct depression, its lateral corners rather protruding forwards and medio-ventrally slightly emarginate; eye glabrous.

*Mesosoma*. Length of mesosoma 1.9 times its height; pronotal side comparatively elongate (Fig. 64) and ventrally coriaceous and partly rugulose, with a distinct antero-lateral tooth; mesoscutum not protruding anteriorly; propleuron robust, 0.8 times as long as mesoscutum in front of tegulae (Fig. 64); antesternal carina narrow and narrowly lamelliform; mesoscutum densely coriaceous and with many medium-sized punctures (but less so on lateral lobes), matt and medio-posteriorly punctate-reticulate (Fig. 65); scutellum coriaceous.

*Wings*. Fore wing: first discal cell subparallel-sided and with outer posterior corner rounded (Fig. 69), glabrous; vein SR1 weakly bent.

*Legs*. Hind coxa coriaceous and with some transverse rugulae dorsally, slender; length of hind femur, tibia and basitarsus 4.6, 5.2 and 6.7 times their width, respectively (Fig. 66); middle tarsus 1.3 times as long as middle tibia; middle femur subparallel-sided and somewhat slenderer than fore femur.

*Metasoma*. Ovipositor sheath 5.2 times as long as hind tibia, 3.1 times as long as hind tibia and tarsus, 1.4 times metasoma and 1.1 times body; ivory part of sheath 2.8 times as long as hind basitarsus; apical half of hypopygium incised.

*Colour*. Black; mandible largely yellow-brown; antenna dark brown, but apically brown; fore and middle legs brown, but tibiae ivory basally and coxae dark brown; hind leg dark brown but tibia with ivory subbasal patch ventrally; second-third metasomal tergites more or less brown, tegulae largely dark brown; pterostigma brown; wing membrane slightly infuscate.

*Male*. Paratype. Head behind eyes roundly narrowed in dorsal view; occipital carina narrow and non-lamelliform medio-dorsally; face wide; antenna missing but according to original description third antennal segment as long as second segment; fourth segment 3.2 times as long as third segment and 1.6 times as long as second and third segments combined; vertex coriaceous, matt; eye glabrous; frons rather convex; propleuron rather robust and 0.8 times as long as mesoscutum in front of tegulae; antesternal carina non-lamelliform and narrow; mesoscutum densely coriaceous, without distinct punctures and posteriorly rugose and rugulose; hind coxa slender in dorsal view; hind leg coloured as in female; paramere dark brown and apically brown; body length 13.3 mm. Very similar to female, but less coarsely sculptured.

*Variation*. Specimen from Hunan: Body length 10.7 mm, ovipositor sheath 0.8 times as long as body, 1.3 times as long as metasoma and 4.3 times as long as hind tibia; ivory part of sheath 3.1 times as long as hind basitarsus; third antennal segment 1.6 times as long as second segment, fourth antennal segment as long as fifth segment and 1.3 times as long as third segment; propleuron as long as mesoscutum in front of tegulae.

#### Distribution.

Oriental China (Hunan, Sichuan); Burma.

#### Biology.

Unknown. Collected in May and July.

#### Notes.

Very similar to *Gasteruption sinense* var. *minus* Kieffer, 1924 from China, but differs by having the propleuron about 0.8 times mesoscutum in front of tegulae (1.0–1.3 times in *Gasteruption sinense* var. *minus* Kieffer, 1924) and occipital carina narrow medio-dorsally (narrow lamelliform).

### Gasteruption coloratum sp. n.

urn:lsid:zoobank.org:act:F333ED0F-6E1E-4F76-A4B8-0A7BA7E30E09

http://species-id.net/wiki/Gasteruption_coloratum

Figs 71–78

#### Type material.

Holotype, ♀ (ZJUH), “[China:] Xinjiang, Kuerle, 1985, En-zhen Wang”.

#### Diagnosis.

**Figures 71–78.**
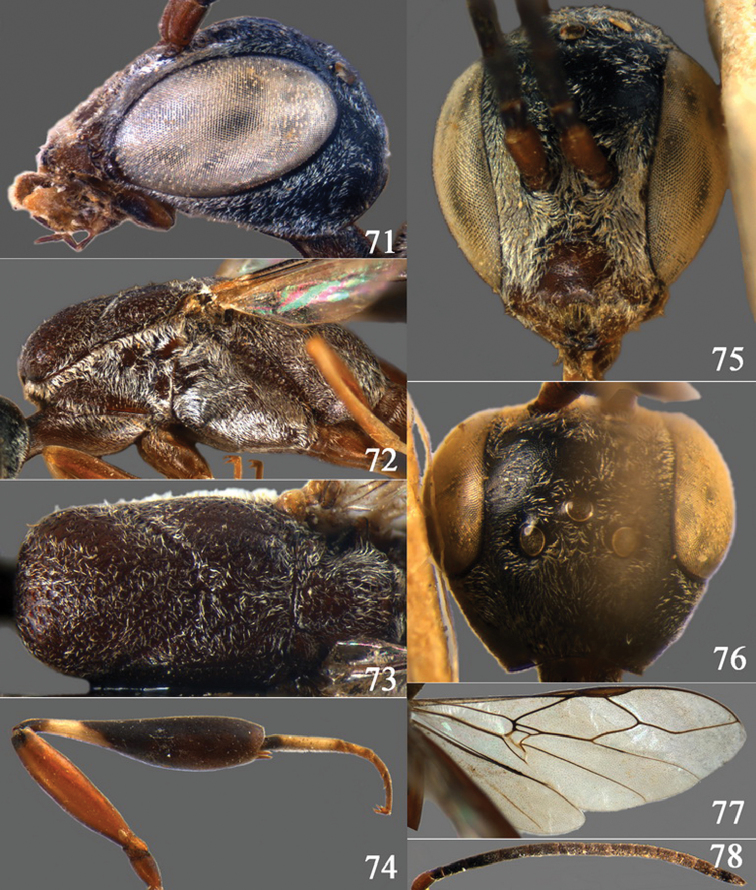
*Gasteruption coloratum* sp. n., holotype, female. **71** head lateral **72** mesosoma lateral **73** mesoscutum dorsal **74** hind leg **75** head anterior **76** head dorsal **77** fore wing **78** antenna.

Head subtruncate medio-posteriorly; posteriorly vertex moderately convex in lateral view (Fig. 71); head black dorsally; hind coxa and femur yellow- or orange-brown; hind tibia black and subbasally ivory and less swollen (Fig. 74); hind basitarsus mainly ivory and only basally dark brown and second segment medially ivory (Fig. 74); metasoma yellow-brown; mesoscutum rather densely setose laterally (Fig. 73); mesopleuron red-brown and weakly sculptured (Fig. 72); ovipositor sheath about 4.0 times as long as hind tibia and tarsus combined, 1.9 times as long as metasoma and about 7.0 times as long as hind tibia; apical 0.5 of hypopygium of female slit-like incised, ; occipital carina obsolescent medio-dorsally (Fig. 76).

#### Description.

Holotype, female, body length 14.0 mm, of fore wing 6.5 mm.

*Head*. Head directly narrowed behind eyes and weakly curved laterally (Fig. 76); temple 0.6 times as long as eye in dorsal view; vertex and frons with satin sheen and rather sparsely very finely punctulate; vertex convex posteriorly (Fig. 71) and without a depression medio-posteriorly (Fig. 76); occipital carina narrow and non-lamelliform medio-dorsally (Fig. 71); third antennal segment 1.7 times as long as second segment; forth antennal segment 1.5 times as long as third segment; fifth antennal segment 1.1 times as long as third segment (Fig. 78); eye setose (Fig. 71); OOL 1.3 times as long as diameter of posterior ocellus; minimum width of malar space 0.2 times as long as second antennal segment; clypeus without directly depression.

*Mesosoma*. Length of mesosoma 2.2 times as long as its height; length of propleuron 0.8 times as long as mesoscutum in front of tegulae (Fig. 72); side of pronotum with satin sheen and sparsely finely punctulate, slightly rugulose, antero-ventrally distinctly rugose especially, with a distinct antero-lateral tooth; mesoscutum with satin sheen and moderately densely punctate, interspaces with small punctulate and finely rugulose (Fig. 73); posteriorly and laterally with rugulose-punctate, scutellum with punctate and irregularly rugulose; propodeum reticulate-rugose, medio-longitudinal carina indistinct.

*Wings*. Fore wing: first discal cell parallel-sided and with outer posterior corner rounded (Fig. 77).

*Legs*. Hind coxa matt, moderately slender and with transversely rugose dorsally; length of hind femur, tibia and basitarsus 4.0, 4.6 and 5.0 times as long as their width, respectively (Fig. 74); middle tarsus 1.3 times as long as middle tibia.

*Metasoma*. Ovipositor sheath 1.3–1.4 times as long as body, 1.9 times as long as metasoma and 7.0 times as long as hind tibia.

*Colour*. Red-brown; head black; antenna (except for first black basal segment) dark brown; mandible orange brown, apex dark brown; legs mainly orange brown, fore and middle tibiae laterally with ivory longitudinal patches; hind tibia black, subbasal patch ivory, basitarsus mainly ivory and basally dark brown, second tarsomere medially ivory; metasoma orange brown; ovipositor sheath black-brown, besides of apically brown; pterostigma dark brown.

*Male*. Unknown.

#### Distribution.

Palaearctic China (Xinjiang).

#### Etymology.

Named after the multi-coloured hind legs; “coloratus” is “coloured” in Latin.

### Gasteruption corniculigerum

Enderlein, 1913

http://species-id.net/wiki/Gasteruption_corniculigerum

Figs 79 94

Gasteruption corniculigerum Enderlein, 1913: 322–323; Hedicke 1939: 27; Pasteels 1958: 176–177, fig. 1.

#### Type material.

Lectotype here designated, ♀ (DEI), “[China:] Formosa [= Taiwan], Taihorin, 7.VIII.1911, H. Sauter”, “Syntypus”, “*Gasteruption corniculigerum* Enderl., ♀, Type, Dr. Enderlein, det. 1913”, “Dtsch. Entomol. Institut Berlin”. Paralectotypes: 1 ♂ (DEI), topotypic and same date. In DEI and PAN are 3 female paralectotypes (from Taihorin, Kosempo and Silam) and 1 male paralectotype (from Kankau or Koshun).

#### Additional material.

1 ♀ (ZJUH), “[China:] Zhejiang, West Mt. Tianmu, 30.V.1998”; 1 ♂ (ZJUH), “[China:] Zhejiang, Songyang, XI.1986, Han-lin Chen”; 1 ♀ (ZJUH), “[China:] Fujian, Fuzhou, 2.X.1983, Nai-quan Lin”; 1 ♂ (ZJUH), “[China:] Fujian, Fuzhou, 13.VIII.1989, Chang-ming Liu”; 1 ♂ (ZJUH), “[China:] Fujian, Fuzhou, 3.X.1990, Nai-quan Lin”; 1 ♀ (ZJUH), “[China:] Fujian, Fuzhou, Jinshan, 23.VI.1986, Jing-zhang Zhao”; 1 ♂ (ZJUH), “[China:] Fujian, Fuzhou, 9.VI.1988, Xiu-fu Zhao”; 1 ♀ (ZJUH), “[China:] Fujian, Fuzhou, Jinshan, 19–26.IV.1990, Xiu-fu Zhao”; 1 ♀ (ZJUH), “[China:] Fujian, Fuzhou, 31.X.1954, H. F. Chao [= Xiu-fu Zhao] Coll”; 1 ♂ (ZJUH), “[China:] Fujian, Nanjing, 28.VII.1988, Xiao-lin Lin”; 1 ♀ (NMNS) “[China:] Taiwan, Taitung, Taiyuan, 21.VII.2000, M.L. Chan”; 1 ♂ (id.), “[China:] Taiwan, Paling, 18.VII.1980 , B. S. Chang”; 1 ♀ (TARI), “[China:] Taiwan, Musha, 25.VI-5.VII.1947, Maa, Chen & Lin”; 1 ♀ (TARI), “[China:] Formosa [= Taiwan], Suo, 19.VII.1938”; 1 ♀ (TARI), “[China:] Taiwan, KAGI, 16.IV.1967, S. Toyota”; 1 ♂ (TARI), [China:] ?Taiwan, Takezaki, 3.V.1931, T. Shiraki”; 1 ♀ (TARI), “[China:] Taiwan, Suisha, 21.I.1927, J. Sonan”; 1 ♂ (TARI), [China:] Taiwan, Funkiko, Arisan, 10.VII.1937, J. Sonan”; 1 ♀ (TARI), “[China:] Taiwan, Koshun, IV.1918, T. Shiraki”; 1 ♂ (TARI), “[China:] Formosa [= Taiwan], Koshun, 25.IV.–25.V.1918, J. Sonan, K. Miyake, M. Yoshino”; 1 ♀ (SCAU), “[China:] Guangdong, Nanling Nature Reserve, 16.X.2007, Zai-fu Xu”; 1 ♂ (ZJUH), “[China:] Guangxi, Tianlin, Qingping, 30.V.1982, Jun-hua He”; 1 ♀ (ZJUH), “[China:] Hainan, Baisha, Mt. Yinggeling, 1–2.V.2008, Jing-xian Liu”; 2 ♀ (SCAU), “[China:] Hunan, Mt. Huping, 11–13.VII.2009, Shi-hong Wang”; 1 ♀ (SCAU), “[China:] Hunan, Mt. Huping, Shinianzigou, 9.VII.2009, Ya-li Tang”; 9 ♀ (SCAU), “[China:] Guizhou, Mayang River, 2.X.2007, Cui-hong Xie”; 4 ♀ (SCAU), “[China:] Guizhou, Daheba, 27.IX.2007, Jie-min Yao”; 2 ♀ (ZJUH), “[China:] Guizhou, Suiyang, Kuankuoshui Nature Reserve, Qinggangtang, 8.VI.2010, Pu Tang”; 1 ♀ (ZJUH), “[China:] Guizhou, Tianzhu, VIII.2009, Yang-wen Wang”.

#### Diagnosis.

**Figures 79–86.**
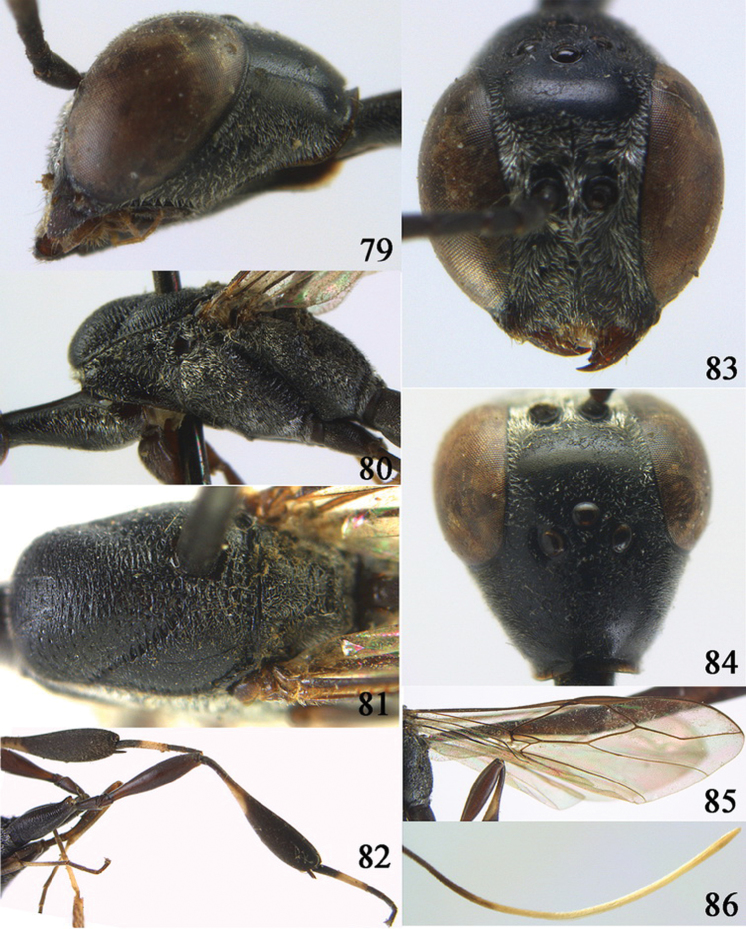
*Gasteruption corniculigerum* Enderlein, 1913, lectotype, female. **79** head lateral **80** mesosoma lateral **81** mesoscutum dorsal **82** hind leg **83** head anterior **84** head dorsal **85** fore wing **86** apex of ovipositor sheath.

**Figures 87–94.**
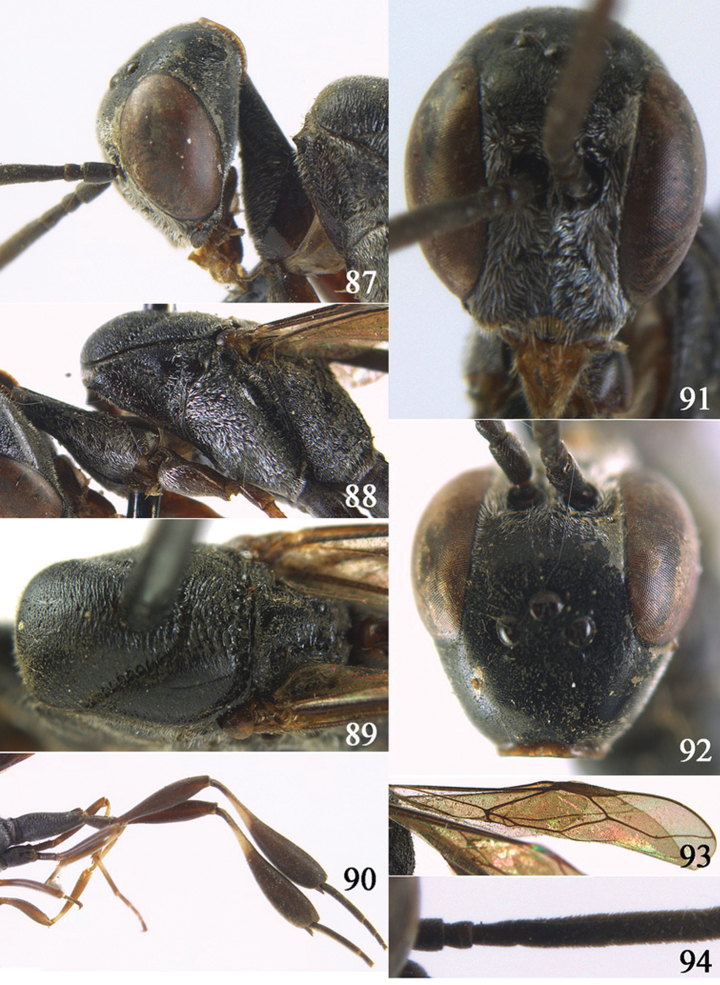
*Gasteruption corniculigerum* Enderlein, 1913, paralectotype, male. **87** head lateral **88** mesosoma lateral **89** mesoscutum dorsal **90** hind legs **91** head anterior **92** head dorsal **93** fore wing **94** the first to fifth antennal segments.

White apical part of ovipositor sheath (Fig. 86) about 2.8–3.5 times as long as hind basitarsus; ovipositor sheath about 1.0–1.1 times as long as body and 1.5–1.8 times as long as metasoma; head directly narrowed in dorsal view (Figs 84, 92); vertex with satin sheen and very finely and sparsely punctulate and in front of occipital carina with shallow triangular depression medio-posteriorly (Figs 84, 92); occipital carina wide and lamelliform medio-dorsally (Figs 79, 87), and distinctly protruding ventro-posteriorly (Figs 79, 87); third antennal segment of ♀ 1.6–1.8 times as long as second segment; frons very finely punctulate, with satin sheen; vertex slightly convex medio-posteriorly and with deep cleft in front of occipital carina in lateral view (Figs 79, 84); propleuron moderately slender and 1.0–1.2 times as long as mesoscutum in front of tegulae (Figs 80, 88); antesternal carina narrow; head in anterior view not protruding below lower level of eyes and mandibular condylus near lower level of eyes (Figs 83, 91); in lateral viewcondylarincision of malar space very close to eye (Figs 79, 87); eyes glabrous; fourth and fifth antennal segment of ♀ 1.3–1.4 (♂ 1.9–2.3) and ♀ 1.1–1.3 (♂ 1.8–2.1) times as long as third segment, respectively (Fig. 94); apical antennal segment of ♀ 1.2 times as long as third antennal segment and its colour similar to colour of medial segments; antenna of female dark brown; mesoscutum with satin sheen and coriaceous with spaced medium-sized crater-like punctures and with coarse transverse rugae medio-posteriorly (Figs 81, 89); lateral lobe of mesoscutum mainly coriaceous, laterally transversely rugulose and with few superficial punctures in lateral view (Figs 81, 89); hind coxa transversely rugose dorsally, mainly coriaceous; hind tibia weakly swollen, resulting in a slightly convex ventral border (Figs 82, 90), with a distinct subbasal ivory patch; apical half of hind basitarsus white (Figs 82, 90); hind tibial spurs dark brown; apical 0.55 of hypopygium incised; head, mesosoma laterally and scape black.

#### Description.

Lectotype, female, body length 16.7 mm.

*Head*. Vertex and frons with satin sheen and very finely densely punctulate; vertex slightly convex in lateral view (Fig. 79), medio-posteriorly with shallow triangular depression and with deep cleft in front of occipital carina (Fig. 84); head directly narrowed behind eyes, subconical (Fig. 84); temple 0.7 times as long as eye in dorsal view; fourth antennal segment 1.4 times as long as third segment and nearly as long as second and third segments combined, fifth antennal segment 1.3 times as long as third segment, third antennal segment 1.8 times as long as second segment; occipital carina wide and lamelliform medio-dorsally (Fig. 79); OOL 1.3 times as long as diameter of posterior ocellus; face rather wide (Fig. 83); malar space partly absent because mandibular condyles reaching eye (Fig. 79); clypeus without depression, its lateral corners not protruding forwards and medio-ventrally slightly emarginate; eye glabrous.

*Mesosoma*. Length of mesosoma twice its height; pronotal side moderately low and ventrally reticulate, but posteriorly largely smooth (Fig. 80), with a small antero-lateral tooth; mesoscutum hardly protruding anteriorly; propleuron comparatively slender, 1.1 times as long as mesoscutum in front of tegulae; antesternal carina narrow and narrowly lamelliform; mesoscutum densely coriaceous and with mostly spaced small to medium-sized and crater-like punctures (Fig. 81) and with satin sheen, medio-posteriorly with coarse transverse rugae (Fig. 81); scutellum superficially coriaceous.

*Wings*. Fore wing: first discal cell parallel-sided and with outer posterior corner hardly angulate (Fig. 85; cf. ♂ Fig. 93); vein SR1 distinctly bent.

*Legs*. Hind coxa with satin sheen, moderately slender, coriaceous and dorsally largely transversely rugose; length of hind femur, tibia and basitarsus 4.4, 4.9 and 6.4 times their width, respectively; hind tibia weakly swollen, resulting in a slightly convex ventral border (Fig. 82); middle tarsus 1.3 times as long as middle tibia; middle femur subparallel-sided and slightly slenderer than fore femur.

*Metasoma*. Ovipositor sheath 1.1 times as long as body, 1.7 times as long as metasoma, 3.5 times as long as hind tibia and tarsus combined and 5.6 times as long as hind tibia; white apical part 3.0 times as long as hind basitarsus and 0.17 times total length of sheath; apical 0.55 of hypopygium slit-shaped incised.

*Colour*. Black or black-brown; mandible, tegulae and legs largely dark brown, but fore and middle tibiae basally, fore basitarsus, basal 0.6 of middle basitarsus, large subbasal patch of hind tibia and apical 0.6 of hind basitarsus ivory; apical sixth of ovipositor sheath widely white; pterostigma dark brown; wing membrane slightly infuscate (less than in male).

*Male*. Paralectotype: head behind eyes more gradually narrowed in dorsal view than in female and shiny (Fig. 92); occipital carina wide lamelliform medio-dorsally (Fig. 87) and medio-posterior depression obsolescent (Fig. 92); face rather narrow (Fig. 91); third antennal segment 1.4 times as long as second segment (Fig. 94); fourth segment 2.3 times as long as third segment, 1.3 times as long as second and third segments combined and fifth segment 2.1 times third segment; propleuron rather slender and 1.1 times as long as mesoscutum in front of tegulae (Fig. 88); hind leg coloured as in female but only basal half of fore basitarsus, basal third of middle tarsus ivory and hind basitarsus dark brown (Fig. 90); genitalia missing; body length 16 mm, of fore wing 6.7 mm. Very similar to lectotype.

*Variation*. Chinese specimens: Female: body length 13.0–20.0 mm, of fore wing 6.7–9.0 mm. Ovipositor sheath 1.0–1.1 times as long as body, 1.5–1.8 times as long as metasoma, 5.2–5.7 times as long as hind tibia; its apical ivory or white part 2.8–3.5 times as long as hind basitarsus; temple 0.6–0.7 times as long as eye in dorsal view; third antennal segment 1.6–1.8 times as long as second segment, fourth antennal segment 1.3–1.4 times as long as third segment, fifth antennal segment 1.1–1.2 times as long as third segment; length of mesosoma 1.9–2.2 times as long as its height; propleuron 1.0–1.2 times as long as mesoscutum in front of tegulae; propodeum reticulate-rugose or reticulate. Male: body length 12.5–17.0 mm, of fore wing 6.0–7.2 mm; temple 0.5–0.6 times as long as eye in dorsal view; third antennal segment 1.2 times as long as second segment, fourth antennal segment 1.9–2.1 times as long as third segment, fifth antennal segment 1.8–2.0 times as long as third segment; length of mesosoma 1.8 times as long as its height; propleuron 1.0–1.1 times as long as mesoscutum in front of tegulae.

#### Distribution.

Oriental China (Zhejiang, Fujian, Taiwan, Hunan, Guangdong, Guangxi, Hainan, Guizhou).

#### Biology.

Unknown. Collected in January, April–October.

### Gasteruption dilutum

Semenov, 1892

http://species-id.net/wiki/Gasteruption_dilutum

Figs 95–102

Gasteryption dilutum Semenov, 1892: 211–212.
Gasteruption dilutum ; Szépligeti 1903: 369; Kieffer 1912: 231, 271; Hedicke 1939: 8.

#### Type material.

Holotype of *Gasteruption dilutum*, ♂ (ZISP), “[NW China: Xinjiang,] Oaz. Sandzhu [= Sandhu oasis], 20.VII.[18]90, (1800 m), [B.] Grombtsjevskij”, round golden label, “*Gasteryption dilutum* m., typ. ♂ un., AS. X.[18]91”.

#### Diagnosis.

**Figures 95–102.**
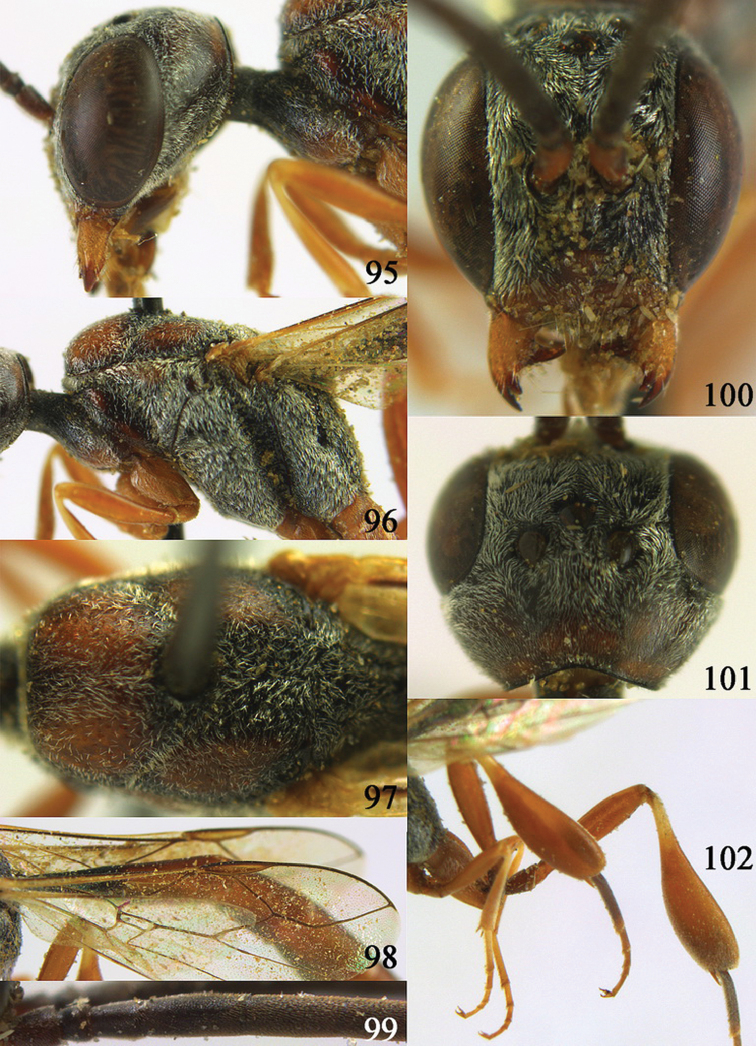
*Gasteruption dilutum* Semenov, 1892, holotype, male. **95** head lateral **96** mesosoma lateral **97** mesoscutum dorsal **98** fore wing **99** the first to fifth antennal segments **100** head anterior **101 **head dorsal **102** hind legs.

Head without a depression in front of occipital carina; propleuron 0.8 times as long as mesoscutum in front of tegulae and robust (Fig. 96); occipital carina distinct but non-lamelliform medio-dorsally (Fig. 95); head directly narrowed behind eyes in dorsal view (Fig. 101); temple 0.7 times as long as eye in dorsal view; OOL (♂) 1.2 times as long as diameter of posterior ocellus; third and fourth antennal segments of female unknown, of male fourth segment 1.8 times as long as third segment and 1.1 times as long as second and third segments combined; fifth antennal segment of female unknown, of male 1.7 times as long as third segment (Fig. 99) and penultimate segments unknown; third antennal segment of male normal, 1.8 times as long as second segment (Fig. 99); vertex finely punctulate and with satin sheen; malar space short (Fig. 95); antero-lateral teeth of pronotum obsolescent, only as angulate corner; mesoscutum robust (Fig. 97), densely setose and partly smooth between separate punctures; marginal cell of fore wing comparatively wide (Fig. 98); hind coxa short and coriaceous, but transversely striate dorsally; hind coxa and femur yellow-brown; pronotal side dorsally and ventrally yellow-brown but medially and posteriorly dark brown; hind basitarsus of male dark brown (but apically somewhat brown) and hind tibia bicoloured (largely yellow-brown, but large ivory basally) and wide (Fig. 102); female tibia unknown; scape and apical half of paramere yellow-brown; colour of ovipositor sheath unknown, but in related species black apically and moderately long.

#### Description.

Holotype, male, body length 12.9 mm.

*Head*. Vertex with satin sheen and finely punctulate, densely setose, flat in lateral view and without depression medio-posteriorly (Fig. 95); head directly narrowed behind eyes (Fig. 101); temple 0.7 times as long as eye in dorsal view; fourth antennal segment 1.8 times as long as third segment and 1.1 times longer than second and third segments combined, fifth antennal segment 1.7 times as long as third segment (Fig. 99), third antennal segment 1.8 times as long as second segment; occipital carina non-lamelliform and distinct medio-dorsally; eyes glabrous; ocelli large, OOL 1.2 times as long as diameter of posterior ocellus and POL twice diameter; face moderately wide (Fig. 100); clypeus flat and laterally not protruding; malar space 0.2 times as long as second antennal segment (= pedicellus).

*Mesosoma*. Length of mesosoma 1.6 times its height; pronotal side normal, ventrally partly smooth, medially crenulate and punctulate dorsally; antero-lateral teeth of pronotum as angulate corners; mesoscutum not protruding anteriorly; propleuron robust and 0.8 times as long as mesoscutum in front of tegulae (Fig. 96); antesternal carina narrow and non-lamelliform; middle and lateral lobes of mesoscutum shiny, densely setose, partly smooth and punctulate between medium-sized punctures and postero-medially reticulate-punctate (Fig. 97); scutellum partly smooth and with few large punctures; propodeum reticulate and densely setose, no median carina; nearly entire mesopleuron coarsely reticulate and densely silvery setose.

*Wings*. First discal cell parallel-sided and no distinct distal posterior corner and its base vertical (Fig. 98).

*Legs*. Hind coxa with satin sheen and punctulate, but transversely striate dorsally; length of hind femur, tibia and basitarsus 5.0, 3.6 and 6.6 times their width, respectively; hind tibia wide (Fig. 102), hind basitarsus parallel-sided in lateral view, but distinctly widened basally in dorsal view.

*Metasoma*. Apical half of parameres of other specimens yellow-brown.

*Colour*. Black or dark brown; vertex partly brown (Fig. 101); clypeus, mandible, scape, pronotal side dorsally and ventrally, mesoscutum (except medio-posteriorly), metasoma (except first segment), hind tibial spurs and legs (but base of tibiae ivory and hind basitarsus dark brown except its apex) yellow-brown; pterostigma pale brown, but laterally darkened; wings subhyaline.

#### Distribution.

Palaearctic China (Xinjiang).

#### Biology.

Unknown. Collected in July at 1800 m.

### Gasteruption dimidiatum

Semenov, 1892

http://species-id.net/wiki/Gasteruption_dimidiatum

Figs 103–110

Gasteryption dimidiatum Semenov, 1892: 210–211.
Gasteruption dimidiatum ; Szépligeti 1903: 369; Kieffer 1912: 229, 263; Hedicke 1939: 9.

#### Type material.

Holotype of *Gasteruption dimidiatum*, ♀ (ZISP), “79396” [= Uzbekistan, Amu Darja River, Petro-Alexandrovsk (= Turtkul), VI.1875, Mielberg]”, round golden label, “*Gasteryption dimidiatum* m., typ. ♂ un., AS. XI.[18]91”.

#### Additional material.

1 ♀ (ZJUH). “[China:] Xinjiang, Urumqi, 14–21.VII.1991, Jun-hua He”; 1 ♀ (ZJUH), “[China:] Xinjiang, Qitai, V.1986”; 1 ♀ (RMNH), “[China:] Xinjiang, Urumqi, 1978, Wen-liang Ma”; 1 ♀ (ZJUH), “[China:] Xinjiang, Shihezi, 1981, Fu-de He”; 1 ♀ (ZJUH), “[China:] Xinjiang, Aertai, Jian-xin Kang”.

#### Diagnosis.

**Figures 103–110.**
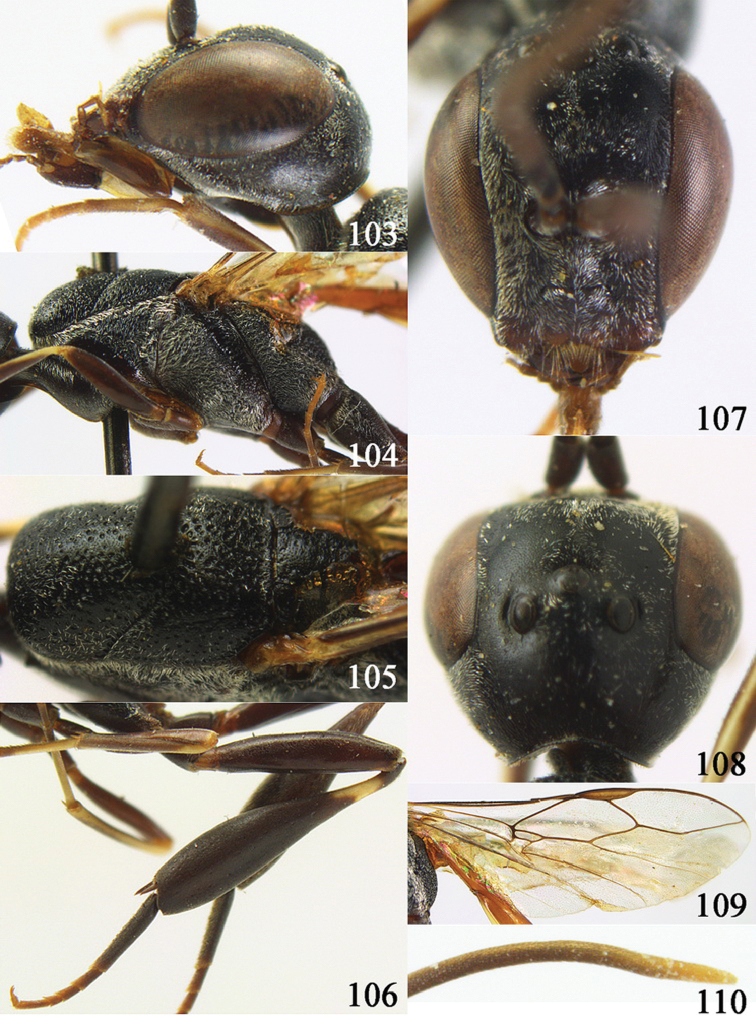
*Gasteruption dimidiatum* Semenov, 1892, holotype, female. **103** head lateral **104** mesosoma lateral **105** mesoscutum dorsal **106** hind leg **107** head anterior **108** head dorsal **109** fore wing **110** apex of ovipositor sheath.

Apex of ovipositor sheath narrowly ivory (Fig. 110), at most 0.3 times as long as hind basitarsus; ovipositor sheath 1.0–1.3 as long as body; occipital carina narrow, non-lamelliform medio-dorsally (Fig. 103) and protruding laterally (Fig. 103); propleuron robust, 0.7 times as long as mesoscutum in front of tegulae (Fig. 104); antesternal carina narrow; head, laterally mesosoma and scape black; head in anterior view not protruding below lower level of eyes (Fig. 107); in lateral viewcondylarincision of malar space close to eye (Fig. 103); clypeal ventral depression obsolescent and lateral corners hardly protruding; eyes glabrous; fourth and fifth antennal segment 1.1 and 1.0 (♀; of ♂ unknown) times as long as third segment, respectively; apical antennal segment of ♀ 1.2 times as long as third antennal segment and dark brown, as colour of medial segments; mesoscutum and head with satin sheen, head dorsally very finely punctulate and with satin sheen (Fig. 108); mesoscutum partly smooth or punctulate between numerous large punctures, rugose-punctate medio-posteriorly (Fig. 105); hind coxa transversely rugulose dorsally (but posteriorly transversely striate), laterally densely punctulate; hind tibia slender, with ivory ring subbasally, with a moderately convex ventral border (Fig. 106); hind basitarsus moderately long and entirely dark brown (Fig. 106), in dorsal view distinctly widened basally; hind tibial spurs dark brown; first metasomal segment orange or yellow-brown; apical 0.6 of hypopygium incised.

#### Description.

Holotype, female, body length 12.6 mm.

*Head*. Vertex and frons with satin sheen and densely and very finely punctulate (Fig. 108), moderately convex medio-posteriorly (Fig. 103); head rather directly narrowed behind eyes and U-shaped emarginate medio-posteriorly (Fig. 108); temple 0.6 times as long as eye in dorsal view; fourth antennal segment 1.1 times as long as third segment and 0.8 times as long as second and third segments combined, fifth antennal segment as long as third segment, third antennal segment 2.6 times as long as second segment; occipital carina narrow, non-lamelliform medio-dorsally; OOL 1.2 times as long as diameter of posterior ocellus; face rather wide (Fig. 107); minimum width of malar space 0.4 times as long as second antennal segment (Fig. 103); clypeus without distinct depression, its lateral corners hardly protruding forwards and medio-ventrally emarginate; eye glabrous.

*Mesosoma*. Length of mesosoma 1.8 times its height; pronotal side comparatively low and largely coriaceous, medially crenulate and with small antero-lateral tooth; mesoscutum not protruding anteriorly; propleuron robust, 0.7 times as long as mesoscutum in front of tegulae (Fig. 104); antesternal carina narrow and hardly lamelliform; mesoscutum partly smooth or punctulate between numerous large punctures and rugose-punctate medio-posteriorly, with satin sheen, rather matt (Fig. 105); scutellum punctulate and with some punctures, shiny; meso- and metapleuron densely silvery setose.

*Wings*. Fore wing: first discal cell subparallel-sided and with outer posterior corner rounded (Fig. 109), glabrous; vein SR1 distinctly bent; marginal cell rather slender (Fig. 109).

*Legs*. Hind coxa with satin sheen, rather wide, transversely rugulose dorsally (but posteriorly transversely striate), laterally densely punctulate; length of hind femur, tibia and basitarsus 4.5, 4.5 and 5.5 times their width, respectively (Fig. 106); hind tibia 1.1 times as long as hind femur and trochanter combined; middle tarsus 1.2 times as long as middle tibia; middle femur subparallel-sided and slenderer than fore femur.

*Metasoma*. Ovipositor sheath 5.0 times as long as hind tibia, 3.8 times as lomg as hind tibia and basitarsus combined, 1.6 times metasoma and as long as body; ivory part of sheath 0.3 times as long as hind basitarsus; apical 0.6 of hypopygium slit-like incised.

*Colour*. Black or dark brown; base of fore and middle tibiae and most of fore and middle basitarsi and subbasal ring of hind tibia ivory; remainder of hind leg (including basitarsus) mainly dark brown; clypeus ventrally, metasoma yellow-brown; pterostigma pale brown, but laterally darkened; wing membrane subhyaline.

*Variation*. Chinese specimens: Female: body length 12.6–15.5 mm, ovipositor sheath 1.0–1.3 times as long as body, 1.5–1.9 times as long as metasoma, 2.9–3.6 times as long as hind tibia and tarsus combined and 4.4–6.1 times as long as hind tibia; temple 0.5–0.6 times as long as eye in dorsal view; third antennal segment 1.6–1.7 times as long as second segment, fourth antennal segment 1.3–1.4 times as long as third segment, fifth antennal segment 1.0–1.1 times as long as third segment; length of mesosoma 1.8–2.1 times as long as its height; propleuron robust, 0.7–0.8 times as long as mesoscutum in front of tegulae; medio-posteriorly of mesoscutum with medium-sized punctate or coalescent punctates, laterally sparsely punctate or punctate-rugose; length of hind femur, tibia and basitarsus 3.9–4.1, 4.5–4.9 and 5.0–5.8 times their width, respectively; middle tarsus 1.2–1.3 times as long as middle tibia; colour of body mainly black or dark brown; frist tergite orange-brown or black.

#### Distribution.

Palaearctic China (Xinjiang); Uzbekistan.

#### Biology. 

Unknown. Collected in July and November.

### Gasteruption formilis

Alekseev, 1995

http://species-id.net/wiki/Gasteruption_formilis

Figs 111 123

Gasteruption formilis Alekseev, 1995: 42 (in key only).

#### Type material.

Not available.

#### Other material.

1 ♀ + 1 ♂ (ZJUH), “[China:] Gansu, Zhenyuan, VII.1981, Wei Cao”; 1 ♂ (ZJUH), “[China:] Xinjiang, Aletai, 25.VII.1990, Deng-yuan Wang”; 1 ♂ (ZJUH), “[China:] Xinjiang, Tacheng, 11.VII.1988, Qi Ma”.

#### Diagnosis.

**Figures 111–118.**
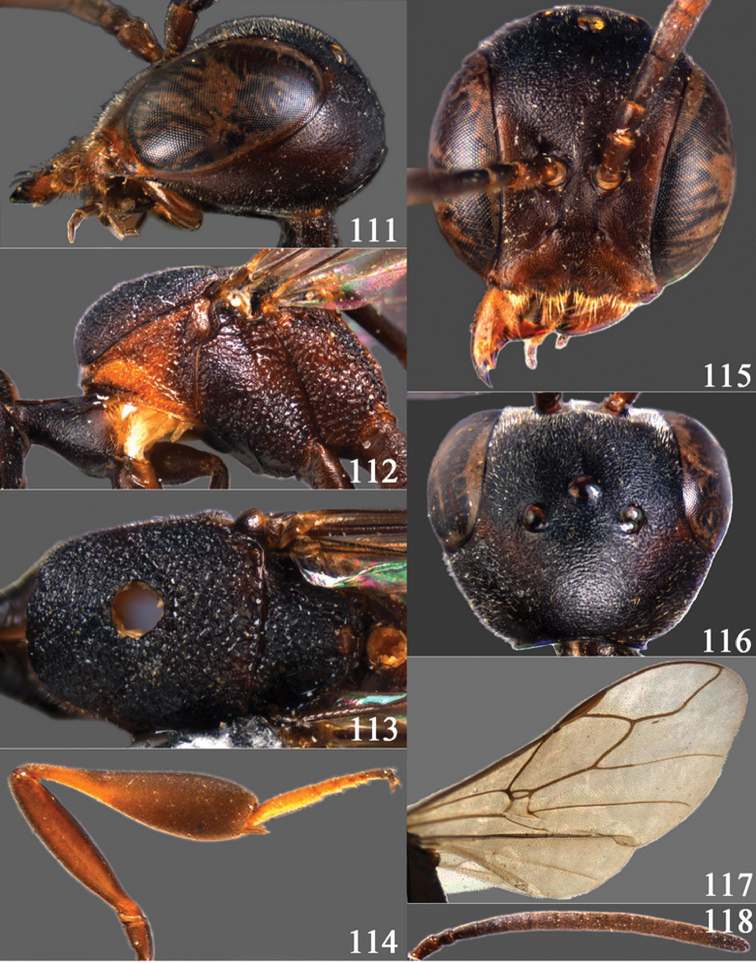
*Gasteruption formilis* Alekseev, 1995, female, Gansu. **111** head lateral **112** mesosoma lateral **113** mesoscutum dorsal **114** hind leg **115** head anterior **116** head dorsal **117** fore and hind wings **118** antenna.

Ovipositor sheath 0.5–0.6 times as long as hind tibia and tarsus combined, about 0.4 times as long as metasoma and as long as hind tibia; hypopygium comparatively shallowly emarginate posteriorly; apex of ovipositor sheath black or dark brown; mesoscutum rather finely and very irregularly rugulose or rugose (Fig. 113; males have rugose, females anteriorly rugulose); hind tibia about 1.2 times as long as hind femur and trochanter and less swollen (Fig. 114); hind basitarsus comparatively short and wide (Fig. 114); head truncate medio-posteriorly or nearly so; vertex and frons matt and more or less coriaceous (Fig. 116); occipital carina obsolescent to narrowly lamelliform medio-dorsally (Fig. 111); clypeus with large shallow depression up to dorsal third (Fig. 115, 122); apical antennal segment 1.4–1.5 times third antennal segment; malar space 0.1–0.3 times as long as second antennal segment (= pedicellus); propleuron 0.7–0.8 times as long as mesoscutum in front of tegulae, robust (Fig. 112); median carina of propodeum absent (but sometimes a slightly elevated smooth median line).

#### Description.

Described from a female from Gansu (Zhenyuan), body length 8.5 mm, of fore wing 4.5 mm.

*Head*. Head directly narrowed behind eyes and weakly curved laterally (Fig. 116); temple 0.6 times as long as eye in dorsal view; vertex and frons matt and finely rugulose; vertex moderately convex posteriorly (Fig. 111) and without depression medio-posteriorly (Fig. 116); occipital carina narrow and non-lamelliform medio-dorsally (Fig. 111); third antennal segment 1.5 times as long as second segment, fourth antennal segment as long as third segment, fifth antennal segment 0.9 times as long as third segment (Fig. 118); eye glabrous; OOL 1.7 times as long as diameter of posterior ocellus; minimum width of malar space 0.2 times as long as second antennal segment; clypeus with a distinct depression (Fig. 115).

*Mesosoma*. Length of mesosoma 1.7 times as long as its height; propleuron with rugulose, 0.8 times as long as mesoscutum in front of tegulae (Fig. 112); side of pronotum reticulate-rugose or rugose; mesoscutum and scutellum rugose (Fig. 113); propodeum reticulate-rugose, medio-longitudinal carina indistinct.

*Wings*. Fore wing: first discal cell small and subtriangular, m-cu short (Fig. 117).

*Legs*. Hind coxa robust and dorsally rugose; length of hind femur, tibia and basitarsus 4.3, 3.7 and 4.0 times their width, respectively (Fig. 114); middle tarsus 1.1 times as long as middle tibia; hind tibia 2.0 mm.

*Metasoma*. Ovipositor sheath 0.2 times as long as body, 0.4 times as long as metasoma, 0.5–0.6 times as long as hind tibia and tarsus combined and as long as hind tibia; hypopygium v-shaped incised apically.

*Colour*. Dark brown; pronotum red; mesoscutum black-brown; medio-longitudinal part of propodeum red; second-fifth metasomal tergites with red band; wing membrane slightly infuscate; pterostigma dark brown.

**Figures 119–123.**
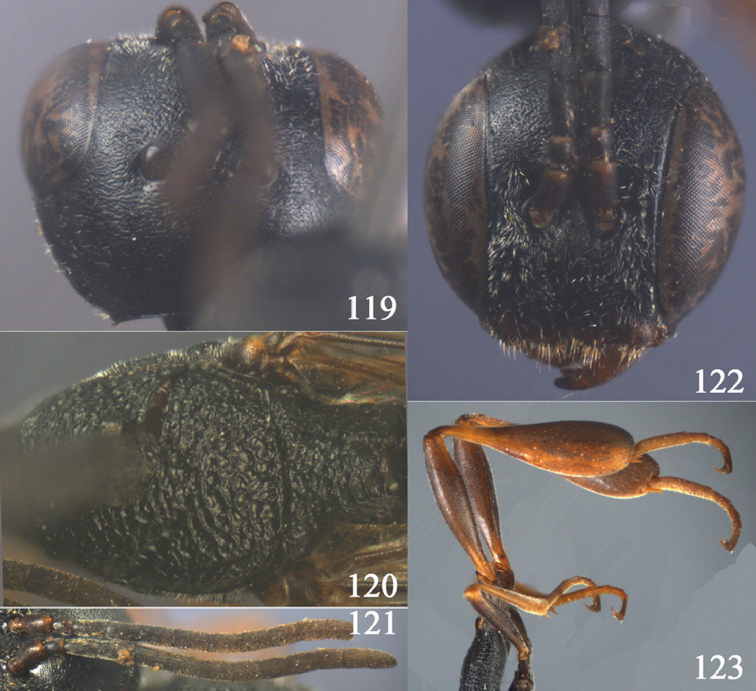
*Gasteruption formilis* Alekseev, 1995, male, Xinjiang. **119** head dorsal **120** mesoscutum dorsal **121** antennae **122** head anterior **123** hind legs.

*Male* (described after a male from Xinjiang). Body length 10.0 mm; temple 0.75 times as long as eye in dorsal view (Fig. 119); third antennal segment 1.5 times as long as second segment, fourth and fifth antennal segment as long as third segment (Fig. 121); OOL 1.3 times as long as diameter of posterior ocellus; minimum width of malar space 0.2 times as long as second antennal segment; length of mesosoma 1.7 times as long as its height; side of pronotum reticulate-rugose or rugose; mesoscutum coarsely rugose, posteriorly coarsely reticulate-rugose (Fig. 120); scutellum rugose; medio-longitudinal carina of propodeum indistinct; length of hind femur, tibia and basitarsus 4.3, 3.7 and 5.0 times their width, respectively (Fig. 123); middle tarsus 1.1 times as long as middle tibia; colour of body black; mandible dark brown; legs (except for coxae black) dark brown; matasoma dark brown, but second-fifth metasomal tergites apically red.

*Variation*. Male: Body length about 10.0–11.5 mm.

#### Distribution.

Palaearctic China (Gansu, Xinjiang).

#### Biology.

Unknown. Collected in July.

### Gasteruption formosanum

Enderlein, 1913

http://species-id.net/wiki/Gasteruption_formosanum

Figs 124 141

Gasteruption formosanum Enderlein, 1913: 326; Hedicke 1939: 27; Pasteels 1958: 188–189, fig. 13.

#### Type material.

Holotype, ♂ (DEI), “[China:] Formosa [= Taiwan], Taihorin [in Chiayi county], 1911, H. Sauter”, “7.VIII.”, “Holotypus”, “*Gasteruption formosanum* Enderl., ♂, Type, Dr. Enderlein, det. 1913”, “Dtsch. Entomol. Institut Berlin”.

#### Additional material.

1 ♂ (RMNH), “[China:] Zhejiang, Mt. Tianmu, 15.ix.1947”; 2 ♀ + 2 ♂ (ZJUH, RMNH), “[China:] Fujian, Fuzhou, 25.IV.1991, Chang-ming Liu”; 1 ♀ (TARI), “ [China:] Taiwan, Taipei, 21.V.1959, S.C. Chui”; 1 ♀ (TARI), “ [China:] Taiwan, Rokkiri, 15.III.1918, T. Shiraki”; 1 ♀ (RMNH), “[China:] Hunan, Linyang, near Changsha, 30.VI.1985, RMNH’11”.

#### Diagnosis.

**Figures 124–132.**
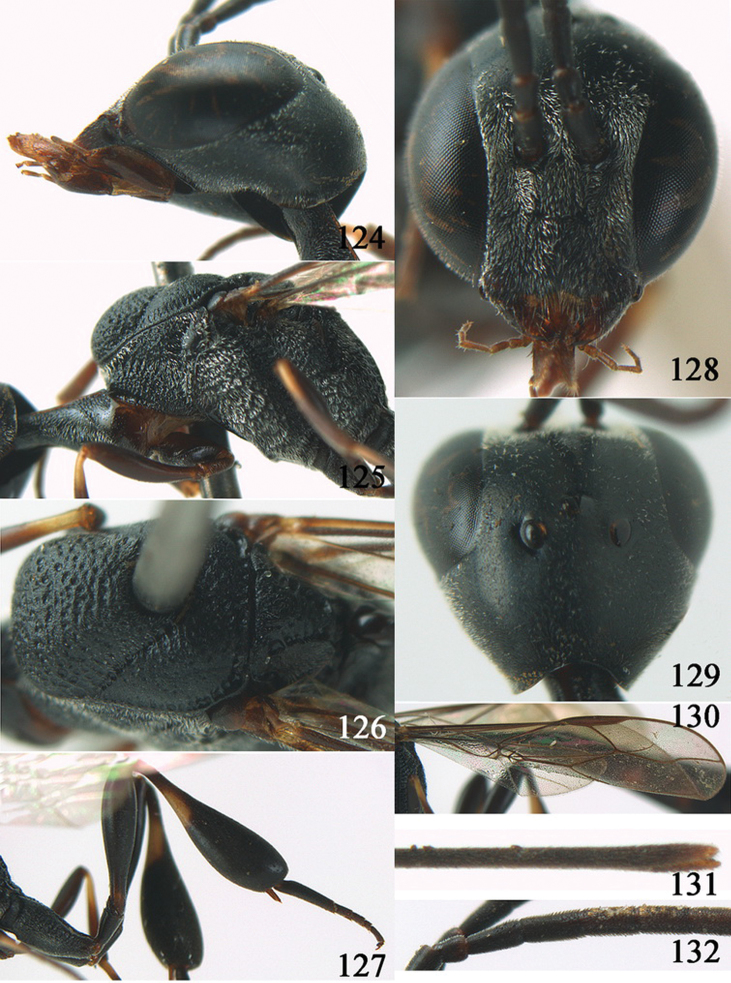
*Gasteruption formosanum* Enderlein, 1913, female, Taiwan. **124** head lateral **125** mesosoma lateral **126** mesoscutum dorsal **127** hind leg **128** head anterior **129** head dorsal **130** fore wing **131** apex of ovipositor sheath **132** the first to sixth antennal segments.

**Figures 133–141.**
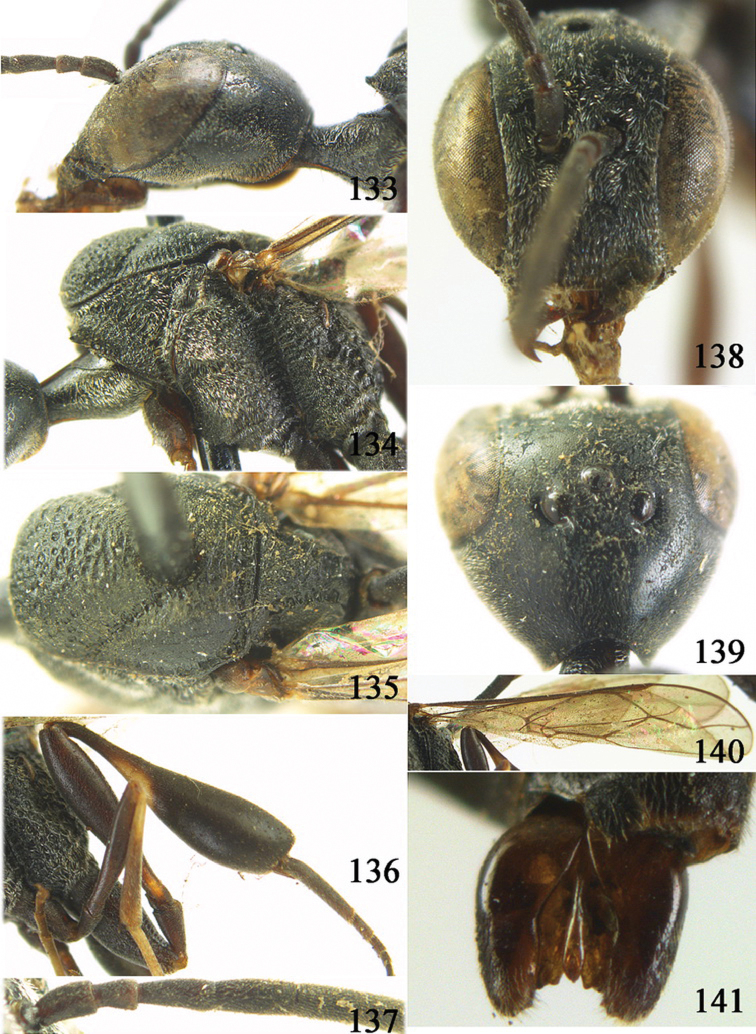
*Gasteruption formosanum* Enderlein, 1913, holotype, male. **133** head lateral **134** mesosoma lateral **135** mesoscutum dorsal **136** hind leg **137** the first to fifth antennal segments **138** head anterior **139** head dorsal **140** fore wing **141** copulatory organ.

Apex of ovipositor sheath black or dark brown (Fig. 131); ovipositor sheath about 0.8 times as long as hind tibia and tarsus combined, about 0.4 times as long as metasoma and about 1.3 times as long as hind tibia; head somewhat elongate in anterior and dorsal view, emarginate medio-posteriorly; first discal cell of fore wing subparallel-sided (Figs 130, 140); occipital carina narrow and non-lamelliform medio-dorsally (Figs 124, 133); antesternal carina narrow; mesoscutum very finely and densely coriaceous, matt and with coarse, more or less isolated punctures (Figs 126, 135), smaller and sparser on lateral lobes and medio-posteriorly with coarse transverse rugae; hind tibia robust and swollen, about as long as hind femur and trochanter combined (Figs 127, 136); hind basitarsus comparatively short, widened basally and black; malar space 0.3 times as long as second antennal segment (= pedicellus); propodeum regularly coarsely reticulate or scrobiculate, median carina of propodeum as a slightly elevated coriaceous median line; eyes with short setae; pronotum with small tooth antero-laterally; vertex and frons shiny and very finely punctulate; propleuron 0.8–0.9 times as long as mesoscutum in front of tegulae and robust (Figs 125, 134); notauli distinct, finely crenulate and medium-sized (Figs 126, 135); hind tibia with pale subbasal patch; hind basitarsus black; apical 0.2 of hypopygium of ♀ incised; apex of ovipositor narrow and acute.

#### Description.

Holotype, male, body length 12.8 mm, of fore wing 6.1 mm.

*Head*. Head distinctly incised medio-posteriorly (Fig. 139); vertex and frons with shiny, largely smooth and very finely punctulate (Fig. 139), moderately convex (Fig. 133) and without a depression medio-posteriorly (Fig. 139); head directly narrowed behind eyes; temple nearly as long as eye in dorsal view (Fig. 139); fourth antennal segment 1.1 times as long as third segment and 0.7 times as long as second and third segments combined, fifth antennal segment nearly as long as third segment (Fig. 137), third antennal segment 1.9 times as long as second segment and moderately slender (Fig. 137); occipital carina narrow and non-lamelliform medio-dorsally (Fig. 133); ocelli comparatively small, OOL 1.5 times as long as diameter of posterior ocellus; face moderately wide (Fig. 138); minimum width of malar space 0.4 times as long as second antennal segment (Fig. 133); clypeus without triangular depression and slightly emarginate; eye with short setae.

*Mesosoma*. Length of mesosoma 1.3 times its height; pronotal side short triangular and largely coarsely reticulate-rugose (as mesopleuron), antero-laterally only angularly protruding; mesoscutum slightly protruding anteriorly; propleuron wide (Fig. 134) and 0.8 times as long as mesoscutum in front of tegulae; antesternal carina narrow and hardly lamelliform; mesoscutum very finely and densely coriaceous, matt and with large, more or less isolated punctures (Fig. 135), smaller and sparser on lateral lobes and medio-posteriorly and near lateral margin with coarse transverse rugae; scutellum coarsely punctate and superficially coriaceous; propodeum coarsely reticulate or scrobiculate, median carina of propodeum as a slightly elevated coriaceous median line.

*Wings*. First discal cell parallel-sided and with outer posterior corner rounded (Fig. 140).

*Legs*. Hind coxa rather matt, moderately stout, transversely rugose and coriaceous; length of hind femur, tibia and basitarsus 4.2, 4.0 and 5.0 times their width, respectively; hind tibia swollen, sparsely punctate and as long as hind tibia and trochanter combined; hind basitarsus rather short and somewhat widened basally (Fig. 136); middle tarsus 1.1 times as long as middle tibia; middle femur about as slender as fore femur.

*Metasoma*. Paramere dark brown (Fig. 141).

*Colour*. Black or black-brown; tegulae and legs (including hind tibial spurs) dark brown or nearly so, but hind tibia with subbasal ivory patch ventrally, fore and middle tibiae basally and fore and middle basitarsi (except apically) pale yellow; wing membrane slightly infuscate; pterostigma dark brown.

*Female* (described after a female from Taiwan). Head behind eyes rather directly narrowed in dorsal view (Fig. 129); occipital carina narrow and non-lamelliform medio-dorsally (Fig. 124); face comparatively narrow (Fig. 128); third antennal segment about twice as long as second segment (Fig. 132); fourth segment 1.1 times as long as third segment, fifth segment as long as third segment, and 0.7 times as long as second and third segments combined; vertex with satin sheen and finely punctulate; eye short setose; frons normal and anterior ocellus near upper level of frons (Fig. 129); propleuron robust and 0.9 times as long as mesoscutum in front of tegulae (Fig. 125); mesoscutum densely coriaceous, between coarse punctures and medio-posteriorly distinctly punctate-rugose (Fig. 126); hind coxa moderately robust in dorsal view; hind basitarsus distinctly widened basally, especially in dorsal view; hind leg dark brown, but with ivory patch ventrally near basal 0.4 of hind tibia (Fig. 127); apical half of first metasomal tergite and second tergite dark brown; body length 13.6 mm; ovipositor sheath 0.2 times as long as body, 0.4 times as long as metasoma and 1.3 times as long as hind tibia; sheath apically dark brown; hypopygium v-shaped incised apically.

#### Distribution.

China (Zhejiang, Fujian, Taiwan, Hunan).

#### Biology.

Unknown. Collected in March–May and September.

### Gasteruption japonicum

Cameron, 1888

http://species-id.net/wiki/Gasteruption_japonicum

Figs 142 152

Gasteruption japonicum Cameron, 1888: 134.
Gasteruption sinense var. *minus* Kieffer, 1924: 78. syn. n.

#### Type material. 

Holotype, ♀ (BMNH), [Japan, Honshu, Kobe, G. Lewis], “Type”, B.M. Type 3.a.166”, “*Gasteruption japonicum* Cam., Type / 97.11”. Syntypes of *Gasteruption sinense* var. *minus* from China (Zi-ka-wei [= Xujiahui, Shanghai], 1 ♀ (15.VI.) + 1 ♂ (6.VI.), O. Piel) not found in O. Piel collection (TARI), MNHN, Entomological Museum Shanghai or Chinese Academy of Sciences Beijing. Probably lost unless present in remnants of Kieffer collection.

#### Additional material.

Topotypical series of var. *minus* (TARI, RMNH) from Shanghai (Zi-ka-wei) collected by O. Piel in IX.1890, V.1918, V.1925, VI.1926 and 1930–31; 2 ♂ (TARI), “Heirin BI, 24.VI.1926, J. Sonan”; 1 ♀ (ZJUH), “[China:] Jilin, Changchun, Jingyue Pool, 7.VIII.1980, En-yu Yu”; 1 ♀ (TARI), “[China:] Zhejiang, Mt. Tianmu, 24.V.[19]37, O. Piel, Musée Heude”; 1 ♀ (ZJUH), “[China:] Fujian, Nanping, Mt. Mangdang, IX. 2002, Chang-ming Liu”; 2 ♂ (TARI), “[China:] ?Taiwan, Makazayazaya, Heito, 9.X.1926, J. Sonan”; 1 ♀ (TARI), “[China:] N. Taiwan, Taipei, 2.XII.1968, K.H. Chang”; 1 ♀ (TARI), “[China:] Formosa [=Taiwan], Mizuho, 23.III.1935, M. Chujo”; 1 ♀ (ZJUH), “[China:] Inner Mongolia, Mt. Helan, Shuimogou, 30.VII.2010, Cheng-jin Yan”; 1 ♀ (CAU) “[China] Shaanxi, Ganquan, Qingquangou, 16.VIII.1971, Ji-kun Yang”; 1 ♀, (CAU). “[China:] Shaanxi, Nanzheng, 1630 m, 23.VII.1985, Fa-sheng Li”; 2 ♀ (ZJUH), “[China:] Ningxia, Mt. Liupan, 21.VI.2008, Jie-min Yao”; 1 ♀ (ZJUH), “[China:] Gansu, Zhenyuan, VII.1981, Wei Cao”; 1 ♀ (ZJUH), “[China:] Xinjiang, Kuerle, V.1985, Jiang-lin Li”; 1 ♀ (SCAU), “[China:] Hubei, Huangmei, 25.VIII.2009, Qi Yang; 1 ♀, (ZJUH), “[China:] Hunan, Panyang, Yichang, XI.1955”; 1 ♀ (SCAU), “[China:] Sichuan, Pingwu, Baimazhai, 25.VI.2006, Hong-ying Zhang; 2 ♀ + 1 ♂ (CAU), “[China:] Yunnan, Kunmingxinshe, 16.VII.1940 & 12.VII.1940”; 1 ♀ (CAU) “[China:] Yunnan, Kunming, Agricultural University, IV.1942”; 1 ♀ (ZJUH), “[China:] Yunnan, Panxi, 27.VII.1983, Yi-chang Liao.

#### Diagnosis.

**Figures 142–149.**
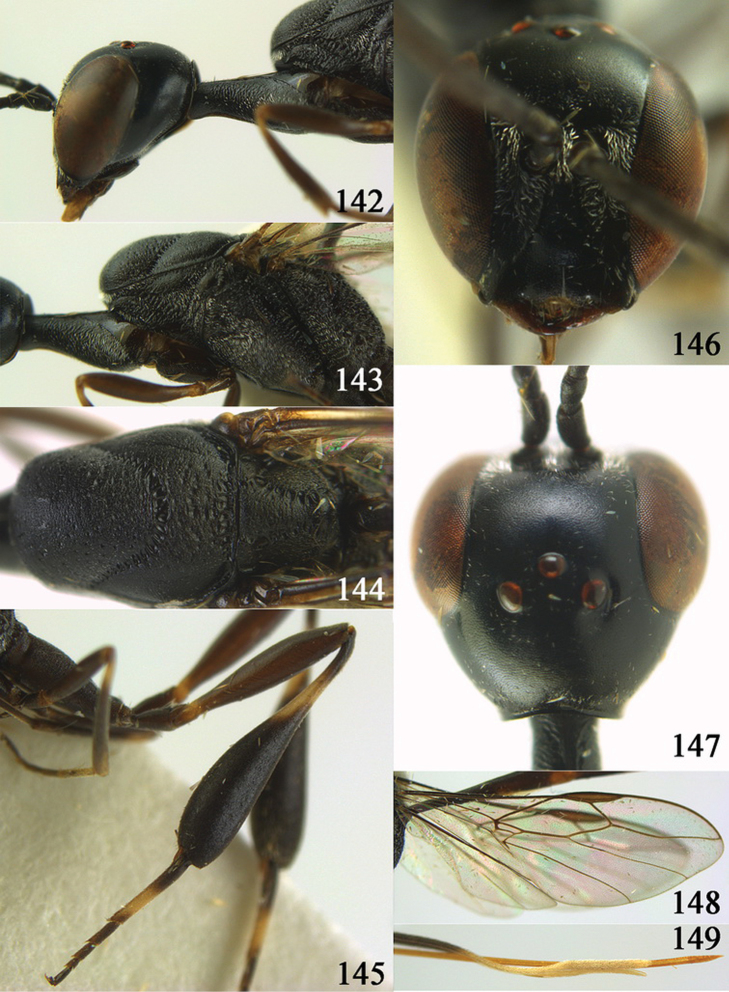
*Gasteruption japonicum* Cameron, 1888, holotype, female. **142** head lateral **143** mesosoma lateral **144** mesoscutum dorsal **145** hind legs **146** head anterior **147** head dorsal **148** fore and hind wings **149** apex of ovipositor and its sheath.

**Figures 150–152.**
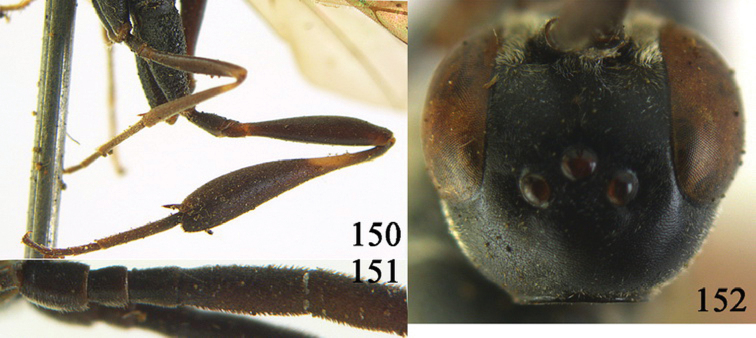
*Gasteruption japonicum* Cameron, 1888, male, Zhejiang. **150** hind leg **151** the first to fourth antennal segments **152** head dorsal.

White apical part of ovipositor sheath (Fig. 149) 1.4–2.0 times as long as hind basitarsus; ovipositor sheath 0.9–1.1 times as long as body; occipital carina narrow lamelliform medio-dorsally (Fig. 142) and rather protruding ventro-posteriorly (Fig. 142); propleuron comparatively slender, 0.9–1.1 times as long as mesoscutum in front of tegulae (Fig. 143); antesternal carina narrow; head, laterally mesosoma and scape black; head in anterior view slightly protruding below lower level of eyes by less than basal width of mandible and mandibular condylus near lower level of eyes (Fig. 146); in lateral viewcondylarincision of malar space close to eye (Fig. 142); clypeus with small depression or depression obsolescent and lateral corners protruding forwards; eyes glabrous; fourth and fifth antennal segment 1.4–1.5 and 1.2–1.4 (♀) (of ♂ 1.4 and 1.4) times as long as third segment, respectively (♂ Fig. 151); apical antennal segment of ♀ 1.3 times as long as third antennal segment and its colour similar to colour of medial segments; antenna of female black or dark brown; mesoscutum and head with satin sheen, head dorsally very finely punctulate and mesoscutum coriaceous with spaced medium-sized punctures and some rugae medio-posteriorly, at most mesoscutum superficially rugulose; hind coxa transversely rugose dorsally, mainly coriaceous; hind tibia robust, with a distinct subbasal ivory ring and swollen, resulting in a distinctly convex ventral border (Figs 145, 150); hind basitarsus comparatively long (Figs 145, 150); hind tibial spurs brown; hind tarsus dark brown basitarsus (except basally) white; incision of hypopygium deep, slit-shaped.

#### Description. 

Holotype of *Gasteruption japonicum*, female, according to original description body length 20 mm (holotype has apical metasomal tergites and ovipositor missing, length up to apex of hypopygium about 18 mm).

*Head*. Vertex and frons with satin sheen and rather sparsely very finely punctulate, moderately convex and without a depression medio-posteriorly (Fig. 147); head gradually narrowed behind eyes; temple 0.5 times as long as eye in dorsal view (Fig. 147); fourth antennal segment 1.5 times as long as third segment and about as long as second and third segments combined, fifth antennal segment 1.3 times as long as third segment, third antennal segment 1.7 times as long as second segment; occipital carina narrow lamelliform medio-dorsally; OOL 1.2 times as long as diameter of posterior ocellus; face rather wide (Fig. 146); minimum width of malar space 0.2 times as long as second antennal segment (Fig. 142); clypeus with indistinct triangular depression, its lateral corners protruding forwards and medio-ventrally slightly emarginate; eye glabrous, but according to original description setose.

*Mesosoma*. Length of mesosoma twice its height; pronotal side moderately high and ventrally coriaceous and partly rugulose, without a distinct antero-lateral tooth; mesoscutum not protruding anteriorly; propleuron comparatively slender, 0.9 times as long as mesoscutum in front of tegulae (Fig. 143); antesternal carina narrow and narrowly lamelliform; mesoscutum densely coriaceous and with spaced medium-sized punctures and with satin sheen, rather matt, posteriorly with a few rugae (Fig. 144); scutellum entirely coriaceous.

*Wings*. Fore wing: first discal cell parallel-sided and with outer posterior corner rounded (Fig. 148), glabrous; vein SR1 slightly bent.

*Legs*. Hind coxa rather matt, slender, coriaceous and dorsally transversely rugose; length of hind femur, tibia and basitarsus 4.5, 5.0 and 7.6 times their width, respectively (Fig. 145); middle tarsus 1.3 times as long as middle tibia; middle femur subparallel-sided and slenderer than fore femur.

*Metasoma*. Ovipositor sheath missing but according to original description “longer than the body” (but according to measurements at the end of the description 0.95 times as long as body!) and sheath apically “broadly white”.

*Colour*. Black or black-brown; mandible, fore and middle femora narrowly apically, fore basitarsus and second-fourth metasomal tergites apically more or less brown, tegulae and remainder of legs largely dark brown, but hind tibial spurs brown and fore and middle tibiae hind tibia basally and apically, large subbasal patch of hind tibia (ventrally wider than dorsally) and hind basitarsus (but basally dark brown) white or ivory; pterostigma dark brown.

*Male* (described after a male from Zhejiang). Head behind eyes roundly narrowed in dorsal view (Fig. 152); occipital carina distinctly pigmented, narrow and non-lamelliform medio-dorsally; face wide; third antennal segment about 1.7 times as long as second segment (Fig. 151); fourth segment 1.4 times as long as third segment, as fifth segment, and 0.8 times as long as second and third segments combined; vertex coriaceous, matt; eye glabrous; frons rather convex and anterior ocellus above upper level of frons; propleuron slender and 0.9 times as long as mesoscutum in front of tegulae; antesternal carina non-lamelliform and narrow; mesoscutum densely coriaceous, mixed with fine transverse rugulosity and posteriorly distinctly rugose; hind coxa slender in dorsal view; hind basitarsus transversely rugose dorsally; hind leg coloured as in female but hind tarsus largely brown (Fig. 150); apical half of first metasomal tergite and second tergite black; paramere black apically; body length 9.7 mm. Very similar to female, but slightly more coarsely sculptured.

*Variation*. Body length 9.0–16.7 mm. Ovipositor sheath 0.9–1.1 times as long as body, 1.3–1.6 times as long as metasoma and 4.7–6.0 times as long as hind tibia; its white apical part 1.4–2.0 times as long as hind basitarsus; temple 0.5–0.6 times as long as eyes in dorsal view; third antennal segment 1.7–2.0 times as long as second segment, fourth antennal segment 1.4–1.5 times as long as third segment, fifth antennal segment 1.2–1.4 times as long as third segment; eye glabrous or shortly setose; propleuron 0.9–1.1 times as long as mesoscutum in front of tegulae; mesoscutum punctate-rugose and spaced medium-sized punctates; propodeum reticulate or reticulate-rugose, longitudinal carina indistinct or distinct; hind coxa with satin sheen and regularly transversely rugose or sometimes coriaceous.

#### Distribution.

China (Heilongjiang, Jilin, Inner Mongolia, Shaanxi, Ningxia, Gansu, Xinjiang, Shanghai, Zhejiang, Fujian, Taiwan, Hubei, Hunan, Sichuan, Yunnan); Japan (Honshu, Kokkaido).

#### Biology.

Unknown. Collected in March–December.

### Gasteruption latitibia sp. n.

urn:lsid:zoobank.org:act:D0D14A99-2C3B-46E7-9B79-487FCA5B877E

http://species-id.net/wiki/Gasteruption_latitibia

Figs 153 165

#### Type material.

Holotype, ♀ (SCAU). “[China:] Guizhou, Mayang River, 2.X.2007, Cui-hong Xie”. Paratypes. 1 ♀ (ZJUH), “[China:] Fujian, Shaxian, VII.1979, Zhen-yong Mei”; 1 ♀ (SCAU), “[China:] Hubei, Mt. Jing, 15.VII.2009, Yuan Ye”; 1 ♀ (ZJUH), “[China:] Hunan, Changsha, 27.VI.1982”; 1 ♂ (SCAU), “[China:] Hunan, Mt. Huping, Shinianzigou, 9.VII.2009, Ya-li Tang”; 1 ♂ (ZJUH), “[China:] Hunan, Liuyang, Xin-wang Tong”.

#### Diagnosis. 

**Figures 153–160.**
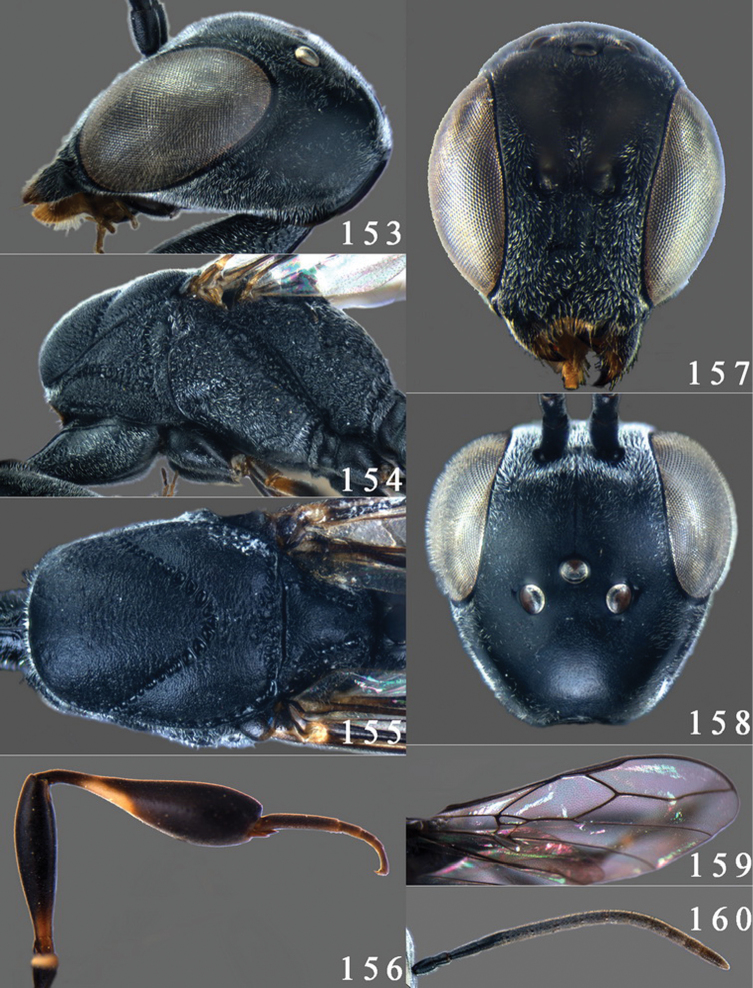
*Gasteruption latitibia* sp. n., holotype, female. **153** head lateral **154** mesosoma lateral **155** mesoscutum dorsal **156** hind leg **157** head anterior **158** head dorsal **159** fore wing **160** antenna.

**Figures 161–165.**
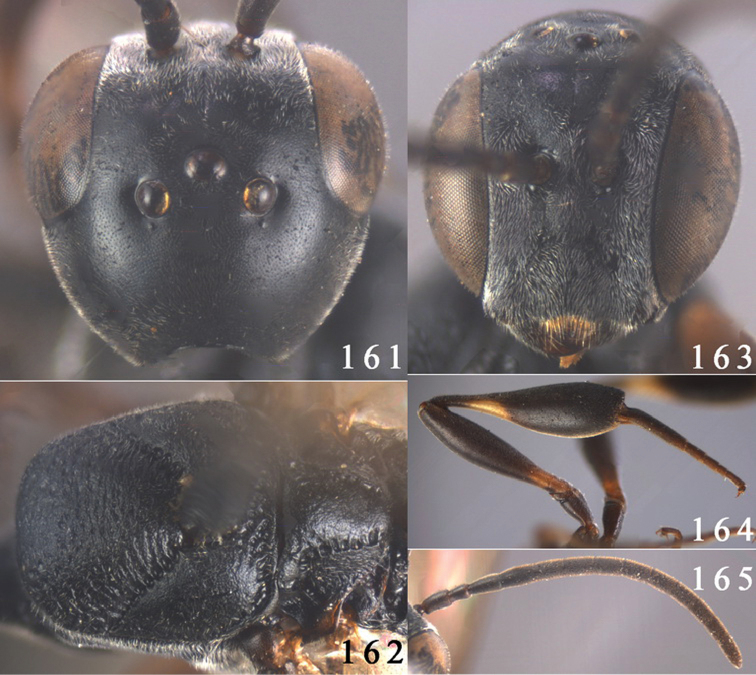
*Gasteruption latitibia* sp. n., paratype, male. **161** head dorsal **162** mesoscutum dorsal **163** head anterior **164** hind leg **165** antenna.

Hind tibia inflated (Figs 156, 164); side of pronotum with moderately wide to wide and distinctly crenulate grooves (Figs 155, 162); mesoscutum predominantly finely coriaceous, at most with some shallow punctures (Figs 155, 162); head more protruding in lateral and anterior view (Figs 153, 157, 163); vertex virtually smooth; vertex and frons more or less shiny and largely smooth (Figs 158, 161) or very finely punctulate; third antennal segment 1.7–1.9 times as long as second segment; propleuron rugulose antero-dorsally; hind basitarsus less slender (Figs 156, 164); propleuron 0.8–1.0 times as long as mesoscutum in front of tegulae moderately slender (Fig. 154); hind basitarsus comparatively short and black (Figs 156, 164); head truncate medio-posteriorly or nearly so; occipital carina obsolescent to narrowly lamelliform medio-dorsally (Fig. 153); malar space 0.1–0.3 times as long as second antennal segment (= pedicellus); median carina of propodeum absent (but sometimes a slightly elevated smooth median line), if present then surrounding reticulate-rugose and carina similarly developed; hind tibia about 1.2 times as long as hind femur and trochanter combined and less swollen (Figs 156, 164); ovipositor sheath 0.7–0.9 times as long as hind tibia and tarsus combined, at most 0.3–0.6 times as long as metasoma and 1.2–1.4 times as long as hind tibia; hypopygium comparatively shallowly emarginate posteriorly; apex of ovipositor sheath black or dark brown.

#### Description.

Holotype, female, body length 12.5 mm, of fore wing 6.0 mm.

*Head*. Head directly narrowed behind eyes and weakly curved laterally (Fig. 158); temple 0.8 times as long as eye in dorsal view; vertex and frons shiny and rather sparsely very finely punctulate; vertex moderately convex posteriorly and without depression medio-posteriorly; occipital carina narrow and non-lamelliform medio-dorsally (Fig. 153); third antennal segment 1.9 times as long as second segment, fourth antennal segment as long as third segment, fifth antennal segment 0.8 times as long as third segment (Fig. 160); eye setose; OOL 1.3 times as long as diameter of posterior ocellus; minimum width of malar space 0.3 times as long as second antennal segment; clypeus with a distinct angular depression (Fig. 157).

*Mesosoma*. Length of mesosoma 1.8 times as long as its height; propleuron robust, 0.8 times as long as mesoscutum in front of tegulae (Fig. 154); side of pronotum rugose, with a indistinct antero-lateral tooth; mesoscutum matt, with finely rugulose or coriaceous (Fig. 155); medio-posteriorly with finely punctate-rugose; scutellum coriaceous; propodeum reticulate, medio-longitudinal carina distinct.

*Wings*. Fore wing: first discal cell parallel-sided and with outer posterior corner rounded (Fig. 159).

*Legs*. Hind coxa matt, with satin sheen, comparatively slender and transversely rugose ; length of hind femur, tibia and basitarsus 3.8, 3.7 and 5.1 times as long as their width, respectively (Fig. 156); middle tarsus 1.3 times as long as middle tibia.

*Metasoma*. Ovipositor sheath 0.3 times as long as body, 0.4 times as long as metasoma, 1.3 times as long as hind tibia and about 0.8 times as long as hind tibia and tarsus combined; hypopygium v-shaped incised apically.

*Colour*. Black; mandible and antenna (except for four black basal segments) dark brown; fore and middle trochanters, femura and tibiae dark brown, tarsi brown, but middle basitarsus basally ivory; subbasal patch of hind tibia ivory; second-fifth metasomal tergites with red-brown band.

*Male* (described after a male from Hunan). Body length 13.0 mm; temple 0.7 times as long as eye in dorsal view (Fig. 161); third antennal segment 1.6 times as long as second segment, fourth antennal segment as long as third segment, fifth antennal segment 0.8 times as long as third segment (Fig. 165); minimum width of malar space 0.2 times as long as second antennal segment; length of mesosoma 1.7 times as long as its height; propleuron 0.7 times as long as mesoscutum in front of tegulae; mesoscutum sculpture more densely than of female (Fig. 162); length of hind femur, tibia and basitarsus 4.2, 3.7 and 4.6 times their width, respectively (Fig. 164); middle tarsus 1.1 times as long as middle tibia.

*Variation*. Female: body length 11.0–12.0 mm, ovipositor sheath 0.3–0.5 times as long as metasoma, 1.2–1.4 times as long as hind tibia and 0.7–0.9 times as long as hind tibia and tarsus combined. Temple 0.7–0.8 times as long as eye in dorsal view; third antennal segment 1.7–1.9 times as long as second segment, fourth antennal segment 0.9–1.0 times as long as third segment, fifth antennal segment 0.7–0.8 times as long as third segment; OOL 1.2–1.3 times as long as diameter of posterior ocellus; length of mesosoma 1.7–1.9 times as long as its height; propleuron 0.8–0.9 times as long as mesoscutum in front of tegulae, with a comparatively distinct antero-lateral tooth; mesoscutum matt and coriaceous, rather sparsely very finely punctate, or very finely rugulose; length of hind femur, tibia and basitarsus 3.8–4.5, 3.5–3.8 and 4.3–5.2 times as long as their width, respectively. Male: body length 11.7 mm. Temple 0.7–0.8 times as long as eyes in dorsal view; third antennal segment 1.6 times as long as second segment, fourth antennal segment 1.0–1.1 times as long as third segment, fifty antennal segment 0.8–0.9 times as long as third segment; OOL 1.1 times as long as diameter of posterior ocellus; length of mesosoma 1.7–1.8 times as long as its height; propleuron 0.9 times as long as mesoscutum in front of tegulae; length of femur, tibia and basitarsus 4.0, 3.7 and 4.7 times as long as their width, respectively.

#### Distribution.

China (Hubei, Hunan, Fujian, Guizhou).

#### Etymology.

Named after the widened (“latus” is Latin for “wide”) hind tibia.

#### Biology.

Unknown. Collected in June, July and October.

### Gasteruption oriplanum

Kieffer, 1911

http://species-id.net/wiki/Gasteruption_oriplanum

Figs 166–173

Gasteruption oriplanum Kieffer, 1911: 210; Hedicke 1939: 27.

#### Type material.

Holotype of *Gasteruption oriplanum*, ♂ (BMNH), “Type”, “B. M. Type Hym. 3.a.173”, “*Gasteruption oreiplanus* [sic!] Kieff.”, “[China:], Tibet, Gyangtse, 13,000 ft., June 1904, Tibet Exped., H.J. Walton, 1905-172/ 29.vi.1904”.

#### Additional material.

3 ♂ (ZJUH, RMNH), “[China:] Tibet, Zhenge, 3722 m, 11.VI.2009, Jiang-li Tan”; 1 ♀ (CSCS), “[China:] Tibet, Linzhi, Bomi, 2800 m, 20.VIII.2003, Wei Xiao”.

#### Diagnosis.

**Figures 166–173.**
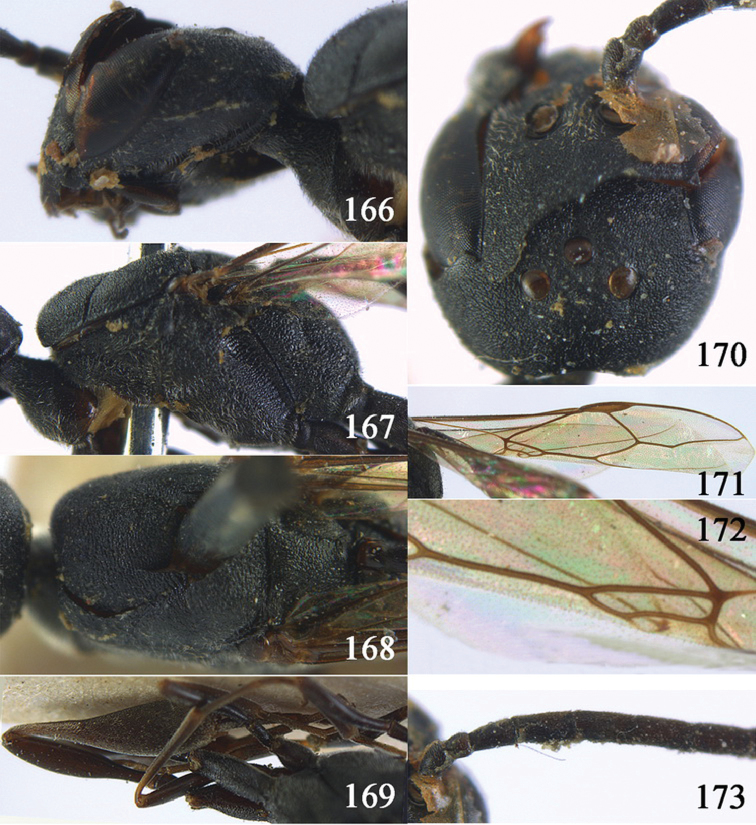
*Gasteruption oriplanum* Kieffer, 1911, holotype, male. **166** head lateral **167**  mesosoma lateral **168** mesoscutum dorsal **169** hind leg **170** head dorsal **171** fore wing **172** discal cell of fore wing **173** the first to seventh antennal segments.

Apex of ovipositor sheath black; ovipositor sheath 1.1 times as long as hind tibia and 0.7 times as long as hind tibia and tarsus combined; occipital carina obsolescent medio-dorsally (Fig. 166) and somewhat protruding ventro-posteriorly (Fig. 166); antesternal carina narrow; head, laterally mesosoma and scape black; head in anterior view distinctly protruding below lower level of eyes by 0.7 times basal width of mandible and mandibular condylus below lower level of eyes; in lateral viewcondylarincision of malar space distinctly removed from eye (Fig. 166); outer side of mandible distinctly convex; clypeus with obsolescent depression; eyes glabrous; fourth and fifth antennal segments of ♂ 1.3 and 1.3 (♀: 1.2 and 1.0) times as long as third segment, respectively (Fig. 173); apical antennal segment of HT missing, at most 1.4 times as long as third antennal segment and its colour similar to colour of medial segments; antenna of female black; mesoscutum and head similarly coriaceous, at most mesoscutum superficially rugulose; propleuron robust and about 0.6–0.7 times as long as mesoscutum in front of tegulae (Fig. 167); hind coxa mainly rugulose dorsally; hind tibia robust, without a subbasal ivory ring and swollen, resulting in a distinctly convex ventral border (Fig. 169); hind basitarsus comparatively long and parallel-sided (Fig. 169); hind tibial spurs brown; hind tarsus dark brown.

#### Description.

Holotype, male, body length 9.3 mm.

*Head*. Vertex and frons matt and very densely finely coriaceous, moderately convex and without a depression medio-posteriorly; head gradually narrowed behind eyes (Fig. 170); temple 0.7 times as long as eye in dorsal view; fourth antennal segment 1.3 times as long as third segment and 0.8 times as long as second and third segments combined, fifth antennal segment 1.3 times as long as third segment (Fig. 173), third antennal segment 1.7 times as long as second segment; occipital carina narrow and non-lamelliform medio-dorsally; ocelli comparatively small, OOL 1.4 times as long as diameter of posterior ocellus; face rather wide; minimum width of malar space as long as second antennal segment (Fig. 166; incorrectly described in original description); clypeus without triangular depression and slightly emarginate; eye glabrous.

*Mesosoma*. Length of mesosoma 1.7 times its height; pronotal side moderately high and coriaceous except for medial and subposterior crenulate grooves, without antero-lateral tooth; mesoscutum slightly protruding anteriorly; propleuron robust, 0.7 times as long as mesoscutum in front of tegulae (Fig. 167); antesternal carina narrow and hardly lamelliform; mesoscutum densely coriaceous and rather matt, posteriorly with some rugulae (Fig. 168); scutellum coriaceous; propodeum rugulose and with median carina, distinctly more developed then surrounding sculpture.

*Wings*. First discal cell slightly sinuate posteriorly and with outer posterior corner rounded (Figs 171, 172).

*Legs*. Hind coxa rather matt, slender, coriaceous; length of hind femur, tibia and basitarsus 4.2, 3.9 and 6.8 times their width, respectively (Fig. 169); middle tarsus normal, 1.1 times as long as middle tibia; middle femur narrowed basally and slenderer than fore femur.

*Metasoma*. Paramere normal and completely black.

*Colour*. Black or black-brown; hind tibial spurs brown; hind tibia with large ventral subbasal patch ivory, but brown basally; pterostigma brown.

*Female*. Head behind eyes roundly narrowed in dorsal view; occipital carina distinctly pigmented, narrow and non-lamelliform medio-dorsally; face wide; third antennal segment about 1.4 times as long as second segment; fourth segment 1.2 times as long as third segment, as fifth segment, and 0.7 times as long as second and third segments combined; vertex coriaceous, matt; eye glabrous; frons rather convex and anterior ocellus above upper level of frons; propleuron robust and 0.7 times as long as mesoscutum in front of tegulae; antesternal carina non-lamelliform and narrow; mesoscutum densely coriaceous, mixed with fine transverse rugulose and posteriorly distinctly rugose; hind coxa slender in dorsal view; hind basitarsus transversely rugose dorsally; hind leg coloured as in female but hind tarsus largely brown; apical half of first metasomal tergite and second tergite black; paramere black apically; body length 9.0 mm; ovipositor sheath 0.3 times as long as body, 0.4 times as long as metasoma and 1.1 times as long as hind tibia; sheath apically dark brown; hypopygium v-shaped incised apically.

*Variation*. Male: body length 10.0–12.5 mm; head deeply emarginated medio-posteriorly; temple as long as eye in dorsal view; vertex and frons finely aciculate; third antennal segment 1.5–1.6 times as long as second segment, fourth antennal segment 1.4 times as long as third segment, fifth antennal segment 1.2–1.3 times as long as third segment; OOL 1.8 times as long as diameter of posterior ocellus; minimum width of malar space as long as second antennal segment; length of mesosoma 1.7–1.8 times as long as its height; propleuron coarsely rugose and robust, about 0.6 times as long as mesoscutum in front of tegulae; dorsal lobe of pronotum rugose or rugulose, ventrally densely rugose, ventro-laterally coarsely rugose, dorso-laterally coriaceous; mesoscutum matt and finely densely transversely rugulose, posteriorly weakly irregularly rugulose; propodeum rugose, medio-longitudinal carina distinct; first discal cell broaden; hind coxa matt, slender and dorsally transversely rugose; length of hind femur, tibia and basitarsus 4.5–4.8, 3.9–4.2 and 5.6–6.5 times their width, respectively; middle tarsus 1.2–1.3 times as long as middle tibia.

#### Distribution.

China (Tibet, 2800–4300 m).

#### Biology. 

Unknown. Collected in June and August.

#### Notes. 

Very similar to *Gasteruption minutum* (Tournier), but this species has the first discal cell straight posteriorly and hind tarsus yellow-brown.

### Gasteruption parvicollarium

Enderlein, 1913

http://species-id.net/wiki/Gasteruption_parvicollarium

Figs 174 190

Gasteruption parvicollarium Enderlein, 1913: 323–324; Hedicke 1939: 27; Pasteels 1958: 183, fig. 8.

#### Type material.

Holotype, ♂ (DEI), “[China:] Formosa [= Taiwan], Kankau (Koshun) VII.1911, H. Sauter”, “Holotypus”, “*Gasteruption parvicollarium* Enderl., ♂, Type, Dr. Enderlein, det. 1913”, “Dtsch. Entomol. Institut Berlin”.

#### Additional material.

1 ♀ (ZJUH), “[China:] Jiangsu, Yixing, 4.VI.1935”; 1 ♀ (ZJUH), “[China:] Zhejiang, West Mt. Tianmu, 17–18.V.1988, Xiao-ming Lou”; 1 ♀ (ZJUH), “[China:] Zhejiang, West Mt. Tianmu, 1–3.VII.2010, Qi-chao Zhao”; 1 ♂ (CAU): “[China:] Fujian, Chong'an, Mt. Xiaowuyi, 30.VI.1979, Ji-kun Yang”; 1 ♀ (RMNH), “[China:] Fujian, Fuzhou, 25.IV.1991, Nai-quan Lin”; 1 ♂ (ZJUH), “[China:] Fujian, Fuzhou, 17.IV.1991, Chang-ming Liu”; 1 ♀ (ZJUH), “[China:] Fujian, Mt. Wuyi, Dazhulan, 31.VII.1983, Jun-hua He”; 1 ♂ (ZJUH), “[China:] Fujian, Mt. Wuyi, 14.VII.1994”; 1 ♀ (SCAU), “[China:] Fujian, Jianyang, Masha, 28.IV.2012, Jun-hao Huang”; 5 ♀ + 1 ♂ (TARI), “[China:] Taiwan, Musha, 25.VI.–5.VII.1947, Maa, Chen & Lin”; 1 ♀ + 1 ♂ (TARI), “[China:] Taiwan, Makazayazaya, Heito, 9.X.1926, J. Sonan”; 1 ♂ “[China:] Hopeh [= Hebei] Prov., Sienhsien [= Xianxian], Musée Heude”; 1 ♀ (TARI), “[China:] Taiwan, Pingtung, Kenting, 22–26.III.1982, T. Lin & S.C. Lin”; 1 ♀ + 3 ♂ (TARI, RMNH), “[China:] Taiwan, Nantou, Lushan, 100 m, 27–31.V.1980, K.S. Lin & L.Y. Chou”; 6 ♂ (TARI, RMNH), “[China:] Taiwan, Nantou, Tungpu, 1200 m, 23–27.VII.1984, K.C. Chou & C.H. Yang”; 2 ♂ (TARI), “[China:] Taiwan: Nantou, Wushe, 1150 m, 7.V.1984, K.C. Chou & C.C. Pan”; 1 ♂ (TARI), id., but 17.VIII.1984; 2 ♂ (TARI), id., but 23–28.VI.1981, K.S. Lin & W.S. Tang; 1 ♂ (TARI), “[China:] Taiwan, Taitung, Chihpen, 17–18.II.1982, L.Y. Chou & K.C. Chou”; 1 ♀ (TARI), “[China:] Taiwan, Taipei, 2.XII.1968, K.H. Chang”; 1 ♂ (TARI), id., but 13.IX.1968; 1 ♀ (TARI), “[China:] Taiwan, Kuandouchi, 16–22.IV.1971, Malaise trap”; 1 ♂ (TARI), “[China:] Taiwan, KAGI, 15.III.1936, S. Toyota”; 1 ♂ (TARI), “[China:] Taiwan, Kotosho, 1–4.IV.1920, J. Sonan”; 1 ♀ (SCAU), “[China:] Ningxia, Mt. Liupan, 3–14.VII.2009, Hua-yan Chen”; 1 ♀ (SCAU), “[China:] Shanxi, Mt. Li National Nature Reserve, 26–30.VII.2012, Zhen Liu”; 1 ♀ (ZJUH), “[China:] Hubei, Wufenghouhe Nature Reserve, 11.VII.1999, Wen-jun Pu”; 2 ♀ + 1 ♂ (ZJUH), “[China:] Hunan, Liuyang, 6.V.1984 & 22.IX.1984 & 29.IV.1985, Xin-wang Tong”; 1 ♂ (ZJUH), “[China:] Guangxi, Guilin, 22.IX.1987, Jun-hua He”; 1 ♀ (ZJUH), “[China:] Guizhou, Meitan, V.1943, Ru-zuo Zhu”.

#### Diagnosis.

**Figures 174–181.**
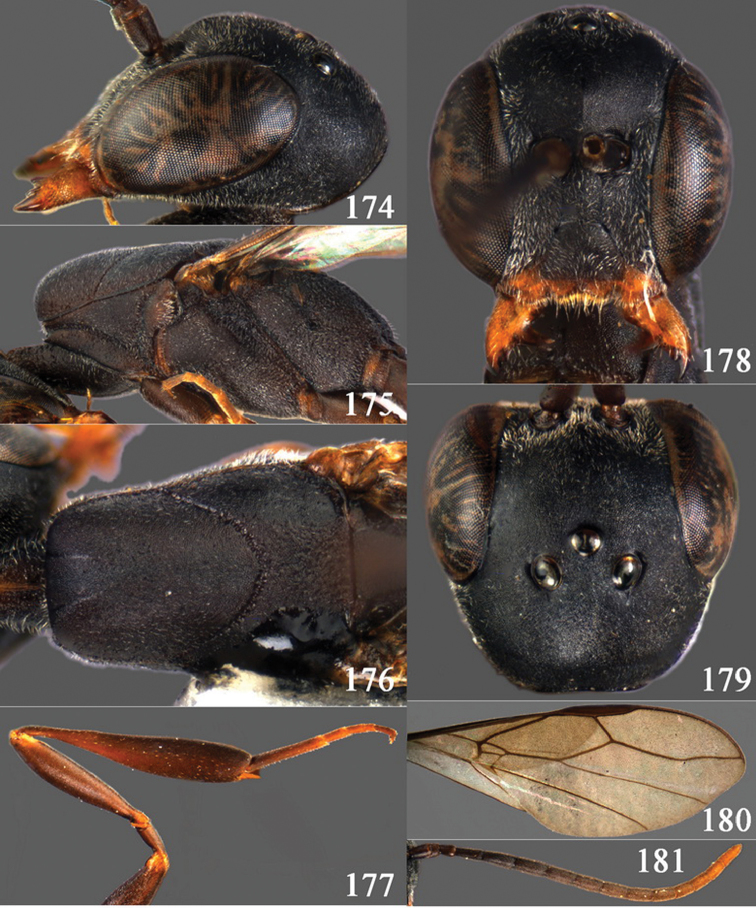
*Gasteruption parvicollarium* Enderlein, 1913, female, Zhejiang. **174** head lateral **175** mesosoma lateral **176** mesoscutum dorsal **177** hind leg **178** head anterior **179** head dorsal **180** fore wing **181** antenna.

**Figures 182–190.**
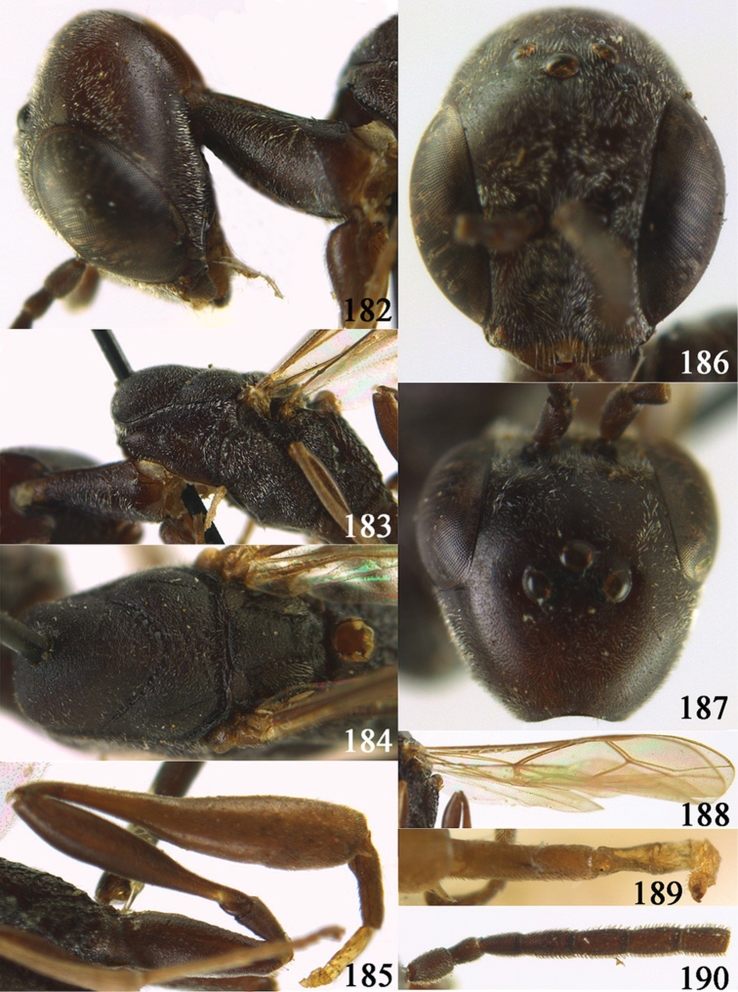
*Gasteruption parvicollarium* Enderlein, 1913, holotype, male. **182** head lateral **183** mesosoma lateral **184** mesoscutum dorsal **185** hind leg **186** head anterior **187** head dorsal **188** fore wing **189** tarsus of hind leg **190** the first to seventh antennal segments.

Apex of ovipositor sheath black or dark brown; ovipositor sheath 0.7–1.0 times as long as hind tibia and tarsus combined, at most 0.6 times as long as metasoma and 1.2–1.7 times as long as hind tibia; head nearly “fez-shaped” in anterior view (Figs 178, 186) and conical elongate in lateral view (Figs 174, 182); first discal cell of fore wing subparallel-sided (Figs 180, 188); occipital carina narrow and non-lamelliform medio-dorsally (Figs 174, 182); antesternal carina narrow; hind tibia moderately robust (Figs 177, 185); hind basitarsus comparatively short and black; malar space 0.1 times as long as second antennal segment (= pedicellus); propodeum regularly coarsely reticulate or scrobiculate, median carina of propodeum as a slightly elevated smooth median line; eyes glabrous; mesoscutum very finely and densely coriaceous, matt and with more or less obsolescent isolated punctures (Figs 176, 184); pronotum with only angulate antero-laterally; vertex and frons with satin sheen and very finely punctulate; propleuron 0.8–1.0 times as long as mesoscutum in front of tegulae and moderately slender (Figs 175, 183); notauli very shallow and narrow in ♀ but wider and more or less crenulate in ♂ (Figs 176, 184); hind tibia entirely brown (♂ or with pale patch (♀); apical 0.1 of hypopygium of ♀ incised.

#### Description.

Holotype, male, body length 11 mm, of fore wing 4.9 mm.

*Head*. Vertex and frons with satin sheen and very densely finely punctulate (somewhat coriaceous), moderately convex and without a depression medio-posteriorly; head gradually narrowed behind eyes; temple as long as eye in dorsal view (Fig. 187); fourth antennal segment 1.4 times as long as third segment and 0.8 times as long as second and third segments combined, fifth antennal segment 1.4 times as long as third segment (Fig. 190), third antennal segment 1.7 times as long as second segment and moderately slender (Fig. 190); occipital carina narrow and non-lamelliform medio-dorsally (Fig. 182); ocelli comparatively small, OOL 1.6 times as long as diameter of posterior ocellus; face moderately wide (Fig. 186); minimum width of malar space 0.1 times as long as second antennal segment (Fig. 182); clypeus without triangular depression and slightly emarginate; eye glabrous.

*Mesosoma*. Length of mesosoma 1.8 times its height; pronotal side moderately low and coriaceous except for medial and subposterior crenulate grooves, antero-laterally only angularly protruding; mesoscutum slightly protruding anteriorly; propleuron comparatively slender (Fig. 183), as long as mesoscutum in front of tegulae; antesternal carina narrow and hardly lamelliform; mesoscutum very finely and densely coriaceous, matt and with more or less obsolescent isolated punctures (Fig. 184); scutellum coriaceous; propodeum regularly coarsely reticulate or scrobiculate, median carina of propodeum as a slightly elevated smooth median line.

*Wings*. First discal cell parallel-sided and with outer posterior corner rounded (Fig. 188).

*Legs*. Hind coxa rather matt, slender, coriaceous; length of hind femur, tibia and basitarsus 4.8, 5.0 and 4.4 times their width, respectively (Fig. 185), middle tarsus 1.1 times as long as middle tibia; middle femur as slender as fore femur; apical segments of hind tarsus deformed (Fig. 189).

*Metasoma*. Paramere dark brown.

*Colour*. Black or black-brown; antenna, mandible, tegulae and legs (including hind tibial spurs) dark brown or nearly so; wing membrane subhyaline; pterostigma brown.

*Female* (described after a female from Zhejiang). Body length 13 mm; head truncate medio-posteriorly, gradually narrowed behind eyes and weakly curved laterally (Fig. 179); temple 0.7 times as long as eye in dorsal view; vertex and frons with satin sheen and coriaceous; vertex moderately convex posteriorly and without depression medio-posteriorly; occipital carina non-lamelliform and obsolescent medio-dorsally (Fig. 174); third antennal segment 1.4 times as long as second segment, fourth antennal segment 1.3 times as long as third segment, fifth antennal segment as long as third segment (Fig. 181); eye glabrous; OOL 1.4 times as long as diameter of posterior ocellus; minimum width of malar space 0.1 times as long as second antennal segment; clypeus with indistinct triangular depression (Fig. 178); length of mesosoma 2.3 times as long as its height; propleuron matt and moderately robust, 0.9 times as long as mesoscutum in front of tegulae (Fig. 175); side of pronotum coriaceous, without a distinct antero-lateral tooth; mesoscutum coriaceous and matt (Fig. 176); medio-posteriorly weakly rugulose; scutellum coriaceous and matt; propodeum reticulate-rugose, medio-longitudinal carina distinct; hind coxa matt, slender and dorsally regularly weakly rugulose; length of hind femur, tibia and basitarsus 3.7, 4.5 and 5.6 times their width, respectively (Fig. 177); middle tarsus 1.2 times as long as middle tibia; ovipositor sheath 0.2 times as long as body, 0.3 times as long as metasoma, 0.8 times as long as hind tibia and tarsus combined and 1.3 times as long as hind tibia; hypopygium shallow v-shaped incised apically.

*Variation*. Male: body length 10.0–12.0 mm; third antennal segment 1.2–1.3 times as long as second segment, fourth antennal segment 1.2–1.3 times as long as third segment, fifth antennal segment 1.1–1.2 times as long as third segment. Female: body length 10.0–13.0 mm, ovipositor sheath 0.2–0.3 times as long as body, 0.3–0.6 times as long as metasoma, 0.7–1.0 times as long as hind tibia and tarsus combined and 1.2–1.7 times as long as hind tibia. Temple 0.5–0.8 times as long as eye in dorsal view; third antennal segment 1.2–1.6 times as long as second segment, fourth antennal segment 1.1–1.2 times as long as third segment, fifth antennal segment 1.0–1.2 times as long as third segment; OOL 1.4–1.6 times as long as diameter of posterior ocellus; length of mesosoma 2.3–2.6 times as long as its height; propleuron 0.8–0.9 times as long as mesoscutum in front of tegulae.

#### Distribution.

China (Ningxia, Shanxi, Hebei, Jiangsu, Zhejiang, Fujian, Taiwan, Hubei, Hunan, Guangxi, Guizhou).

#### Biology.

Unknown. Mainly collected in February–October and December.

### Gasteruption poecilothecum

Kieffer, 1911

http://species-id.net/wiki/Gasteruption_poecilothecum

Figs 191–199

Gasteruption poecilothecus Kieffer, 1911: 205.

#### Type material.

Holotype, ♀ (BMNH), “Type”, B.M. Type 3.a.164”, “*Gasteruption poecilothecus* Kieff.”, “[Far East Russia or North China], Amoor [= Amur River= Heilongjiang] / 71 25”, “Determined by Dr. Kieffer”.

#### Additional material.

1 ♀ (ZJUH), “[China:] Jilin, Jiao River, VII.1988”; 1 ♀ (RMNH), “[China:] Jilin, Daxinggou, 7.VIII.2005, Mao-ling Sheng”; 1 ♀ (ZJUH), “[China:] Hebei, Pingquan, 3.VII.1986, Jin-jun Du”; 1 ♀ (CSCS), “[China:] Xinjiang, Aletai, Kanasi, 16.VII.2007, N48°40.056’, E87°02.150’, alt. 1386 m, Mei-cai Wei”.

#### Diagnosis.

**Figures 191–199.**
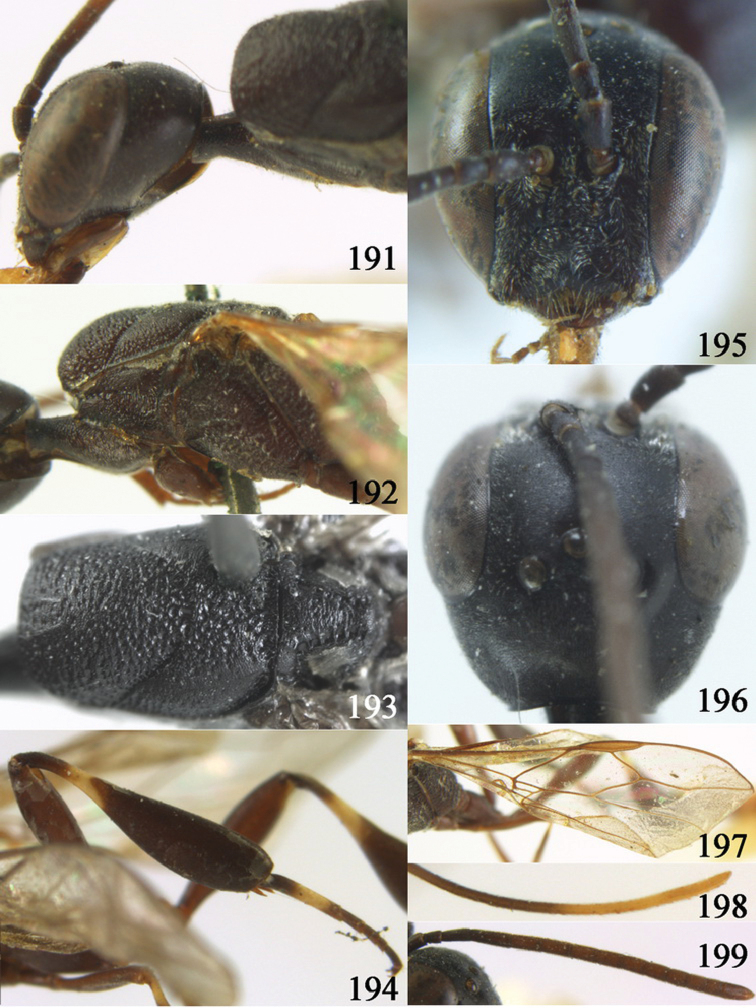
*Gasteruption poecilothecum* Kieffer, 1911, holotype, female. **191** head lateral **192** mesosoma lateral **193** mesoscutum dorsal **194** hind leg **195** head anterior **196** head dorsal **197** fore wing **198** apex of ovipositor sheath **199** antenna.

Ivory apex of ovipositor sheath (Fig. 198) 1.4-1.9 times as long as hind basitarsus; ovipositor sheath about as long as body; occipital carina narrow lamelliform medio-dorsally (Fig. 191) and rather protruding laterally (Fig. 191); propleuron robust, 0.8 times as long as mesoscutum in front of tegulae (Fig. 192); antesternal carina narrow; head, laterally mesosoma and scape black-brown; head in anterior view not protruding below lower level of eyes and mandibular condylus near lower level of eyes (Fig. 195); in lateral viewcondylarincision of malar space close to eye (Fig. 191); clypeal ventral depression obsolescent and lateral corners rather protruding forwards; eyes glabrous; fourth and fifth antennal segment 1.4 and 1.1 (♀) times as long as third segment, respectively (Fig. 199); apical antennal segment of ♀ 1.4 times as long as third antennal segment and brown, as colour of middle segments; antenna of female brown, except dark brown basal quarter; mesoscutum and head with satin sheen, head dorsally very finely punctulate and mesoscutum coriaceous between large and many punctures, become punctate-rugose medio-posteriorly; hind coxa transversely rugose dorsally, interspaces mainly rugulose; hind tibia robust, with a distinct subbasal ivory ring and swollen, resulting in a distinctly convex ventral border (Fig. 194); hind basitarsus comparatively long and medially ivory (Fig. 194); hind tibial spurs brown; remainder of hind tarsus dark brown; incision of hypopygium deep.

#### Description. 

Holotype, female, body length 14 mm.

*Head*. Vertex and frons with satin sheen and densely and very finely punctulate or subcoriaceous, flat medio-posteriorly; head gradually narrowed behind eyes (Fig. 196); temple 0.5 times as long as eye in dorsal view; fourth antennal segment 1.4 times as long as third segment and 0.9 times as long as second and third segments combined, fifth antennal segment 1.1 times as long as third segment (Fig. 199), third antennal segment 1.9 times as long as second segment; occipital carina narrow lamelliform medio-dorsally (Fig. 191); OOL 1.1 times as long as diameter of posterior ocellus; face rather wide (Fig. 195); minimum width of malar space 0.2 times as long as second antennal segment (Fig. 191); clypeus without distinct depression, its lateral corners protruding forwards and medio-ventrally slightly emarginate; eye glabrous.

*Mesosoma*. Length of mesosoma 1.8 times its height; pronotal side moderately high and ventrally coriaceous and partly rugulose, with a distinct antero-lateral tooth; mesoscutum not protruding anteriorly; propleuron robust, 0.8 times as long as mesoscutum in front of tegulae (Fig. 192); antesternal carina narrow and narrowly lamelliform; mesoscutum densely coriaceous and with many large punctures and with satin sheen, rather matt, medio-posteriorly punctate-rugose (Fig. 193); scutellum partly finely punctate and with some transverse rugae.

*Wings*. Fore wing: first discal cell parallel-sided and with outer posterior corner rounded (Fig. 197), glabrous; vein SR1 slightly bent.

*Legs*. Hind coxa with satin sheen, slender, coriaceous and dorsally transversely rugose; length of hind femur, tibia and basitarsus 4.1, 4.1 and 6.6 times their width, respectively (Fig. 194); middle tarsus 1.2 times as long as middle tibia; middle femur subparallel-sided and slenderer than fore femur.

*Metasoma*. Ovipositor sheath 5.0 times as long as hind tibia, 1.6 times metasoma and 1.1 times body; ivory part of sheath 1.5 times as long as hind basitarsus; hypopygium deep slit-shaped incised apically.

*Colour*. Black-brown; antenna (except dark basal quarter) brown; fore leg and middle tibia and tarsus brown, but tibiae ivory basally; second-fourth metasomal tergites apically more or less yellow-brown, tegulae and remainder of legs largely dark brown, but hind tibial spurs brown and a large subbasal patch of hind tibia (ventrally wider than dorsally) and hind basitarsus (but basally and apically dark brown) white or ivory; pterostigma brown.

*Male*. Unknown.

*Variation*. Body length 14.5–16.0 mm. Ovipositor sheath 1.0–1.1 times as long as body, 1.6–1.7 times as long as metasoma and 4.7–5.1 times as long as hind tibia; its white apical part 1.4–1.9 times as long as hind basitarsus.

#### Distribution.

China (Heilongjiang, Jilin, Xinjiang, Hebei).

#### Biology.

Unknown. Collected in July and August.

#### Notes.

The sculpture of the mesoscutum is variable in this species; it varies from some shallow punctures up to many (but well separated) coarse punctures (Fig. 193). Also the colour of the hind basitarsus is variable (as in most species; often largely dark brown or black but sometimes with distinct ivory part).

### Gasteruption rufescenticorne

Enderlein, 1913

http://species-id.net/wiki/Gasteruption_rufescenticorne

Figs 200 216

Gasteruption rufescenticorne Enderlein, 1913: 324–325; Hedicke 1939: 28; Pasteels 1958: 177, fig. 2.

#### Type material.

Lectotypehere designated, ♀ (ZMIZ), “Type”, “[China:] Formosa [= Taiwan], Fuhosho, III.[19]09, H. Sauter”, “*Gasteruption rufescenticorne* Enderl., ♀, Type, Dr. Enderlein, det. 1913”, “Mus. Zool. Polonicum, Warszawa, 12/45”, “MIZ 313248”, “Mus. Zool. Polomocum Warszawa, Typus n. 1522, *Gasteruption rufescenticorne* Enderlein, 1913, Syntypus”. Paralectotype, ♂ (according to label; metasoma and antenna missing; DEI), “[China:] Formosa [= Taiwan], Takao, 5.VII.[19]07, H. Sauter S.”, “Syntypus”, “*Gasteruption rufescenticorne* Enderl., ♂, Type, Dr. Enderlein det. 1913”, “Dtsch. Entomol. Institut Berlin”. Additional paralecotype (1 ♂ from Takao) in ZMIZ.

#### Additional material.

1 ♀ (TARI), “ZÔ-SE”, “Chine, Prov. Kiangsu [= Jiangsu], Shanghai, Musée Heude”, “10.VI.[19]30, O. Piel”; 1 ♀ (TARI), “[China:] Chenkiang [= Zhenjiang], Chusan, Musée Heude”, “4.VI.[19]31”, “O. Piel”; 1 ♀ + 2 ♂ (ZJUH), “[China:] Zhejiang, Hangzhou, 4.VII.1980”; 1 ♀ (ZJUH), “[China:] Zhejiang, Huizhou, Shishi, 25.IX.1984, Cai-e Zhou”; 1 ♀ (SCAU), “[China:] Fujian, Fuzhou, 5.V.1965, Liang-chen Wang”; 1 ♂ (ZJUH), “[China:] Fujian, Fuzhou, Jinshan, 29.IV–2.V.1990, Xiu-fu Zhao”; 2 ♂ (ZJUH), “[China:] Fujian, Fuzhou, Jinshan, 6.VI.1989; 10.VI.1989, Xiu-fu Zhao”; 1 ♂ (ZJUH), “[China:] Fujian, Fuzhou, Jinshan, 3–9.V.1990, Xiu-fu Zhao”; 1 ♂ (ZJUH), “[China:] Fujian, Fuzhou, Jinshan, 20.VI.1990, Xiu-fu Zhao”; 1 ♂ (ZJUH), “[China:] Fujian, Fuzhou, Jinshan, 14–18.X.1990, Xiu-fu Zhao”; 3 ♂ (ZJUH), “[China:] Fujian, Fuzhou, 17.VIII.1990, Chang-ming Liu”; 2 ♂ (ZJUH, RMNH), “[China:] Fujian, Fuzhou, 15.VI.1988, Sa-ping Yi”; 1 ♂ (ZJUH), “[China:] Fujian, Jiangle, Mt. Longqi, 1.VII.1991, Chang-ming Liu”; 1 ♂ (ZJUH), “[China:] Fujian, Shaxian, Yangfang, 5.VIII.1974, Xiu-fu Zhao”; 1 ♂ (NMNS) “Fujian, Kingmen, 15.IV.1999, by hand, M.M. Yang”; 1 ♂ (id.), “[China:] Taiwan, Jonghua, 11.IV.1971, B. S. Chang”; 1 ♀ (TARI), “[China:] Taiwan, Urai, VI.1931, J. Sonan”, 1 ♂ (TARI), “[China:] Taiwan, Renaechi, 17.IX.1933, J. Sonan”; 2 ♂ (TARI), “[China:] Taiwan, Kuandouchi, 22–28.VI.1971, Malaise trap”; 1 ♀ (TARI), id., but 25–31.V.1971; 1 ♂ (TARI), id., but 29.VI.–5.VII.1971; 1 ♂ (TARI), “[China:] Taiwan, Taichung, Puli, 16–19.V.1956”, K.S. Lin”; 1 ♂ “[China:] Taiwan, Nantou, Wushe, 1150 m, 7–8.X.1982, K.C. Chou”; 1 ♂ (SCAU), “[China:] Ningxia, Mt. Liupan, 3–14.VII.2009, Hua-yan Chen”; 1 ♀ + 1 ♂ “[China:] Hubei, Tongcheng, 8.VI.1979”; 1 ♀ + 1 ♂ (ZJUH), “[China:] Hunan, Liuyang, 30.V.1984, Xin-wang Tong”; 1 ♀ (ZJUH), “[China:] Hunan, Liuyang, 20.VI.1985, Xin-wang Tong”; 1 ♀ (ZJUH), “[China:] Hunan, Liuyang, 25.V.1986, Xin-wang Tong”; 1 ♂ (ZJUH), “[China:] Hunan, Liuyang, 13.V.1985, Xin-wang Tong”; 1 ♂ (ZJUH), “[China:] Hunan, Liuyang, 21.IX.1984, Xin-wang Tong”; 1 ♀ (CAU): “[China:] Guangxi, Guilin, Mt. Qixing, 28.IV.1963, Ji-kun Yang”; 1 ♂ (ZJUH), “[China:] Guangxi, Binzhou, 8.VI.1982, Jun-hua He”; 1 ♀ (SCAU), “[China:] Hainan, Mt. Diaoluo, 29.V.2007, Bin Xiao”.

#### Diagnosis.

**Figures 200–208.**
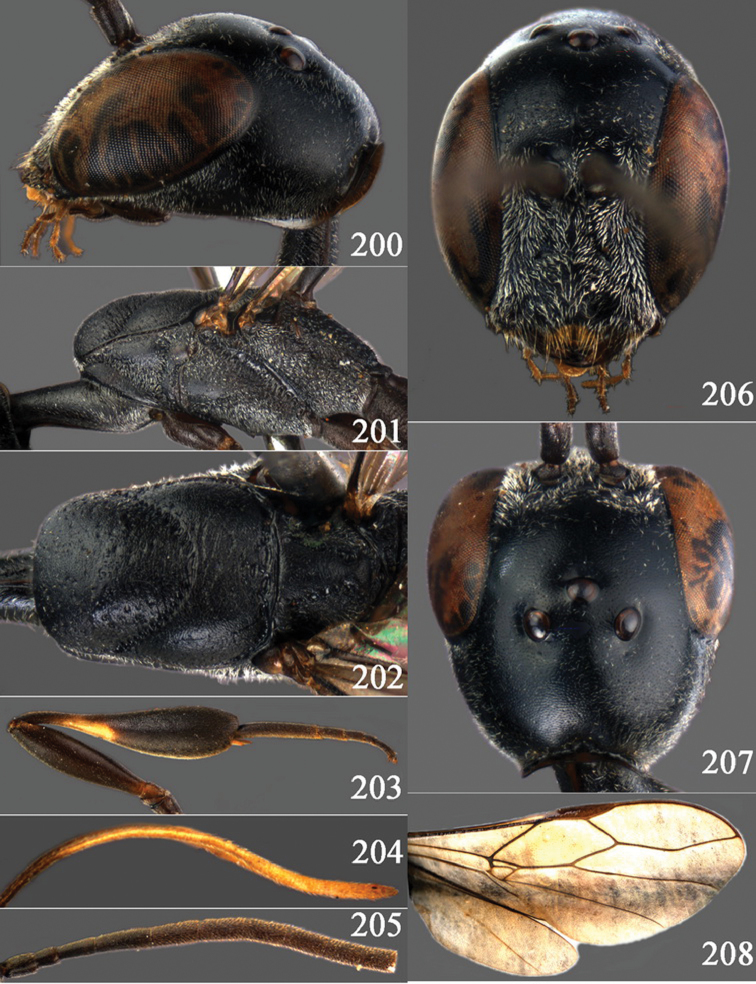
*Gasteruption rufescenticorne* Enderlein, 1913, female, Hunan. **200** head lateral **201** mesosoma lateral **202** mesoscutum dorsal **203** hind leg **204** apical of ovipositor sheath **205** the first to ninth antennal segments **206** head anterior **207** head dorsal **208** fore and hind wings.

**Figures 209–216.**
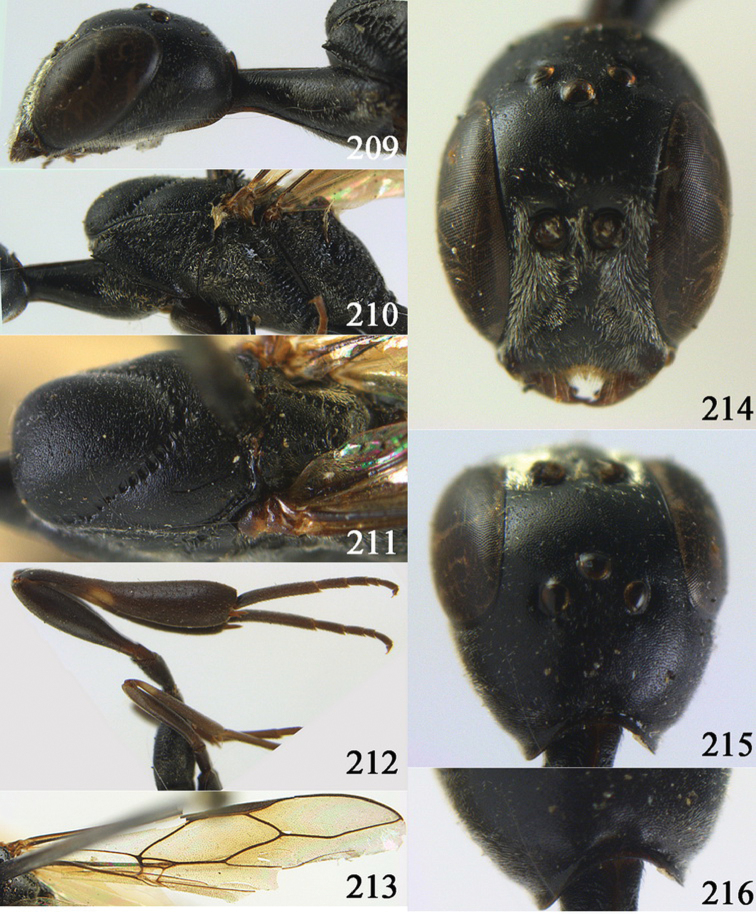
*Gasteruption rufescenticorne* Enderlein, 1913, holotype, male. **209** head lateral **210** mesosoma lateral **211** mesoscutum dorsal **212** hind legs **213** fore wing **214** head anterior **215** head dorsal **216** occipital carina.

Apical fifth of ovipositor sheath white (Fig. 204), 1–2 times as long as hind basitarsus; head distinctly excised posteriorly (♂ Fig. 215) or truncate (♀ Fig. 207), with a small triangular depression in front of wide to medium-sized lamelliform occipital carina medio-dorsally, with indistinct minute tubercles and deep cleft (Figs 200, 209); antesternal carina non-lamelliform and narrow (Fig. 201); propleuron 1.0–1.1 times as long as mesoscutum in front of tegulae and rather slender (Fig. 201); head roundly narrowed behind eyes in dorsal view (Figs 207, 215); temple much shorter than eye in dorsal view; antennal segments unknown of types, according to original description third segment about 1.3–1.6 times as long as second segment; malar space partly absent and condylus nearly touching eye; frons and vertex finely densely punctulate and with distinct satin sheen; antero-lateral teeth of pronotum absent, only somewhat angulate; mesoscutum coriaceous and with satin sheen, with minute separate punctures and medio-posteriorly mainly coriaceous with some large oval and round punctures (Figs 202, 211); marginal cell of fore wing elongate; hind coxa moderately slender and largely coriaceous, but dorsally transversely rugose; ovipositor sheath about 0.8–1.1 times as long as body and about 1.3–1.6 times as long as metasoma; hind coxa and pronotal side black; hind basitarsus entirely dark brown and slender, rarely with ivory patch; hind tibia slender and bicoloured (dorsally dark brown, ventrally with rather small ivory subbasal patch).

#### Description.

Syntype, male, length of head and mesosoma (metasoma missing) 6.4 mm.

*Head*. Vertex excised medio-posteriorly, with satin sheen and densely finely punctulate, moderately convex and with small triangular depression medio-posteriorly, with pair of minute tubercles (Figs 215, 216); head gradually narrowed behind eyes; temple 0.7 times as long as eye in dorsal view; third antennal segment (of male?) about 1.5 times as long as second segment according to original description; occipital carina wide lamelliform medio-dorsally (Fig. 209); ocelli comparatively small, OOL 1.4 times as long as diameter of posterior ocellus; face moderately narrow (Fig. 206, 214); malar space partly absent (Fig. 209).

*Mesosoma*. Length of mesosoma 2.1 times its height; pronotal side comparatively elongate and mainly coriaceous antero-ventrally; mesoscutum not protruding anteriorly; propleuron 1.1 times as long as mesoscutum in front of tegulae (Fig. 210); antesternal carina narrow and hardly lamelliform; mesoscutum coriaceous and with satin sheen, with minute separate punctures and medio-posteriorly mainly coriaceous with some large oval punctures (Fig. 211); scutellum coriaceous.

*Wings*. First discal cell parallel-sided and indistinct posterior corner (Fig. 213; cf. ♀ Fig. 208); marginal cell of fore wing slender.

*Legs*. Hind coxa largely coriaceous, with satin sheen, but partly finely transversely striate dorsally; length of hind femur, tibia and basitarsus 4.2, 4.8 and 6.2 times their width, respectively (Fig. 212).

*Metasoma*. Missing.

*Colour*. Black or dark brown; mandible, tegulae, fore and middle legs (except ivory bases of tibiae), hind leg (but tibia with rather small ivory patch) more or less dark brown; hind tibial spurs brown; pterostigma dark brown; wing membrane moderately infuscate.

*Female*. Lectotype. Body length 15.4 mm. Head truncate medio-posteriorly in dorsal view, gradually narrowed behind eyes and weakly curved laterally (Fig. 207); temple 0.6 times as long as eye in dorsal view; vertex with a very shallow medio-posterior depression in front of occipital carina; occipital carina medium-sized (Fig. 200); antenna missing; eye glabrous; malar space very short, nearly absent; clypeus without depression, but slightly emarginate ventrally; length of mesosoma twice as long as its height; propleuron moderately slender (Fig. 201), nearly as long as mesoscutum in front of tegulae; side of pronotum mainly coriaceous, but ventrally with some rugulae and with an indistinct antero-lateral tooth; mesoscutum mainly very finely coriaceous and matt, sparsely weakly punctate; medio-posteriorly with some large round or oval punctures (Fig. 202); scutellum very finely coriaceous; mesopleuron reticulate but partly coriaceous; propodeum reticulate, medio-longitudinal carina absent; metapleuron reticulate dorsally and ventrally rugulose; ovipositor sheath as long as body, 1.5 times as long as metasoma and 4.6 times as long as hind tibia; its apical ivory part 2.1 times as long as hind basitarsus and 0.15 times as long as ovipositor sheath; incision of hypopygium deep and slit-like, 0.4 times length of hypopygium; legs dark brown, subbasal patch of middle and hind tibia ivory (Fig. 203); second-third metasomal tergites slightly red-brown apically; hind basitarsus dark brown; according to original description apical third of antenna brown-yellow.

*Variation*. Body length 10.0–16.5 mm; of fore wing 5.8–8.5 mm. Female: ovipositor sheath 0.8–1.1 times as long as body, 1.3–1.6 times as long as metasoma and 4.6–6.0 times as long as hind tibia, its apical ivory part 1.0–3.0 times as long as hind basitarsus; temple 0.5–0.7 times as long as eye in dorsal view; third antennal segment 1.3–1.6 times as long as second segment, fourth antennal segment 1.3–1.5 times as long as third segment, fifth antennal segment 1.1–1.4 times as long as third segment (Fig. 205); length of mesosoma 2.1–2.5 times as long as its height; propleuron 1.0–1.2 times as long as mesoscutum in front of tegulae; legs black or dark brown, basitarsus mainly ivory, ivory patch of hind basitarsus indistinctly. Male: vertex more distinctly depressed medio-posteriorly than female, sometimes as three depressions in front of occipital carina, medio-dorsal one medium-sized and both of lateral ones comparatively shallow temple 0.6–0.7 times as long as eye in dorsal view; third antennal segment 1.0–1.3 times as long as second segment, fourth antennal segment 1.8–2.2 times as long as third segment, fifth antennal segment 1.8–2.3 times as long as third segment; length of mesosoma 2.1–2.2 times as long as its height; propleuron 1.0–1.1 times as long as mesoscutum in front of tegulae.

#### Distribution.

China (Ningxia, Jiangsu, Shanghai, Zhejiang, Fujian, Taiwan, Hubei, Hunan, Guangxi, Hainan).

#### Biology.

Unknown. Collected in March–October.

### Gasteruption sinarum

Kieffer, 1911

http://species-id.net/wiki/Gasteruption_sinarum

Figs 217 227

Gasteruption sinarum Kieffer, 1911: 205–206, 1912: 229, 264; Hedicke 1939: 21.
Gasteruption sinense Kieffer, 1924: 77–78; Hedicke 1939: 21. syn. n.

#### Type material.

Holotype of *Gasteruption sinarum*, ♀ (BMNH), “Type”, B.M. Type 3.a.181”, “*Gasteruption sinarum* Kieff.”, “China, Walker Coll., 92–196”, “10–116”, “Determined by Dr. Kieffer”. One syntype of *Gasteruption sinense* here designated lectotype, ♀ (TARI), “Tchenkiang [= Zhenjiang, East of Nanjing; Jiangsu], 4.VII.[19]18”, “Chine, Prov. Kiangsu [= Jiangsu], Chinkiang [= Zhenjiang], Musée Heude”, “O. Piel”. Paralectotypes of *Gasteruption sinense*: 1 ♀ (TARI), same labels as lectotype, but 6.VII.1918. No other syntypes from Zhenjiang and Zi-ka-wei [= Xujiahui, Shanghai] found in MNHN, Entomological Museum Shanghai or Chinese Academy of Sciences Beijing.

#### Additional material. 

1 ♀ (ZJUH),“[China:] Liaoning, Shenyang, Botanical Garden, 3.VII.1993”; 1 ♀ (SCAU), “[China:] Inner Mongolia, Bayanchuoer, VIII.2007, Bo Qiu”; 1 ♀ (TARI), “[Inner Mongolia] Chahar, Yangklaping”, “12.VII.[19]37, O. Piel”; 1 ♀ (CAU), “[China:] Beijing, Mt. Xiang, 20.VII.1961, Ji-kun Yang”; 3 ♀ (CAU), “[China:] Beijing, China Agricultural University, VIII.1957, Fa-sheng Li & 8.VI.1957, Ji-kun Yang & 20.VIII.2002, Min Zheng”; 1 ♀ (CAU), “[China:] Beijing, Mt. Miaofeng, 7.IX.1953”; 1 ♂ (CAU), “[China:] Beijing, Gongzhufen, 22.VI.1952, Ji-kun Yang”; 1 ♀ (TARI), “[China:] Taiwan, Peiping”; 1 ♀ (ZJUH), “[China:] Beijing, Haidian, 1.VIII.1979, Tian-zu Jing”; 1 ♂ (CSCS), “[China:] Tianjin, Balitai, 5.VI.1952, Pei-yu Wang”; 1 ♀ (CSCS), “[China:] Tianjin, Nankai University Farm, 29.VI., Zong-chen Song”; 9 ♀ + 3 ♂ (TARI), “[China: Shandong,] Tsinanfou [= Jinan], Long-tong, 500–700 m, Musée Heude”; 1 ♀ (TARI), “Chine, Prov. Kiangsu [= Jiangsu], Shanghai, Musée Heude”, “Zi-ka-wei [= Xujiahui], 21.V.[19]25”, “O. Piel”; 5 ♀ (TARI), id., but 23.V.1918, 5.VII.1918, 21.VI.1920, 17.VI.1925 and 18.IX.1940; 7 ♀ (TARI) as lectotype, but 24.VII.1908, 8.X.1917, 29.V.1918, 6.VI.1918, 5.VII.1918, 11.VII.1918 and 13.V.1919; 2 ♀ (TARI), “[China:] Kiangsu [= Jiangsu], Chemo, Musée Heude”, “8 & 20.V.[19]35, O. Piel”; 1 ♀ (TARI), “[China: Jiangsu,] Nanking [= Nanjing], 15.VII.[19]18”, “Chine, Prov. Giansu [= Jiangsu], Nanking [= Nanjing], Musée Heude”, “O. Piel”; 3 ♀ (ZJUH), “[China:] Jiangsu, Yangzhou, 16.VII.1980, Lian-min Yang”; 1 ♀ (ZJUH), “[China:] Zhejiang, Changxing, 20.V.1981, Wen-liu Lv”; 1 ♂ (ZJUH), “[China:] Zhejiang, Hangzhou, 24.VI.1989, Jun-hua He”; 3 ♀ (SCAU), “[China:] Ningxia, Mt. Liupan, 3–14.VII.2009, Hua-yan Chen”; 1 ♀ (ZJUH), “[China:] Henan, Xinxiang, 19.VII.1981, Cheng-zhong Xu”; 2 ♀ (TARI), “Chine, Prov. Anhwei [=Anhui], Ou-yuen, IX.1934, O. Piel, Musée Heude”; 1 ♀ (RMNH): “[China:] Hubei, Huazhong Agricultural University, 1983, Mao-ling Sheng”; id., Jingsan, 14.VIII.1986; 1 ♂, id., but Tongcheng, 8.VI.1979; 1 ♂ (SCAU), “[China:] Hubei, Mt. Dahu, 20.VII.2009, Chun-hong Zheng”; 1 ♀ (SCAU), “[China:] Hubei, Huangmei, 25.VIII.2009, Qi Yang”; 2 ♀ (SCAU), “[China:] Hubei, Mt. Jing, 15.VII.2009, Yuan Ye”; 3 ♀ + 1 ♂ (ZJUH), “[China:] Hubei, Huazhong Agricultural University, 1983; id., Jingsan, 14.VIII.1986; id., Tongcheng, 8.VI.1979”; 1 ♀ (ZJUH), “[China:] Hunan, Changsha, 14.IX.1980, Xin-wang Tong”; 1 ♀ (SCAU), “[China:] Hunan, Mt. Huping, Shinianzigou, 9.VII.2009, Ya-li Tang”; 1 ♀ (ZJUH), “[China:] Hunan, Liuyang, 2.VII.1983, Xin-wang Tong”; 2 ♀ (ZJUH), “[China:] Hunan, Shashi, 27.VI.1983”; 2 ♀ (ZJUH), “[China:] Hunan, Liuyang, 28.V.1984 Xin-wang Tong”; 1 ♀ (ZJUH), “[China:] Hunan, 28.IX.1985, Xin-wang Tong”; 1 ♀ (ZJUH), “[China:] Hunan, Liuyang, 27.IX.1985, Xin-wang Tong”; 1 ♀ (ZJUH), “[China:] Guizhou, Huishui, VII.1987, Ji-ming Chu, No. 874969”; 1 ♀ (ZJUH), “[China:] Guizhou, Meitan, 24.V.1943, Ru-zuo Zhu”; 1 ♂ (ZJUH), “[China:] Guizhou, Meitan, 1.V.1943”; 1 ♀ (SCAU), “[China:] Guizhou, Mayang River, 2.X.2007, Cui-hong Xie”; 1 ♂ (ZJUH), “[China:] Guangxi, Guilin, 22.IX.1987, Jun-hua He”; 1 ♀ (ZJUH), “[China:] Guangxi, Mt. Yi, 3.XI.1943, Ru-zuo Zhu”; 3 ♀ (SCAU), “[China:] Guangdong, Meizhou, Meixian, 14.VII.2007, Cui-hong Xie”; 3 ♀ (SCAU), “[China:] Guangdong, Mt. Guanyin, 15.IX.2007, Zai-fu Xu”; 8 ♀ + 1 ♂ (SCAU), “[China:] Guangdong, Nanling Nature Reserve, 16.X.2007, Zai-fu Xu”; 2 ♀ + 1 ♂ (SCAU), “[China:] Guangdong, Qingyuan, Huangchuansanxia, 18.IX.2004, Zai-fu Xu”; 4 ♀ + 2 ♂ (SCAU), “[China:] Guangdong, Qingyuan, 11.IX.2004, Zai-fu Xu”.

#### Diagnosis.

**Figures 217–224.**
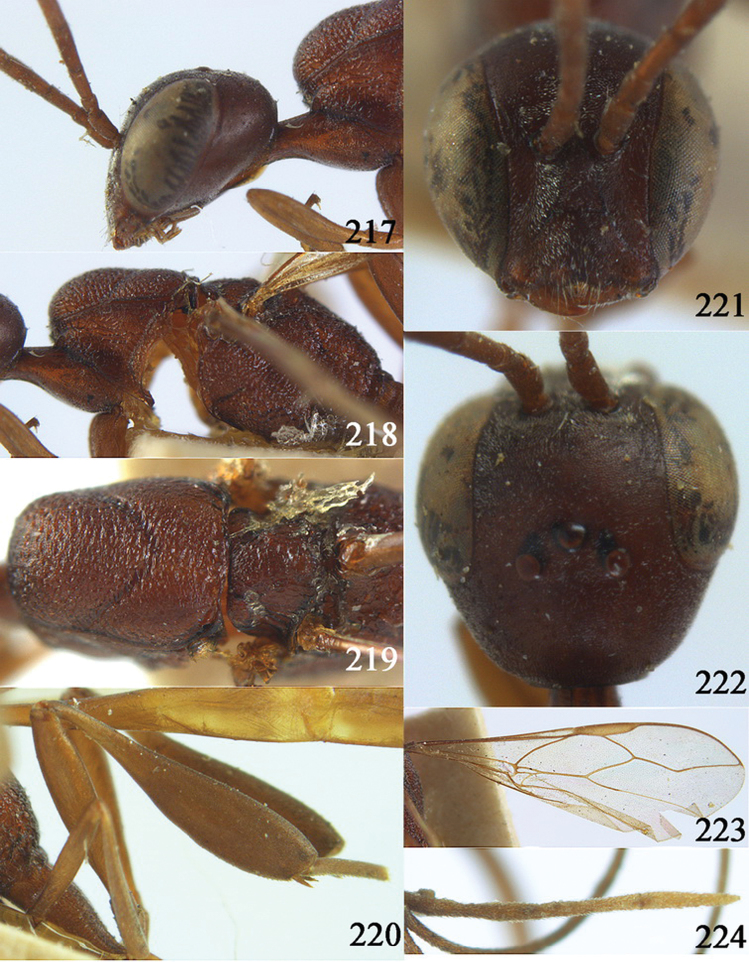
*Gasteruption sinarum* Kieffer, 1911, holotype, female. **217** head lateral **218** mesosoma lateral **219** mesoscutum dorsal **220** hind legs **221** head anterior **222** head dorsal **223** fore wing **224** apex of ovipositor sheath.

**Figures 225–227.**
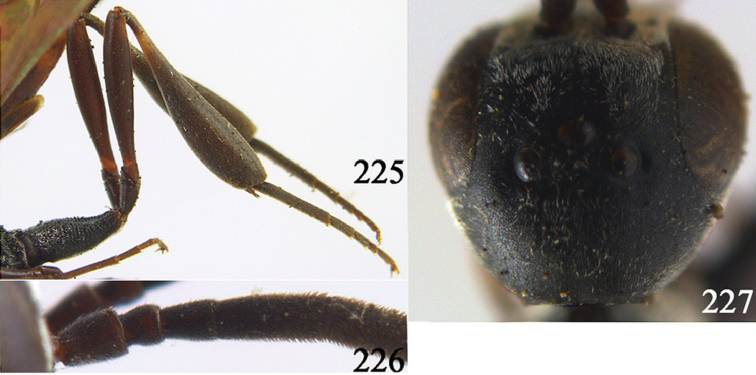
*Gasteruption sinarum* Kieffer, 1911, male, Shandong. **225** hind legs **226** the first to fourth antennal segments **227** head dorsal.

Apex of ovipositor sheath narrowly ivory (Fig. 224); ovipositor sheath 1.1–1.2 times as long as body; occipital carina narrow, non-lamelliform medio-dorsally (Fig. 217) and protruding laterally (Fig. 217); propleuron robust, 0.8–1.0 times as long as mesoscutum in front of tegulae (Fig. 218); antesternal carina narrow; head, laterally mesosoma and scape brown; head in anterior view not protruding below lower level of eyes and mandibular condylus near lower level of eyes (Fig. 221); in lateral viewcondylarincision of malar space close to eye (Fig. 217); clypeal ventral depression obsolescent and lateral corners rather protruding forwards; eyes glabrous; fourth and fifth antennal segment 1.2–1.5 and 1.0–1.5 (♀) (of ♂ 1.4–2.0 and 1.4–1.8) times as long as third segment, respectively (♂ Fig. 226); apical antennal segment of ♀ 1.1 [absent in HT] times as long as third antennal segment and brown, as colour of medial segments; antenna of female brown; mesoscutum and head with satin sheen, head dorsally very finely punctulate and mesoscutum coriaceous-punctulate between many large punctures, punctate-rugose medio-posteriorly (Figs 219, 222; cf. ♂ Fig. 227); hind coxa transversely rugose dorsally, interspaces smooth or rugulose; hind tibia rather slender, brown subbasally, without an ivory ring, with a distinctly convex ventral border (Fig. 220); hind basitarsus comparatively long and entirely brown (Fig. 225); hind tibial spurs dark brown; apical half of hypopygium incised.

#### Description.

Holotype of *Gasteruption sinarum*, female, body length 11 mm.

*Head*. Vertex and frons with satin sheen and densely and very finely punctulate, moderately convex medio-posteriorly; head gradually narrowed behind eyes; temple 0.7 times as long as eye in dorsal view (Fig. 222); fourth antennal segment 1.5 times as long as third segment and 0.9 times as long as second and third segments combined, fifth antennal segment 1.5 times as long as third segment, third antennal segment 1.7 times as long as second segment; occipital carina narrow, non-lamelliform medio-dorsally (Fig. 217); OOL 1.5 times as long as diameter of posterior ocellus; face rather wide (Fig. 221); minimum width of malar space 0.2 times as long as second antennal segment; clypeus without distinct depression, its lateral corners protruding forwards and medio-ventrally emarginate; eye glabrous.

*Mesosoma*. Length of mesosoma twice its height; pronotal side moderately high and largely moderately reticulate-rugose, posteriorly with coriaceous patches, with minute acute antero-lateral tooth; mesoscutum not protruding anteriorly; propleuron robust, 0.9 times as long as mesoscutum in front of tegulae (Fig. 218); antesternal carina narrow and hardly lamelliform; mesoscutum anteriorly coarsely punctate and with coriaceous-punctulate interspaces, remainder largely punctate-rugose and with satin sheen, rather matt (Fig. 219); scutellum coriaceous and with some weak transverse rugulae.

*Wings*. Fore wing: first discal cell parallel-sided and with outer posterior corner rounded (Fig. 223), glabrous; vein SR1 distinctly bent.

*Legs*. Hind coxa with satin sheen, slender, coriaceous and dorsally transversely rugose; length of hind femur, tibia and basitarsus 4.9, 4.8 and 6.9 times their width, respectively (Fig. 220); middle tarsus incomplete in HT, 1.2 times as long as middle tibia in China specimen; middle femur subparallel-sided and slenderer than fore femur.

*Metasoma*. Ovipositor sheath 4.9 times as long as hind tibia, 1.5 times metasoma and as long as body; ivory part of sheath 0.7 times as long as hind basitarsus; apical half of hypopygium slit-shaped incised.

*Colour*. Dark brown; antenna, fore and middle legs, hind trochanter, trochantellus and femur and first-sixth metasomal segments yellow-brown; legs without ivory patches; pterostigma pale brown.

*Male* (described a male from Shandong). Similar to female, but mesoscutum with some rather coarse punctures. Third antennal segment as long as second segment (Fig. 226); fourth segment 3.5 times as long as third segment (as fifth segment), and 1.8 times as long as second and third segments combined; propleuron rather robust and 0.9 times as long as mesoscutum in front of tegulae; hind leg coloured as in female with hind tibia slightly less swollen (Fig. 225); apical tergite not impressed apically; paramere brown apically; body length 13.2 mm.

*Variation*. Female: body length 10.5–16.0 mm, of ovipositor sheath 1.1–1.2 times as long as body, 4.8–6.0 times as long as hind tibia, 3.0–3.9 times as long as hind tibia and tarsus combined and 1.5–1.9 times as long as metasoma; of its apical ivory part 0.3–1.0 times as long as hind basitarsus; temple about 0.6 times as long as eye in dorsal view; third antennal segment 1.7–2.0 times as long as second segment, fourth antennal segment 1.2–1.5 times as long as third segment, fifth antennal segment 1.0–1.3 times as long as third segment; eye setose or nearly so, but sometimes glabrous; minimum width of malar space 0.2–0.3 times as long as second antennal segment; length of mesosoma 1.9–2.1 times as long as its height; propleuron about 0.8–1.0 times as long as mesoscutum in front of tegulae; length of hind femur, tibia and basitarsus 4.8–5.1, 4.5–4.7 and 5.5–5.7 times their width, respectively; middle tarsus 1.2–1.3 times as long as middle tibia; hind tibia sometimes with indistinct ventral subbasal patch ivory to brown; the specimen from Henan subapical of hind basitarsus ivory. Male: body length 14.5–15.5 mm; temple 0.6–0.7 time as long as eye in dorsal view; third antennal segment 1.2–1.4 times as long as second segment, fourth antennal segment 1.9–2.0 times as long as third segment, fifth antennal segment 1.8 times as long as third segment; length of mesosoma 1.9–2.0 times as long as its height; length of hind femur, tibia and basitarsus 4.5–5.0, 4.3–4.8 and 5.5–6.1 times their width, respectively.

#### Distribution.

China (Liaoning, Inner Mongolia, Beijing, Tianjin, Ningxia, Henan, Jiangsu, Shanghai, Shandong, Zhejiang, Anhui, Hubei, Hunan, Guangdong, Guangxi, Guizhou).

#### Biology.

Unknown. Collected in May–November.

### Gasteruption sinepunctatum sp. n.

urn:lsid:zoobank.org:act:5264515C-180B-4E9E-A5E9-1A59951AA61F

http://species-id.net/wiki/Gasteruption_sinepunctatum

Figs 228 240

#### Type material.

Holotype, ♀, (ZJUH), “[China:] Zhejiang, Mt. Tianmu, 2.IX.1987, Jin-jiang Fan”. Paratypes: “[China:] Jilin, Daxinggou, 7.VIII.2005”; 1 ♀ (TARI), “[China:] Taiwan, Nantou, Lienhuachi, 650 m, IV.1984, K.S. Lin & K.C. Chou, Malaise trap”; 1 ♀ (TARI), “[China:] Taiwan, 2.IV.1921, Tokuyo coll.”; 1 ♂ (TARI), “[China:] Formosa [= Taiwan], Taito, 25.II–27.III.1919, S. Inamura, J. Sonan, M. Yoshino”; 1 ♂ (TARI), “[China:] Taiwan, Takeyama, 16.V.1926, J. Sonan”; 1 ♀ (TARI), “[China:] Taiwan, Chipon, 9.X.1938, J. Sonan”; 1 ♀ (CSCS), “[China:] Tibet, Cibagou, Chayu, 22.VI.2009, 28°53.816'N, 97°27.771'E, alt. 2697 m, Mei-cai Wei”.

#### Diagnosis.

**Figures 228–236.**
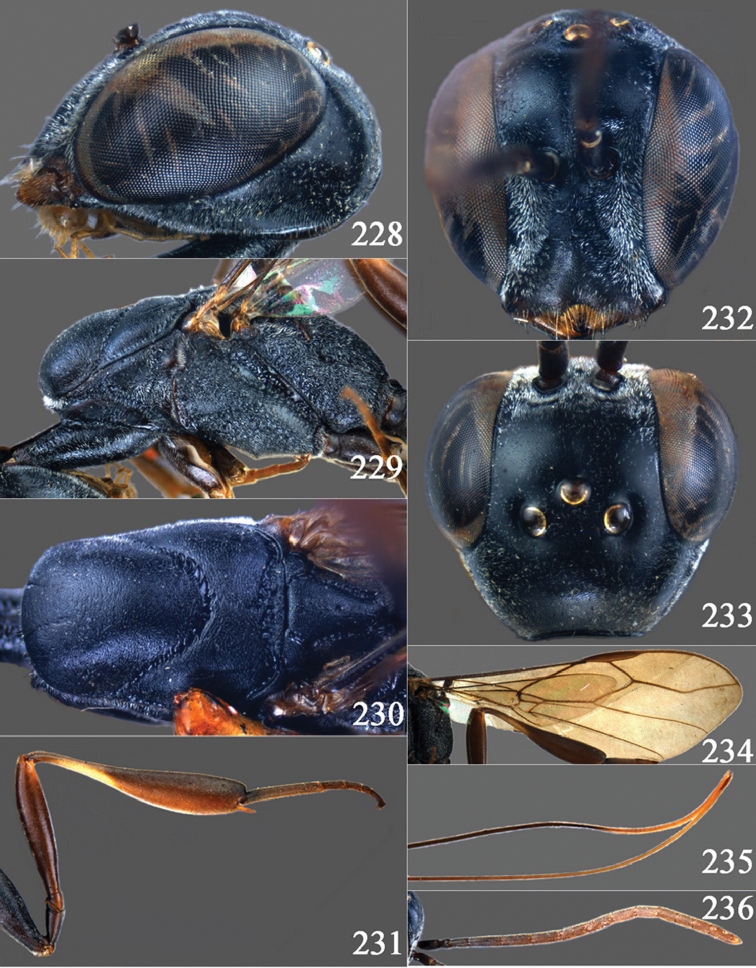
*Gasteruption sinepunctatum* sp. n. holotype, female. **228** head lateral **229** mesosoma lateral **230** mesoscutum dorsal **231** hind leg **232** head anterior **233** head dorsal **234** fore wing **235** apex of ovipositor and its sheath **236** antenna.

Anterior half of mesoscutum very finely coriaceous, rarely very finely transversely rugulose, and with at most some shallow punctures; fourth antennal segment of ♀ 1.7–1.9 times as long as third antennal segment (Figs 236, 238); head more narrowed in dorsal view, resulting in a more transverse head (Figs 233, 239); head dorsally more or less shiny and smooth; vertex slightly convex medio-posteriorly in lateral view or occipital carina narrow lamelliform medio-dorsally (Fig. 228); frons smooth or micro-sculptured; pale apical part of ovipositor sheath 1.3–2.8 times as long as hind basitarsus; subbasal pale patch of hind tibia distinctly differentiated; ovipositor sheath 0.8–1.3 times as long as body and 1.2–1.7 times as long as metasoma; ovipositor slender and nearly straight or gradually upcurved apically; apical 0.3 of hypopygium incised.

#### Description.

Holotype, female, body length 15 mm, of fore wing 8 mm.

**Figures 237–240.**
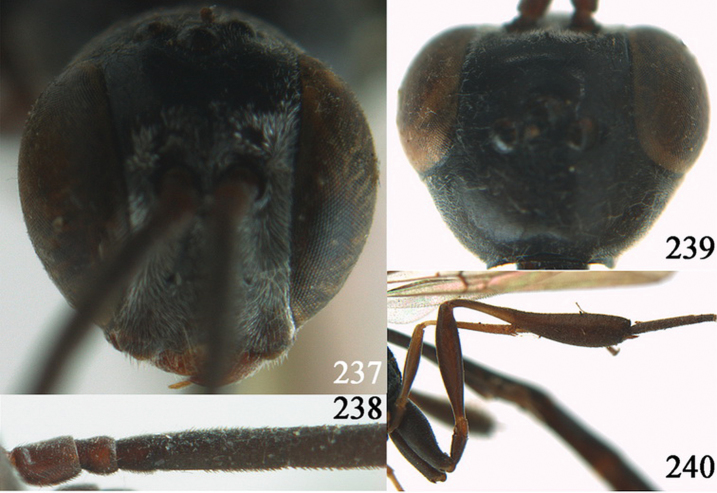
*Gasteruption sinepunctatum* sp. n. paratype, male. **237** head anterior **238** the first to fourth antennal segments **239** head dorsal **240** hind leg.

*Head*. Head more or less truncate, directly narrowed behind eyes and almost straight laterally (Fig. 233); temple at most 0.5 times as long as eye in dorsal view; vertex and frons with satin sheen and rather sparsely very finely punctulate; vertex moderately convex posteriorly and without any depression medio-posteriorly; occipital carina narrow and non-lamelliform medio-dorsally (Fig. 228); third antennal segment 1.3 times as long as second segment, fourth antennal segment 1.8 times as long as third segment, fifth antennal segment 1.6 times as long as third segment (Fig. 236); eye glabrous; OOL 1.1 times as long as diameter of posterior ocellus; minimum width of malar space 0.2 times as long as second antennal segment; clypeus without depression (Figs 232, 237).

*Mesosoma*. Length of mesosoma 2.1 times as long as its height; propleuron 0.9 times as long as mesoscutum in front of tegulae (Fig. 229); side of pronotum mainly coriaceous, but medio-ventrally with rugose; mesoscutum coriaceous and matt, laterally slightly rugulose (Fig. 230); medio-posteriorly regularly rugulose; scutellum coriaceous and matt; propodeum reticulate, medio-longitudinal carina absent.

*Wings*. Fore wing: first discal cell parallel-sided and with outer posterior corner rounded (Fig. 234).

*Legs*. Hind coxa matt, moderately slender and dorsally transversely rugulose; length of hind femur, tibia and basitarsus 4.6, 5.5 and 6.8 times their width, respectively (Fig. 231); middle tarsus 1.3 times as long as middle tibia.

*Metasoma*. Ovipositor sheath 0.9 times as long as body, 1.4 times as long as metasoma, 4.2 times as long as hind tibia and 2.5 times combined hind tarsus and tibia; its apical ivory part 2.7 times as long as hind basitarsus and 0.2 times as long as ovipositor sheath; hypopygium rather slit-shaped incised apically.

*Colour*. Black; antenna dark brown to brown; legs brown, fore tarsus yellow, hind coxa dark brown, large ventral subbasal patch of hind tibia ivory or yellow; second-fifth metasomal tergites brown ventrally; ovipositor sheath black-brown, but largely apex ivory or yellow (Fig. 235); pterostigma dark brown.

*Male* (described after a male from Taiwan). Similar to female, but mesoscutum with some rather coarse punctures. Third antennal segment as long as second segment (Fig. 238); fourth segment 3.5 times as long as third segment (as fifth segment), and 1.8 times as long as second and third segments combined; propleuron rather robust and 0.9 times as long as mesoscutum in front of tegulae; hind leg coloured as in female with hind tibia slightly less swollen (Fig. 240); apical tergite not impressed apically; paramere brown apically; body length 13.2 mm.

#### Distribution. 

China (Jilin, Zhejiang, Taiwan, Tibet).

#### Etymology. 

Named after the very finely sculptured mesoscutum lacking any punctures (Latin: “sine” is “without” and “ punctatum “ is having punctures, dots or spots).

#### Biology.

Unknown. Collected in February–June, August–October.

### Gasteruption sinicola

(Kieffer, 1924), comb. n.

http://species-id.net/wiki/Gasteruption_sinicola

Figs 241 251

Trichofoenus sinicola Kieffer, 1924: 79; Hedicke 1939: 45.

#### Type material.

Holotype ♀ from China (“Tchenkiang” [= Chenkiang, now Zhenjiang, (East of Nanjing, Jiangsu) 29.IX., O. Piel] not found in O. Piel collection (TARI), MNHN, Entomological Museum at Shanghai or Chinese Academy of Sciences at Beijing and is probably lost.

#### Additional material.

China: topotypical series (TARI, RMNH) from Jiangsu (“Chusan, Chenkiang [= Zhenjiang]” collected by O. Piel in 1930–31 and “Tchenkiang [= Zhenjiang], 18.VI.[19]18 & 12.X.[19]17”; 3 ♀ +1 ♂ (ZJUH), “[China:] Fujian, Fuzhou, 17.VIII.1990, Chang-ming Liu”; 2 ♀ (ZJUH), “[China:] Fujian, Fuzhou, 3.X.1990, Nai-quan Lin”; 2 ♀ + 2 ♂ (ZJUH), “[China:] Fujian, Fuzhou, 25.IV.1991, Chang-ming Liu”; 1 ♀ (ZJUH), “[China:] Fujian, Fuzhou, 20.IV.1993, Chang-ming Liu”; 1 ♀ (ZJUH), “[China:] Fujian, Fuzhou, 10.VI.1988, Sa-ping Yi”; 3 ♂ (ZJUH), “[China:] Fujian, Fuzhou, 20.VI.1988 &15.VI.1988 & 15.VII.1993, Nai-quan Lin”; 1 ♂ (ZJUH), “[China:] Fujian, Fuzhou, Jinshan, 6.VI.1989, Xiu-fu Zhao”; 2 ♂ (ZJUH), “[China:] Fujian, Fuzhou, Jinshan, 12.VII.2004, Chang-ming Liu”; 1 ♀ (ZJUH), “[China:] Fujian, Jiangle, Mt. Longqi, 1.VII.1991, Chang-ming Liu”; 1 ♂ (SCAU), “[China:] Guangdong, Nanling Nature Reserve, V.2011, Zai-fu Xu”; 1 ♀ (SCAU), “[China:] Guangdong, Wuhua, Mt. Qimufeng, 31.VII.2003, Yan-xia Song”; 1 ♀ (SCAU), “[China:] Hainan, Mt. Diaoluo, 29.V.2007, Bin Xiao”; 2 ♂ (SCAU), “[China:] Ningxia, Mt. Liupan, 3–14.VII.2009, Hua-yan Chen”; 1 ♀ (ZJUH), “[China:] Hunan, Changsha,V.1974, Xin-wang Tong”; 1 ♀ (RMNH), “[China:] Hunan, Linyang, near Changsha, 30. VI.1985, RMNH’11”.

#### Diagnosis.

**Figures 241–248.**
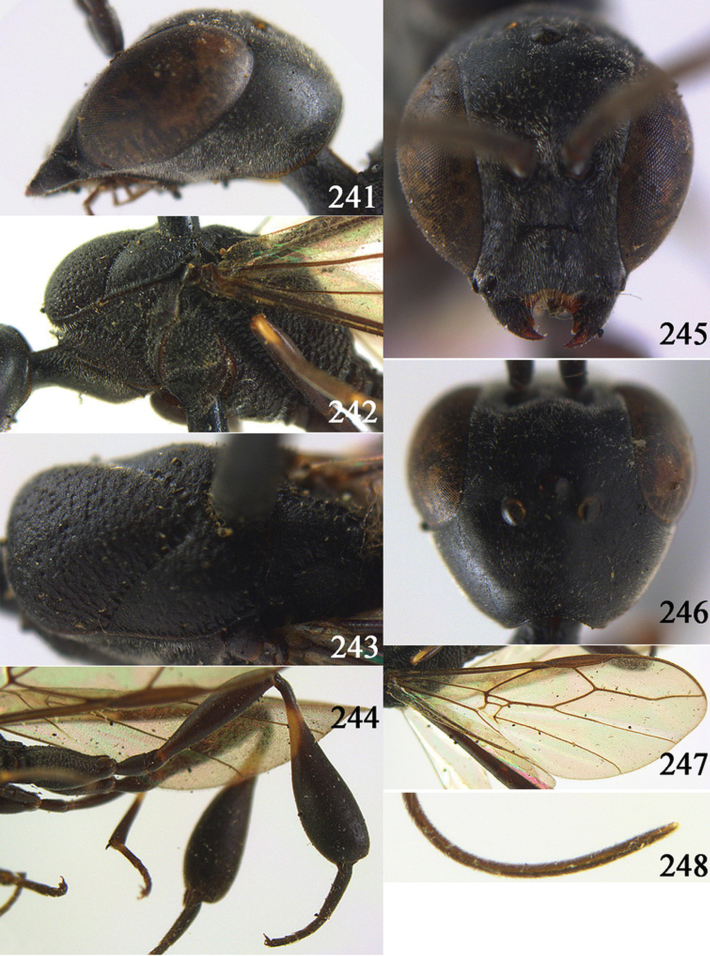
*Gasteruption sinicola* (Kieffer, 1924), female, Jiangsu. **241** head lateral **242** mesosoma lateral **243** mesoscutum dorsal **244** hind legs **245** head anterior **246** head dorsal **247** fore wing **248** apex of ovipositor sheath.

Apex of ovipositor sheath black or dark brown (Fig. 248); ovipositor sheath about 0.8 times as long as hind tibia and tarsus combined, about 0.4–0.5 times as long as metasoma and 1.3–1.5 times as long as hind tibia; eyes shortly setose; head in dorsal and lateral view rather conical elongate (Figs 241, 246, 251); malar space 0.2–0.3 times as long as second antennal segment (= pedicellus); clypeus without depression (Fig. 245); vertex and frons shiny and largely smooth (Figs 246, 251), at most rather remotely finely punctulate; occipital carina indistinct dorsally (Figs 246, 251); propleuron 0.8–1.0 times as long as mesoscutum in front of tegulae moderately slender (Fig. 242); pronotum with small antero-lateral tooth; mesoscutum very finely and densely coriaceous and with isolated punctures (typical) to “crater-like” punctate (Fig. 243); notauli very shallow and narrow in ♀ but wider and more or less crenulate in ♂, mesoscutum finely; antesternal carina narrow; median carina of propodeum absent (but sometimes a slightly elevated smooth median line), if present then surrounding reticulate-rugose and carina similarly developed; hind tibia robust; hind basitarsus black; hind tibia with ivory subbasal patch (♂ ♀); first discal cell of fore wing more or less narrowed apically (Fig. 247); only apical 0.1 of hypopygium widely emarginate.

#### Description.

Described after a female from Jiangsu, Zhenjiang (ex coll. O. Piel), body length 10.0 mm.

*Head*. Vertex and frons shiny and largely smooth (Fig. 246), at most rather remotely finely punctulate and without a depression medio-posteriorly; head directly narrowed behind eyes (Fig. 246); temple 0.7 times as long as eye in dorsal view; fourth antennal segment 1.1 times as long as third segment and 0.7 times as long as second and third segments combined, fifth antennal segment 0.9 times as long as third segment, third antennal segment twice as long as second segment; occipital carina indistinct dorsally (Fig. 241); OOL 1.3 times as long as diameter of posterior ocellus; face moderately wide (Fig. 245); minimum width of malar space 0.2 times as long as second antennal segment (Fig. 241); clypeus with small triangular depression and slightly emarginate; eye with short setose.

*Mesosoma*. Length of mesosoma 1.6 times its height; pronotal side moderately high and ventrally rugulose-coriaceous, with small antero-lateral tooth; mesoscutum not protruding anteriorly; propleuron robust, 0.8 times as long as mesoscutum in front of tegulae (Fig. 242); antesternal carina narrow and hardly lamelliform; mesopleuron rugulose dorsally; mesoscutum densely coriaceous, with more or less crater-like punctures and matt, lateral lobes with fewer and finer punctures than middle lobe (Fig. 243); mesoscutum posteriorly coarsely punctate; scutellum coriaceous; propodeum with distinct median carina.

*Wings*. First discal cell parallel-sided and with posterior corners angulate (Fig. 247).

*Legs*. Hind coxa rather matt, moderately robust, coriaceous-rugulose; hind tibia strongly widened (Fig. 244); length of hind femur, tibia and basitarsus 4.1, 3.4 and 4.8 times their width, respectively; middle tarsus normal (Fig. 244), slightly longer than middle tibia; middle femur subparallel-sided and slightly slenderer than fore femur.

*Metasoma*. Ovipositor sheath 0.3 times as long as body, 0.4 times as long as metasoma, 0.5 times as long as fore wing and 1.3 times as long as hind tibia; sheath dark brown apically; hypopygium shallow v-shaped incised apically.

*Colour*. Black or black-brown; tegulae and legs largely dark brown, but hind tibial spurs brown and hind tibia with large ivory subbasal patch (but narrow dorsally); pterostigma dark brown.

**Figures 249–251.**
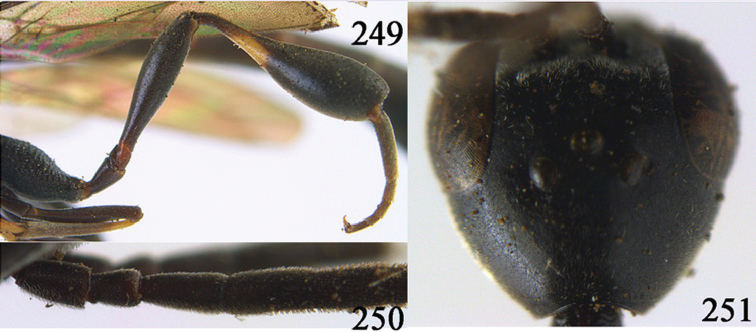
*Gasteruption sinicola* (Kieffer, 1924), male, Jiangsu. **249** hind leg **250** the first to fifth antennal segments **251** head dorsal.

*Male* (described after a male from Jiangsu). Very similar to female, but slightly more coarsely sculptured. Third antennal segment about twice as long as second segment (Fig. 250); fourth segment 1.1 times as long as third segment (fifth segment as long as third), and 0.8 times as long as second and third segments combined; propleuron robust and 0.9 times as long as mesoscutum in front of tegulae; hind leg coloured as in female with hind tibia equally swollen (Fig. 249); eighth tergite widely transversely impressed apically; paramere black apically; body length 12.9 mm.

*Variation*. Female: body length 9.5–13.0 mm, of ovipositor sheath 0.2–0.3 times as long as body, 0.4–0.5 times as long as metasoma and 1.3–1.5 times as long as hind tibia. Temple 0.6–0.7 times as long as eye in dorsal view; third antennal segment 1.5–2.0 times as long as second segment, fourth antennal segment 1.1 times as long as third segment, fifth antennal segment 0.9–1.0 as long as third segment. Male: body length 11.0–13.0 mm, very similar to female.

#### Distribution.

China (Ningxia, Jiangsu, Fujian, Hunan, Guangdong, Hainan).

#### Biology.

Unknown. Collected in April–October.

### Gasteruption strigosum sp. n.

urn:lsid:zoobank.org:act:77D9D3C0-C4DF-4F74-9A63-80508E62FF30

http://species-id.net/wiki/Gasteruption_strigosum

Figs 252 264

#### Type material.

Holotype, ♀ (SCAU): “[China:] Hainan, Baisha, Mt. Jiujialing, 17.VII.2010, Hua-yan Chen”. Paratype: 1 ♀ (ZJUH), “[China:] Fujian, Mt. Xiaowuyi, 26–29. VII.1983, Yun Ma”; 1 ♂ (ZJUH), “[China:] Fujian, Mt. Wuyi, 20.VII.1985, Nai-quan Lin”; 1 ♀ (ZJUH), “[China:] Hainan, Mt. Wuzhi, Shuiman, 16–21.V.2007, Jie Zeng”; 1 ♀ (CSCS), “[China:] Yunnan, Simao, Jingdong, Wenjing, 29.IV.2005, Li Ma”; 1 ♂ (CSCS), “[China:] Yunnan, Baoshan, Mangkuan, Sandieshui, 18.VII.2006, Rui Zhang”.

#### Diagnosis.

**Figures 252–259.**
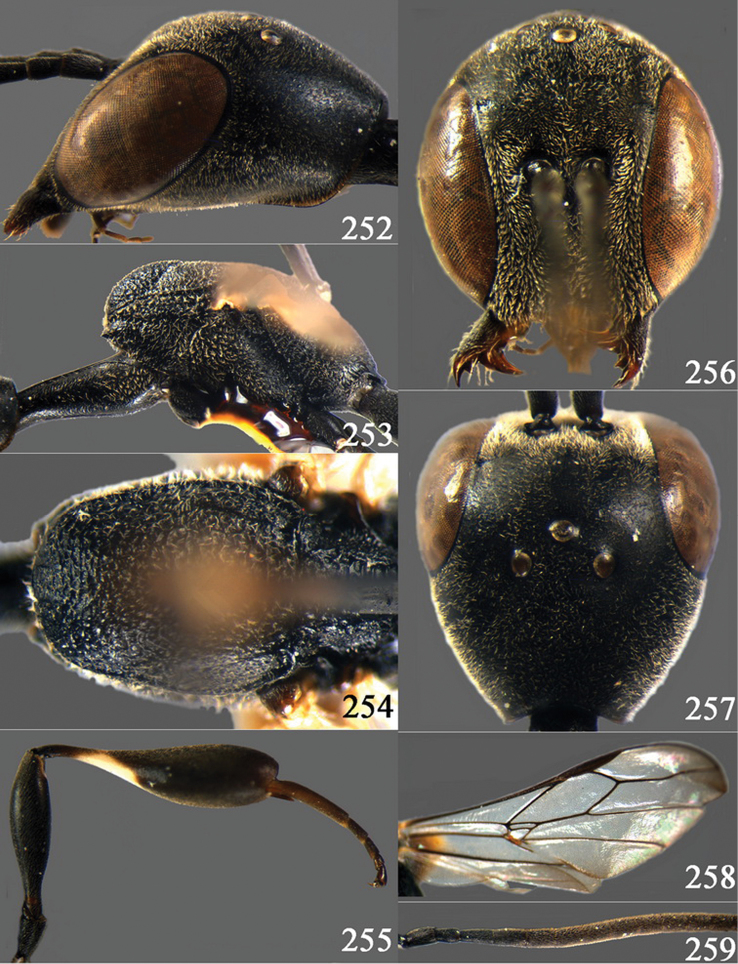
*Gasteruption strigosum* sp. n. holotype, female. **252** head lateral **253** mesosoma lateral **254** mesoscutum dorsal **255** hind leg **256** head anterior **257** head dorsal **258** fore wing **259** antenna.

Propleuron slender and 1.3–1.6 times as long as mesoscutum in front of tegulae (Fig. 253); head conically narrowed behind eyes (Fig. 257), truncate medio-posteriorly or nearly so; vertex very superficially punctulate and with satin sheen (Fig. 257); malar space 0.1–0.3 times as long as second antennal segment (= pedicellus); clypeus at most with a small depression (Figs 256, 262); apical antennal segment at most 1.2 times as long as third antennal segment and its colour similar to colour of medial segments; hind basitarsus comparatively long and narrow (Fig. 255); interspaces of mesoscutum more or less irregularly coriaceous and without punctures or densely transversely rugulose or rugose; hind tibia about 1.2 times as long as hind femur and trochanter and less swollen (Fig. 255); median carina of propodeum absent (but sometimes a slightly elevated smooth median line), if present then surrounding reticulate-rugose and carina similarly developed; ovipositor sheath 1.6–1.7 times as long as hind tibia and tarsus combined, 0.7–0.8 times as long as metasoma and 2.6–2.7 times as long as hind tibia; apical 0.1 of hypopygium incised; occipital carina obsolescent to narrowly lamelliform medio-dorsally (Fig. 252); apex of ovipositor sheath black or dark brown.

#### Description.

Holotype, female, body length 12.8 mm, of fore wing 5.5 mm.

*Head*. Head gradually narrowed behind eyes and indistinct curved laterally (Fig. 257); temple 0.9 times as long as eye in dorsal view; vertex and frons matt and coriaceous; vertex flat without any depression medio-posteriorly; occipital carina undeveloped and non-lamelliform medio-dorsally (Fig. 252); third antennal segment 1.3 times as long as second segment; fourth antennal segment as long as third segment; fifth antennal segment 0.8 times as long as third segment (Fig. 259); eye setose; OOL 2.0 times as long as diameter of posterior ocellus; minimum width of malar space 0.2 times as long as second antennal segment; clypeus without depression.

*Mesosoma*. Length of mesosoma 2.1 times as long as its height; propleuron slender (Fig. 253), 1.3 times as long as mesoscutum in front of tegulae; side of pronotum reticulate-rugose to reticulate, with a distinct antero-lateral tooth; the whole mesoscutum coarsely reticulate-rugose, interspaces coriaceous (Fig. 254); scutellum punctate-rugose; propodeum reticulate, medio-longitudinal carina distinct.

*Wings*. Fore wing: first discal cell parallel-sided and with outer posterior corner rounded (Fig. 258).

*Legs*. Hind coxa satin sheen, moderately slender and dorsally reticulate-rugulose to transversely rugose; length of hind femur, tibia and basitarsus 3.3, 3.9 and 5.5 times their width, respectively (Fig. 255); middle tarsus 1.2 times as long as middle tibia.

*Metasoma*. Ovipositor sheath 0.5 times as long as body, 0.8 times as long as metasoma, 2.7 times as long as hind tibia and 1.6 times as long as hind tibia and tarsus combined; hypopygium shallow v-shaped incised apically.

*Colour*. Black; mandible dark brown; antenna black to dark brown; wing membrane subhyaline, pterostigma and veins brown; fore and middle legs dark brown to brown, but base of tibiae and basitarsi pale; basal 0.4 of hind tibia ivory ventrally; metasoma dark brown ventrally; ovipositor sheath entire black.

**Figures 260–264.**
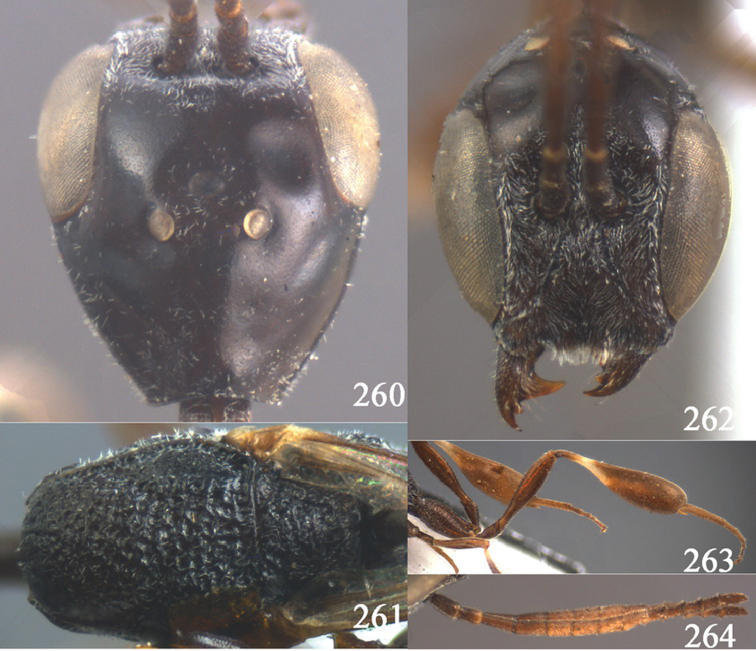
*Gasteruption strigosum* sp. n. paratype, male. **260** head dorsal **261** mesoscutum dorsal **262** head anterior **263** hind legs **264** antennae.

*Male* (described after a male from Fujian). Body length 11 mm; head gradually narrowed behind eyes (Fig. 260); temple as long as eye in dorsal view; vertex and frons with satin sheen and coriaceous (Fig. 260); vertex flat without any depression medio-posteriorly; occipital carina narrow and non-lamelliform medio-dorsally; third antennal segment 1.4 times as long as second segment; fourth antennal segment 0.9 times as long as third segment; fifth antennal segment 0.8 times as long as third segment (Fig. 264); eye setose; propleuron 1.5 times as long as mesoscutum in front of tegulae; side of pronotum mainly reticulate, with a distinct antero-lateral tooth; the whole mesoscutum densely reticulate-rugose, interspaces coriaceous; scutellum punctate-rugose (Fig. 261); hind coxa with satin sheen, moderately slender and dorsally transversely rugose; length of hind femur, tibia and basitarsus 4.1, 3.8 and 5.6 times their width (Fig. 263); the antenna brown; the colour of legs than female shallow; especially metasoma brown.

*Variation*. Female: the paratypes have no obvious differences. Body length 13–14 mm, ovipositor sheath 0.5 times as long as body, 0.7–0.8 times as long as metasoma, 2.6–2.7 times as long as hind tibia and about 1.6–1.7 times as long as hind tibia and tarsus combined.

#### Distribution.

Oriental China (Fujian, Hainan, Yunnan).

#### Biology.

Unknown. Collected in April, May and July.

#### Etymology.

Named after the elongate propleuron (Latin: “strigosus” = “lean, thin”).

### Gasteruption subhamatum

Pasteels, 1958

http://species-id.net/wiki/Gasteruption_subhamatum

Figs 265–272

Gasteruption subhamatum Pasteels, 1958: 206–207, fig. 28.

#### Type material.

Holotype, ♀ (USNM), “[E. Malaysia: Sabah], Borneo, Sandakan, Baker”, “Type”, “*Gasteruption subhamatum* n. sp., J. Pasteels det., 1955”.

#### Additional material.

1 ♀ (SCAU), “[China:] Hainan, Mt. Jianfengling, 7.VI.2007, Jing-xian Liu”; 1 ♀ (SCAU), “[China:] Hainan, Mt. Diaoluo, 12–13.VII.2010, Hua-yan Chen”.

#### Diagnosis.

**Figures 265–272.**
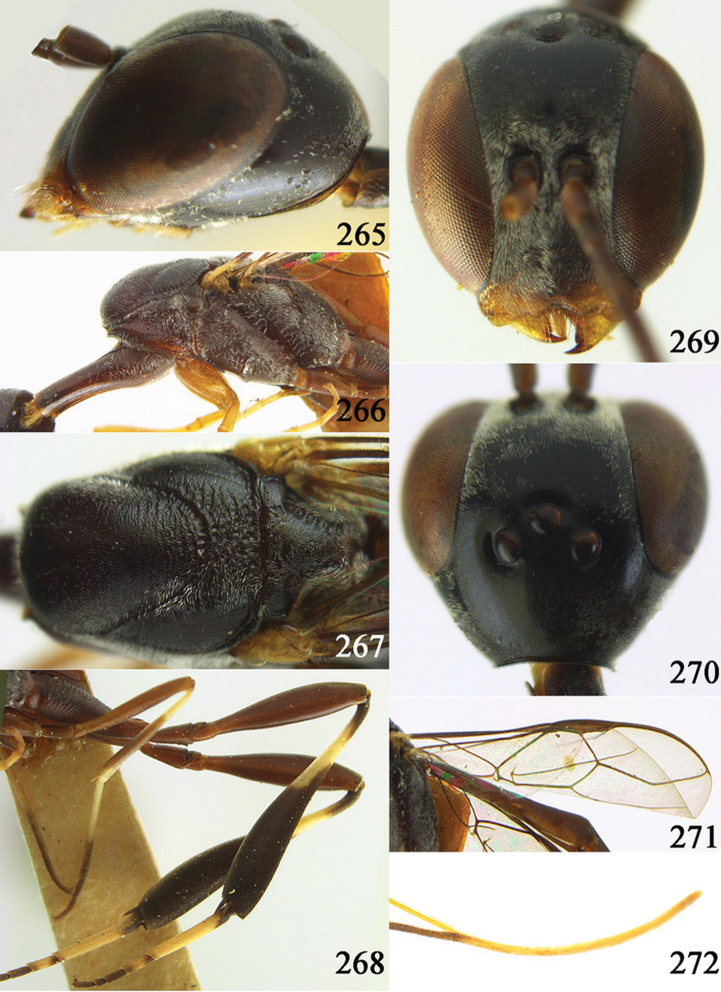
*Gasteruption subhamatum* Pasteels, 1958, holotype, female. **265** head lateral **266** mesosoma lateral **267** mesoscutum dorsal **268** hind legs **269** head anterior **270** head dorsal **271** fore wing **272** apex of ovipositor sheath.

Apical fifth of ovipositor sheath ivory (Fig. 272); ovipositor sheath 1.1–1.2 times as long as body; occipital carina narrow and not lamelliform; head rather transverse in dorsal view; vertex slightly convex in front of occipital carina (Fig. 265); without a medio-posterior depression (Fig. 270); third antennal segment of both sexes about 3 times as long as wide; fourth antennal segment slender and about 1.6 times as long as third segment and third segment 1.7 times as long as second segment; frons and vertex shiny and superficially finely punctate; propleuron 1.2–1.4 times as long as mesoscutum in front of tegulae (Fig. 266); first discal cell of fore wing absent (Fig. 271); hind tibia weakly inflated and elongate (Fig. 268); second hind tarsal segment largely ivory; hind basitarsus elongate (Fig. 268); apical 0.4 of hypopygium incised, slit-shaped.

#### Description.

Holotype, female, body length 13.9 mm, of fore wing 6.6 mm.

*Head*. Head rather transverse (Fig. 270); vertex shiny and superficially finely punctate, slightly convex and without a distinct depression medio-posteriorly; frons evenly convex, shiny and superficially finely punctate (Fig. 270); head directly narrowed behind eyes; temple 0.5 times as long as eye in dorsal view; fourth antennal segment 1.6 times as long as third segment and as long as second and third segments combined, fifth antennal segment 1.5 times as long as third segment, third antennal segment 1.7 times as long as second segment and 3.2 times as long as wide; occipital carina narrow, not lamelliform, straight and entirely black medio-dorsally (Fig. 265); OOL 1.3 times as long as diameter of posterior ocellus; face narrow (Fig. 269); minimum width of malar space 0.3 times as long as second antennal segment (Fig. 265); clypeus medially flat, medio-ventrally semi-circularly emarginate, without depression medio-ventrally, its lateral corners protruding forwards; eye glabrous.

*Mesosoma*. Length of mesosoma 1.8 times its height; pronotal side rather low, ventrally sparsely finely punctate and dorsally densely and very finely punctulate, with a small acute antero-lateral protuberance (Fig. 266); mesoscutum slightly protruding anteriorly; propleuron slender (Fig. 266), 1.4 times as long as mesoscutum in front of tegulae; antesternal carina narrow and narrowly lamelliform; mesopleuron and metapleuron largely moderately regularly reticulate; mesoscutum with satin sheen, densely and finely punctulate and medio-posteriorly transversely rugose (Fig. 267); scutellum densely and finely punctulate; propodeum spaced reticulate.

*Wings*. Fore wing: first discal cell absent (Fig. 271), area glabrous; vein SR1 distinctly bent.

*Legs*. Hind coxa distinctly transversely rugose and with satin sheen dorsally, but laterally mainly punctulate; length of hind femur, tibia and basitarsus 5.4, 6.2 and 7.7 times their width, respectively (Fig. 268); middle tarsus 1.1 times as long as middle tibia; middle femur parallel-sided and slenderer than fore femur; hind femur slightly curved dorsally in lateral view.

*Metasoma*. Ovipositor sheath 1.2 times as long as body, 5.5 times as long as hind tibia and 1.7 times as long as metasoma; ivory part of ovipositor sheath 3.4 times as long as hind basitarsus; hypopygium slit-shaped incised apically.

*Colour*. Black-brown or dark brown; mandible (except dark teeth), palpi, tegulae, clypeus ventrally, fore and middle legs (but middle femur largely darkened, middle tibia dark brown medially, fore and middle tibiae basally and apically, 3 basal segments of fore tarsus and most of middle basitarsus ivory); subbasal patch of hind tibia, hind basitarsus (but basally dark brown) and second hind tarsal segment (but basally and apically with small dark brown patch) and apex of ovipositor sheath ivory; scape and antenna ventrally (except basally and apical segment) brown; wing membrane subhyaline; pterostigma dark brown.

*Male*. According to the original description similar to the female; third antennal segment 1.7 times second segment and fourth segment 1.8 times as long as third segment.

*Variation*. Body length 12.5–15.0 mm; propleuron 1.2–1.4 times as long as mesoscutum in front of tegulae; ovipositor sheath 1.0–1.2 times as long as body; ivory part of ovipositor sheath 3.0–3.4 times hind basitarsus; specimen from Hainan has also third hind tarsal segment ivory.

#### Distribution.

China (Hainan); Malaysia (Sabah).

#### Biology.

Unknown. Collected in June–July in China.

### Gasteruption terebrelligerum

Enderlein, 1913

http://species-id.net/wiki/Gasteruption_terebrelligerum

Figs 273 285

Gasteruption terebrelligerum Enderlein, 1913: 324; Hedicke 1939: 28; Pasteels 1958: 189.

#### Type material.

Holotype of *Gasteruption terebrelligerum*, ♀ (DEI), “[China:] Formosa [= Taiwan], Hoozan, 1910, H. Sauter”, “7.IX.”, “Holotypus”, “*Gasteruption terebrelligerum* Enderl., ♀, Type, Dr. Enderlein, det. 1913”, “Dtsch. Entomol. Institut Berlin”.

#### Additional material. 

Numerous specimens fromChina (Fujian, Mt. Wuyi, 1000 m (CSCS); Shanxi, Gamquan, Qingquanyou (CSCS); 1 ♀ + 3 ♂, Hubei, Huangmei (SCAU); Hunan, Shimen (CSCS); Yunnan, Baoshan, Xianyangqu, Lujiang (CSCS)).

#### Diagnosis.

**Figures 273–280.**
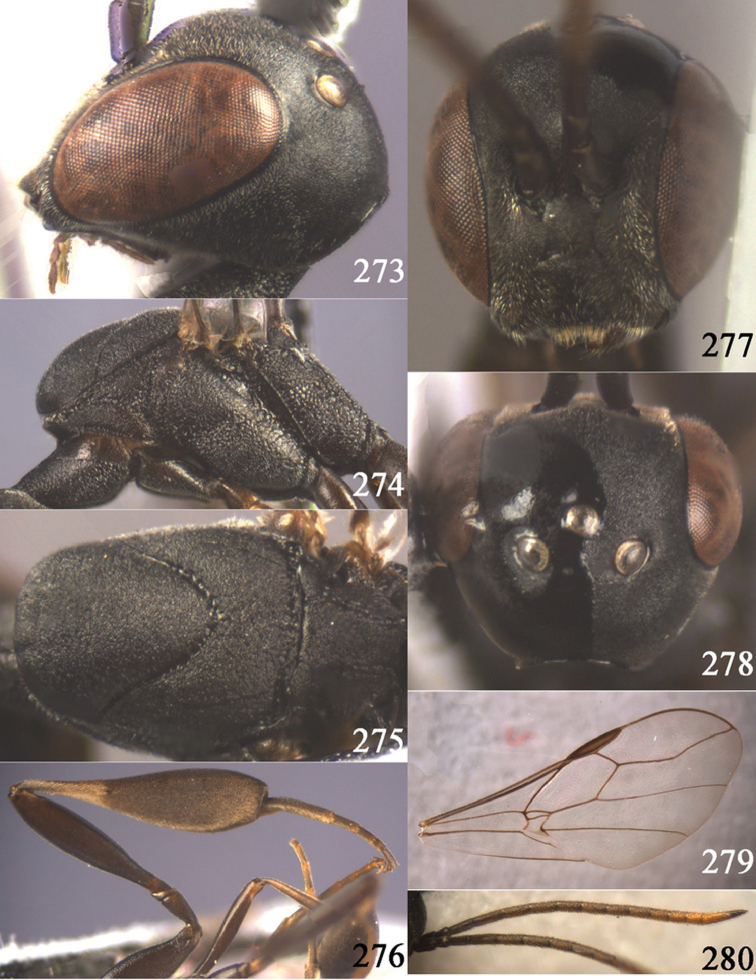
*Gasteruption terebrelligerum* Enderlein, 1913, female, Hubei. **273** head lateral **274** mesosoma lateral **275** mesoscutum dorsal **276** hind leg **277** head anterior **278** head dorsal **279** fore wing **280** antenna.

**Figures 281–285.**
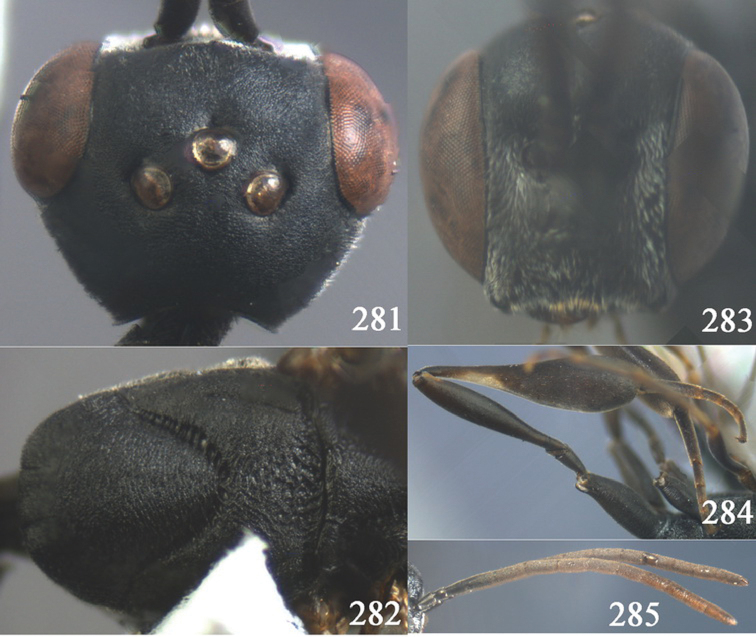
*Gasteruption terebrelligerum* Enderlein, 1913, male, Hubei. **281** head dorsal **282** mesoscutum dorsal **283** head anterior **284** hind leg **285** antennae.

Apex of ovipositor sheath dark brown; ovipositor sheath about 1.5 times as long as hind tibia and about 0.9 times as long as hind tibia and tarsus combined; occipital carina obsolescent medio-dorsally (Figs 273, 281) and slightly protruding ventro-posteriorly (Figs 273, 281); antesternal carina narrow; head, laterally mesosoma and scape black; head in anterior view slightly protruding below lower level of eyes by less than basal width of mandible and mandibular condylus near lower level of eyes (Figs 277, 283); in lateral viewcondylarincision of malar space close to eye (Figs 273, 281); clypeus with small depression or depression obsolescent; eyes setose; fourth and fifth antennal segment 1.1–1.2 and 1.0 (♀) –1.3 (♂) times as long as third segment, respectively (Figs 280, 285); apical antennal segment 1.2–1.4 times as long as third antennal segment and its colour similar to colour of medial segments; antenna of female may be partly or largely yellow-brown apically; mesoscutum and head dissimilarly sculptured, head very finely sculptured and matt, mesoscutum densely rugulose and more or less matt (Figs 275, 282); propleuron robust and 0.7–0.9 times as long as mesoscutum in front of tegulae (Fig. 274); hind coxa often transversely rugose or rugulose dorsally; hind tibia robust, as long as hind femur and trochanter combined, with a distinct subbasal ivory patch and swollen, resulting in a distinctly convex ventral border (Figs 276, 284); hind basitarsus comparatively long and parallel-sided (Figs 276, 284); hind tibial spurs dark brown or yellow-brown; hind tarsus dark brown; apical seventh of hypopygium incised.

#### Description.

Described after a female from Hubei (Huangmei), body length 9.0 mm, of fore wing 4.5 mm.

*Head*. Head directly narrowed behind eyes and weakly curved laterally (Fig. 278); temple 0.6 times as long as eye in dorsal view; vertex and frons matt and with finely aciculate; vertex rounded posteriorly and without depression medio-posteriorly; occipital carina narrow and non-lamelliform medio-dorsally (Fig. 273); third antennal segment 1.9 times as long as second segment, fourth antennal segment 1.2 times as long as third segment, fifth antennal segment as long as third segment (Fig. 280); eye shortly setose; OOL as long as diameter of posterior ocellus; minimum width of malar space 0.3 times as long as second antennal segment; clypeus without depression.

*Mesosoma*. Length of mesosoma twice as long as its height; propleuron robust, 0.7 times as long as mesoscutum in front of tegulae (Fig. 274), anterior of propleuron rather finely rugulose, posteriorly coriaceous and matt; side of pronotum mainly coriaceous, with a few rugulae; mesoscutum matt and coriaceous, medio-posteriorly with a few fine rugae (Fig. 275); scutellum coriaceous; propodeum rugose, medio-longitudinal carina distinct.

*Wings*. Fore wing: first discal cell subtriangular, m-cu rather short (Fig. 279).

*Legs*. Hind coxa moderately slender and dorsally rather finely rugulose; length of hind femur, tibia and basitarsus 4.3, 4.0 and 5.3 times their width, respectively (Fig. 276); hind tibia 2.0 mm.

*Metasoma*. Ovipositor sheath 0.2–0.3 times as long as body, 0.4–0.5 times as long as metasoma, about 0.9 times as long as hind tibia and tarsus combined and 1.5 times as long as hind tibia; hypopygium v-shaped incised apically.

*Colour*. Black; antenna dark brown; legs dark brown, but ventral patch of hind tibia ivory; ventral of metasoma dark brown; ovipositor sheath entire black.

*Male*. Body length 11.5 mm; temple 0.6 times as long as eye in dorsal view (Fig. 281); third antennal segment 1.5 times as long as second segment, fourth antennal segment 1.5 times as long as third segment, fifth antennal segment 1.3 times as long as third segment (Fig. 285); OOL as long as diameter of posterior ocellus; minimum width of malar space 0.2 times as long as second antennal segment; length of mesosoma twice its height; propleuron 0.8 times as long as mesoscutum in front of tegulae; side of pronotum mainly rather finely rugulose or rugose; mesoscutum matt and with finely strigate, medio-posteriorly rugose (Fig. 282); 2-CU1 vein of fore wing sinuate; length of hind femur, tibia and basitarsus 4.7, 3.9 and 4.8 times their width, respectively (Fig. 284); middle tarsus 1.2 times as long as middle tibia; dorsal subbasal patch of fore and middle tibiae and ventral subbasal patch of hind tibia ivory.

*Variation*. Female: body length 8–14 mm; temple 0.6–0.7 times as long as eye in dorsal view. Male: body length 8–11 mm, very similar to female.

#### Distribution.

China (Shanxi, Fujian, Taiwan, Hubei, Hunan, Yunnan).

#### Biology.

Unknown. Collected in May–July and September.

#### Notes.

Very similar to*Gasteruption brevicuspis* Kieffer, 1911, from India (Assam), but *Gasteruption brevicuspis* has the length of the mesosoma about 1.6 times its height, the head slightly slenderer in dorsal view, the ovipositor sheath 0.9 times as long as hind tibia and 0.6 times as long as hind tibia and tarsus combined and the mesoscutum somewhat stronger sculptured. The first discal cell of fore wing is comparatively narrow in the holotype of *Gasteruption terebrelligerum* (Fig. 348), but this is a rather variable character; it varies even among series of other species collected at the same day and locality.

### Gasteruption tonkinense

Pasteels, 1958

http://species-id.net/wiki/Gasteruption_tonkinense

Figs 286–294

Gasteruption tonkinense Pasteels, 1958: 180.

#### Type material.

Holotype, ♀ (MNHN), “[N. Vietnam], Tonkin, reg. de Hoa Bin, A. de Cooman, 1928”, “Holotype”, “*Gasteruption tonkinense* n. sp., J. Pasteels det., 1955”.

#### Additional material.

1 ♀ (TARI), “Chine, Prov. Kiangsu [=Jiangsu], Shanghai, Musée Heude”, “Zi-ka-wei [=Xujiahui], 9.VI.[19]20”, “O. Piel”; 1 ♀ (CSCS), “[China:] Zhejiang, Wuyanlong, Taishun, 28.VII.2005, N27°42’, E119°40’, alt. 1000 m, Yi-ping Wang”; 1 ♀ (ZJUH), “[China:] Zhejiang, Anji, Mt. Longwang, 24.VI.1996, Yun Ma”; 1 ♀ (ZJUH), “[China:] Zhejiang, Qingyuan, Mt. Baishanzu, 31.VIII.1993, Hong Wu”; 1 ♀ (ZJUH), “[China:] Zhejiang, Songyang, 18–31.VII.1989, Jun-hua He”; 2 ♀ (ZJUH), “[China:] Fujian, Jiangle, Mt. Longqi, 15.VII.1991 & 16.VII.1991, Chang-ming Liu”; 1 ♀ (SCAU), “[China:] Guangxi, Guilin, Mt. Maoer, 2–10.VIII.2005, Bin Xiao”; 1 ♀ (CSCS), “[China:] Guizhou, Sunchahe, Xishui, 24–28.IX.2000, alt. 800 m, Wei Xiao”.

#### Diagnosis.

**Figures 286–294.**
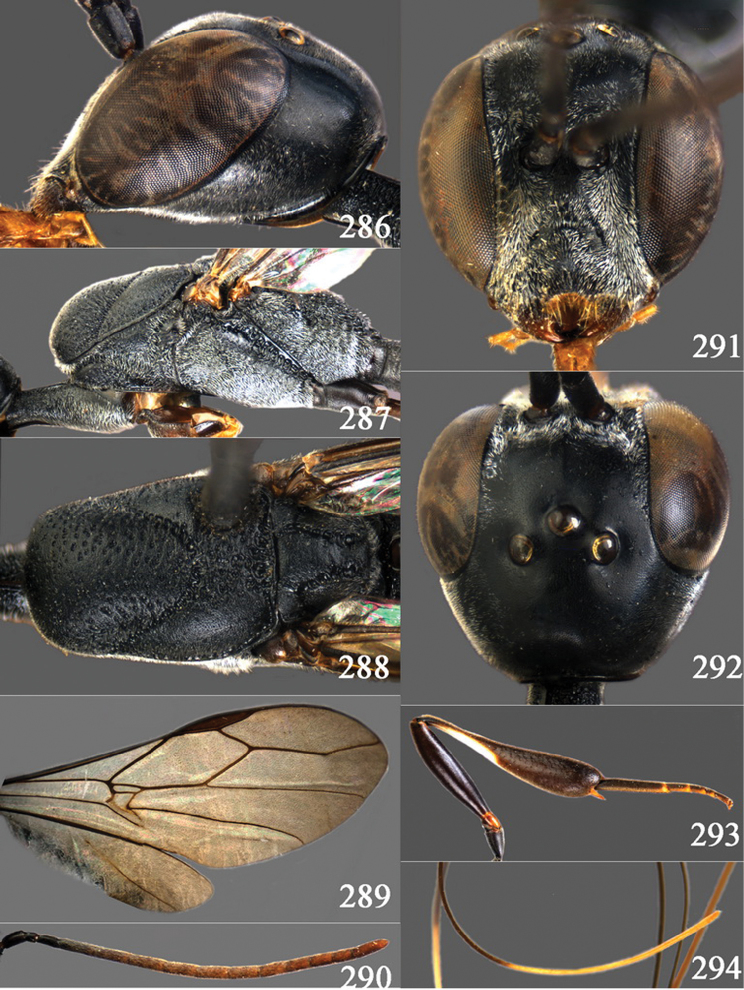
*Gasteruption tonkinense* Pasteels, 1958, female, Zhejiang. **286** head lateral **287** mesosoma lateral **288** mesoscutum dorsal **289** fore and hind wings **290** antenna **291** head anterior **292** head dorsal **293** hind leg **294** apex of ovipositor sheath.

Ivory apex of ovipositor sheath (Fig. 294) 3.0–3.5 times as long as hind basitarsus; ovipositor sheath 0.9–1.1 as long as body; occipital carina moderately lamelliform, upcurved and partly transparent medio-dorsally (Fig. 286) and rather protruding ventro-posteriorly (Fig. 286); head dorsally very finely punctulate and rather shiny, medio-posteriorly slightly flattened; propleuron 0.9–1.1 times as long as mesoscutum in front of tegulae (Fig. 287); antesternal carina narrow; head, laterally mesosoma and scape black; head in anterior view protruding below lower level of eyes by less than basal width of mandible and mandibular condylus near lower level of eyes (Fig. 291); in lateral viewcondylarincision of malar space almost touching eye (Fig. 286); clypeus without depression and lateral corners protruding forwards; eyes glabrous; fourth and fifth antennal segments 1.7–1.9 times and as long as third segment (specimen from China 1.5–1.7 times), respectively (Fig. 290); apical antennal segment of ♀ 1.5 times as long as third antennal segment and its colour similar to colour of medial segments; antenna of female dark brown; mesoscutum with satin sheen and coriaceous with spaced large punctures, but middle lobe laterally with some rugae and lateral lobe mainly coriaceous; hind coxa coarsely transversely rugose and shiny dorsally, but laterally mainly coriaceous and with satin sheen; hind tibia with a distinct subbasal ivory ring and rather swollen, resulting in a moderately convex ventral border (Fig. 293); hind basitarsus comparatively long (Fig. 293); hind tibial spurs rather dark brown; hind tarsus (including basitarsus) dark brown; apical 0.25 of hypopygium incised.

#### Description.

Holotype, female, body length 17.3 mm, of fore wing 8.9 mm.

*Head*. Vertex and frons shiny and rather sparsely very finely punctulate, moderately convex and without a distinct depression medio-posteriorly, but slightly flattened (Fig. 286); head directly narrowed behind eyes; temple 0.6 times as long as eye in dorsal view (Fig. 292); fourth antennal segment 1.8 times as long as third segment and as long as second and third segments combined, fifth antennal segment twice as long as third segment (Fig. 290), third antennal segment 1.2 times as long as second segment (Fig. 290); occipital carina moderately lamelliform, upcurved and partly transparent medio-dorsally; OOL 1.2 times as long as diameter of posterior ocellus; face moderately wide (Fig. 291); minimum width of malar space 0.05 times as long as second antennal segment (Fig. 286); clypeus without distinct depression, its lateral corners protruding forwards and medio-ventrally moderately emarginate; eye glabrous.

*Mesosoma*. Length of mesosoma 1.8 times its height; pronotal side moderately low and ventrally reticulate-rugose and dorsally finely coriaceous-punctulate, with a comparatively large antero-lateral tooth (Fig. 287); mesoscutum not protruding anteriorly; propleuron comparatively robust (Fig. 287), 0.9 times as long as mesoscutum in front of tegulae; antesternal carina narrow and narrowly lamelliform; mesopleuron largely coarsely reticulate-rugose; metapleuron regularly reticulate; mesoscutum with satin sheen and coriaceous with spaced large punctures, but middle lobe laterally with coarsely rugose and lateral lobe mainly coriaceous, medio-posteriorly coarsely reticulate or punctate (Fig. 288); scutellum largely coriaceous and with some punctures.

*Wings*. Fore wing: first discal cell nearly parallel-sided and with outer posterior corner rounded (Fig. 289), glabrous; vein SR1 distinctly bent.

*Legs*. Hind coxa coarsely transversely rugose and shiny dorsally, but laterally mainly coriaceous and with satin sheen; length of hind femur, tibia and basitarsus 4.3, 4.8 and 5.8 times their width, respectively (Fig. 293); middle tarsus 1.3 times as long as middle tibia; middle femur subparallel-sided and slenderer than fore femur; hind femur slightly curved dorsally.

*Metasoma*. Ovipositor sheath 0.9 times as long as body, 5.2 times hind tibia and 1.4 times metasoma; ivory apical part of sheath 3.2 times as long as hind basitarsus; hypopygium moderately slit-shaped incised apically.

*Colour*. Black or black-brown; fore and middle femora narrowly apically, fore and middle tibiae basally, apex of fore tibia, fore basitarsus, basal 0.6 of middle basitarsus, large subbasal patch of hind tibia (ventrally wider than dorsally) and apex of ovipositor sheath broadly ivory; palpi brown; mandible largely, antenna, tegulae and remainder of legs largely dark brown, but hind tibial spurs brown; wing membrane slightly infuscate; pterostigma dark brown.

*Male*. Unknown.

*Variation*. Body length 12–17 mm, of ovipositor sheath 0.9–1.1 times as long as its body, 1.4–1.6 times as long as metasoma and 4.9–5.3 times as long as hind tibia; ivory apical part of sheath 3.0–3.5 times as long as hind basitarsus. Temple about 0.5 times as long as eyes in dorsal view; third antennal segment 1.2–1.7 times as long as second segment, fourth antennal segment 1.7–1.9 times as long as third segment, fifth antennal segment 1.5–1.8 times as long as third segment; OOL 1.0–1.3 times as long as diameter of posterior ocellus; length of mesosoma 1.9–2.1 times as long as its height; length of propleuron 0.9–1.1 times as long as mesoscutum in front of tegulae; length of hind femur, tibia and basitarsus 4.0–4.5, 4.8–5.4 and 6.9–7.2 times their width, respectively.

#### Distribution.

Vietnam; China (Shanghai, Zhejiang, Fujian, Guangxi, Guizhou).

#### Biology.

Unknown. Collected in June–September.

#### Notes.

Close to *Gasteruption japonicum*, but this species has the vertex comparatively flat (distinctly convex in *Gasteruption tonkinense*), the hind basitarsus partly ivory (entirely dark brown), the side of the middle lobe of the mesoscutum sparsely finely punctate (coarsely punctate).

### Gasteruption tournieri

Schletterer, 1885

http://species-id.net/wiki/Gasteruption_tournieri

Figs 295 308

Foenus jaculator ; Tournier, 1887: viii.
Faenus jaculator ; Abeille de Perrin 1879: 263, 265, 270.
Gasteruption tournieri Schletterer, 1885: 287, 1889: 382, 388, 394, 395, 415; Dalla Torre 1902: 1075; Szépligeti 1903: 368; Kieffer 1912: 247; Schmiedeknecht, 1930: 376, 380; Hedicke 1939: 25; Ferrière, 1946: 237, 238, 246; Crosskey 1951: 292; Šedivý 1958: 35, 36, 42; Györfi and Bajári, 1962: 45, 49; Schmidt, 1969: 295; Dolfuss, 1982: 24; Oehlke 1984: 168, 171, 181; Madl 1987d: 404, 1987b: 24, 1988: 39, 1989a: 161, 1989b: 45, 1990a: 129, 1990b: 480, 483; Kozlov 1988: 246, 247; Kofler and Madl 1990: 322; Wall 1994: 164; Scaramozzino, 1995: 3; Pagliano and Scaramozzino 2000: 15, 17, 33; Saure, 2001: 29; Turrisi 2004: 84; Wisniowski 2004: 118; Yildirim et al. 2004: 1351; van der Smissen, 2010: 373.
Gasteruption austriacum Schletterer, 1885: 277; Hedicke 1939: 26; Wall 1994: 148. Synonymized with *Gasteruption tournieri* Schletterer by Schletterer 1889.
Gasteruption nitidum Schletterer, 1885: 281; Hedicke 1939: 26; Wall 1994: 149. Synonymized with *Gasteruption tournieri* Schletterer by Schletterer 1889.

#### Type material.

Type series of *Gasteruption tournieri* from Switzerland [Peney near Geneva], France [Bordeaux, Pérez], and Italy (Geneva Museum). Holotype of *Gasteruption austriacum* (a male in NMW from Austria, Frankenfeld and collected by Erber) and type series of *Gasteruption nitidum* (males in NMW from Italy, Calabria, collected by Erber) are probably lost.

#### Additional material.

1 ♀, “[China:] Jilin, Daxinggou, 7.VIII.2005”; 1 ♀ (CSCU), “[China:] Henan, Mt. Huangbai, Shangcheng, 200 m, 11.VII.1999, Mei-cai Wei”; 1 ♀ (ZJUH), “[China:] Henan, Neixiang, Baotianman Nature Reserve, 13–15.VII.1998, Yun Ma”; 4 ♀ (SCAU), “[China:] Hunan, Mt. Huping, 11–13.VII.2009, Shi-hong Wang”; 3 ♀ + 1 ♂ (ZJUH), “[China:] Hunan, Shimen, Mt. Huping, 11.VII.2009, Jie Zeng”; 1 ♀ (ZJUH), “[China:] Hunan, Mt. Huping, 13.VII.2009, Pu Tang”; 1 ♀ (ZJUH), “[China:] Guizhou, Huishui, VII.1987, Ji-ming Chu”; 1 ♀ (ZJUH), “[China:] Guizhou, Mayang River, 2.X.2007, Cui-hong Xie”.

#### Diagnosis.

Apex of ovipositor sheath with a distinct white or ivory band (Fig. 303), 1.7–2.6 times as long as hind basitarsus; head with middle depression in front of occipital carina comparatively deep and large, with both lateral depressions in front of occipital carina distinctly impressed (Figs 300, 304); occipital carina distinctly lamelliform and medium-sized to wide, more or less concave medio-dorsally (Figs 300, 304); antesternal carina narrow and non-lamelliform or nearly so, not or slightly elevated above mesosternum (Fig. 296); fourth and fifth antennal segments of female 1.5–2.1 and 1.1–2.0 times as long as third segment, respectively (Fig. 302); frons sparsely punctulate and with distinct interspaces or very finely and densely punctulate; vertex more or less finely punctulate and distinctly shiny; face comparatively wide (Figs 299, 306); temples linearly narrowed behind eyes (Figs 300, 304) and shorter than eyes; propleuron 0.8–1.1 times mesoscutum up to tegulae (Fig. 296); lateral lobes of mesoscutum rugulose-coriaceous; anterior half of mesoscutum moderately punctate-rugose (Figs 297, 305); subbasally outer side of hind tibia and apical half of hind basitarsus ivory; fore and middle legs variable from white, ivory to brown; body slender; ovipositor sheath 1.0–1.2 times as long as body, 1.4–1.8 times as long as metasoma and 4.9–5.7 times as long as hind tibia; hypopygium deep slit-shaped incised apically.

#### Description.

Described from a female from Hunan, body length 18.5 mm.

**Figures 295–303.**
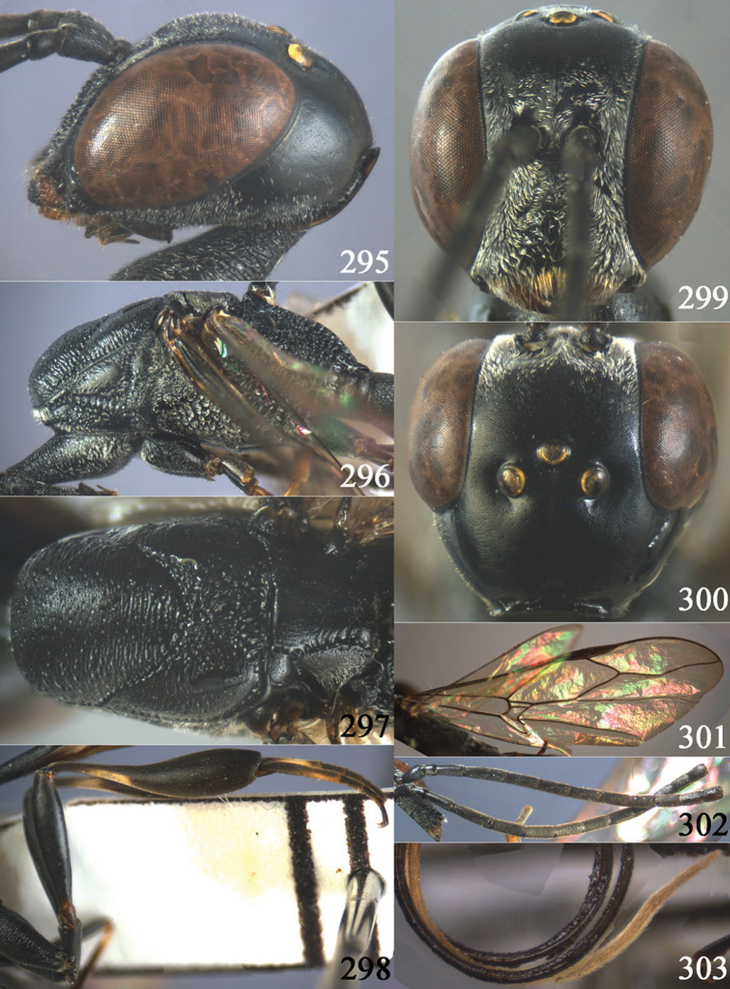
*Gasteruption tournieri* Schletterer, 1885, female, Hunan. **295** head lateral **296** mesosoma lateral **297** mesoscutum dorsal **298** hind leg **299** head anterior **300** head dorsal **301** fore wing **302** antennae **303** apex of ovipositor sheath.

*Head*. Head directly narrowed behind eyes; temple 0.5 times as long as eye in dorsal view (Fig. 300); vertex and frons with satin sheen and sparsely finely punctulated; vertex moderately convex posteriorly (Fig. 295) and with a large depression medio-posteriorly, lateral depressions distinct (Fig. 300); third antennal segment 1.8 times as long as second segment, fourth antennal segment 1.5 times as long as third segment, fifth antennal segment 1.2 times as long as third segment (Fig. 302); eye shortly setose; OOL 1.3 times as long as diameter of posterior ocellus; minimum width of malar space 0.2 times as long as second antennal segment; clypeus without depression.

*Mesosoma*. Length of mesosoma 2.1 times as long as its height; propleuron comparatively slender and densely rugose, 0.9 times as long as mesoscutum in front of tegulae (Fig. 296); side of pronotum mainly coriaceous, but ventrally rugose, antero-lateral tooth distinctly; mesoscutum mainly transversely rugose, with satin sheen and sparsely punctured, medio-posteriorly with large and more or less coalescent punctures (Fig. 297); scutellum shiny, coriaceous to finely transversely rugulose; propodeum shiny and reticulate-rugose, medio-longitudinal carina absent.

*Wings*. Fore wing: first discal cell parallel-sided and with outer posterior corner rounded. (Fig. 301)

*Legs*. Hind coxa matt and transversely rugose dorsally; length of hind femur, tibia and basitarsus 4.2, 4.9 and 6.8 times their width, respectively (Fig. 298).

*Metasoma*. Ovipositor sheath about as long as body, 1.4 times as long as metasoma and 5.0 times as long as hind tibia; its apical ivory part 0.1 times as long as ovipositor sheath and 2.1 times as long as hind basitarsus; hypopygium slit-shaped incised apically.

*Colour*. Black; fore and middle tibia brown; ventral of hind tibia with a large white patch; basitarsus with a basal yellow-brown patch; second and third metasomal tergites apically red-brown.

**Figures 304–308.**
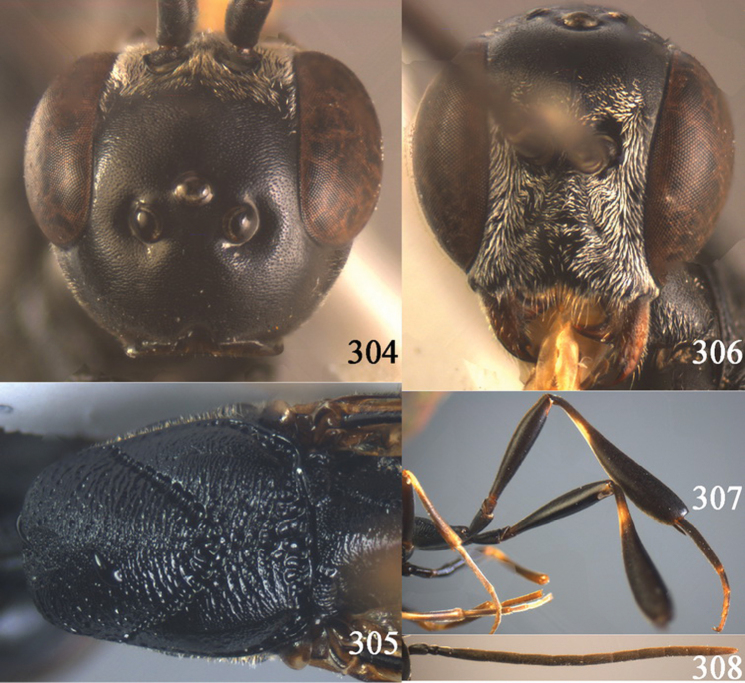
*Gasteruption tournieri* Schletterer, 1885, male, Hunan. **304** head dorsal **305 **mesoscutum dorsal **306** head anterior **307** hind leg **308** antenna.

*Male* (described after a male from Hunan). Body length 10.5 mm; vertex with three depressions in front of occipital carina, medio-dorsal one medium-sized and both of lateral ones comparatively shallow (Fig. 304); temple 0.5–0.6 times as long as eye in dorsal view; third antennal segment distinctly short, 1.4 times as long as second segment, fourth antennal segment 2.6 times as long as third segment, fifth antennal segment 2.5 times as long as third segment (Fig. 308); OOL as long as diameter of posterior ocellus; minimum width of malar space 0.2 times as long as second antennal segment; length of mesosoma twice its height; propleuron with satin sheen and reticulate-rugose, 0.8 times as long as mesoscutum in front of tegulae; dorsal lobe of pronotum coriaceous and rugulose all around, ventrally with satin sheen and rugose or reticulate-rugose, posteriorly moderately smooth, dorso-laterally coriaceous and ventro-laterally rugose, with a distinct antero-lateral tooth; mesoscutum transversely rugose or punctate-rugose, medio-posteriorly coarsely punctate-rugose, laterally rugose (Fig. 305), scutellum mainly coriaceous but finely and broadly transversely rugulose; propodeum coarsely reticulate-rugose, medio-longitudinal carina indistinct; hind coxa moderately slender and dorsally finely transversely rugulose; length of hind femur, tibia and basitarsus 5.1, 5.4 and 6.3 times their width, respectively (Fig. 307); middle tarsus 1.3 times as long as middle tibia; the colour of body black; fore and middle legs (except for coxa) mainly dark brown to brown, hind legs black-brown, basal patch of fore and middle tibia and ventrally subbasal patch of hind tibia ivory.

*Variation*. Chinese specimens: Body length 14.0–18.5 mm. Ovipositor sheath 1.0–1.2 times as long as body, 1.4–1.8 times as long as metasoma and 5.0–5.7 times as long as hind tibia, its apical ivory part 2.1–2.6 times as long as hind basitarsus; temple 0.4–0.6 times as long as eye in dorsal view; propleuron shiny and slender, 0.9–1.0 times as long as mesoscutum in front of tegulae.

#### Distribution.

China (Jilin, Henan, Hunan, Guizhou); Japan, South and Central Europe, up to England, Netherlands and Germany.

#### Biology.

Unknown. Collected in April–May in Japan, in July–August and October in China.

#### Notes.

The Chinese specimens have a slightly differently shaped head, but this may be part of a clinal variation. Specimens from the Central Palaearctic are necessary to decide if this is the case.

### Gasteruption transversiceps

Pasteels, 1958

http://species-id.net/wiki/Gasteruption_transversiceps

Figs 309–316

Gasteruption transversiceps Pasteels, 1958: 208–209, fig. 30.

#### Type material.

Lectotype here designated, ♀ (USNM), “[E. Malaysia: Sabah], Borneo, Sandakan, Baker”, “Paratype”, “*Gasteruption transversiceps* n. sp., J. Pasteels det., 1955”, “*Gasteruption transversiceps* Pasteels, Pasteels, 1955”.

#### Additional material.

1 ♀, (ZJUH), “[China:] Hainan, Mt. Wuzhi, 15–16.V.2008, Jing-xian Liu”.

#### Diagnosis.

**Figures 309–316.**
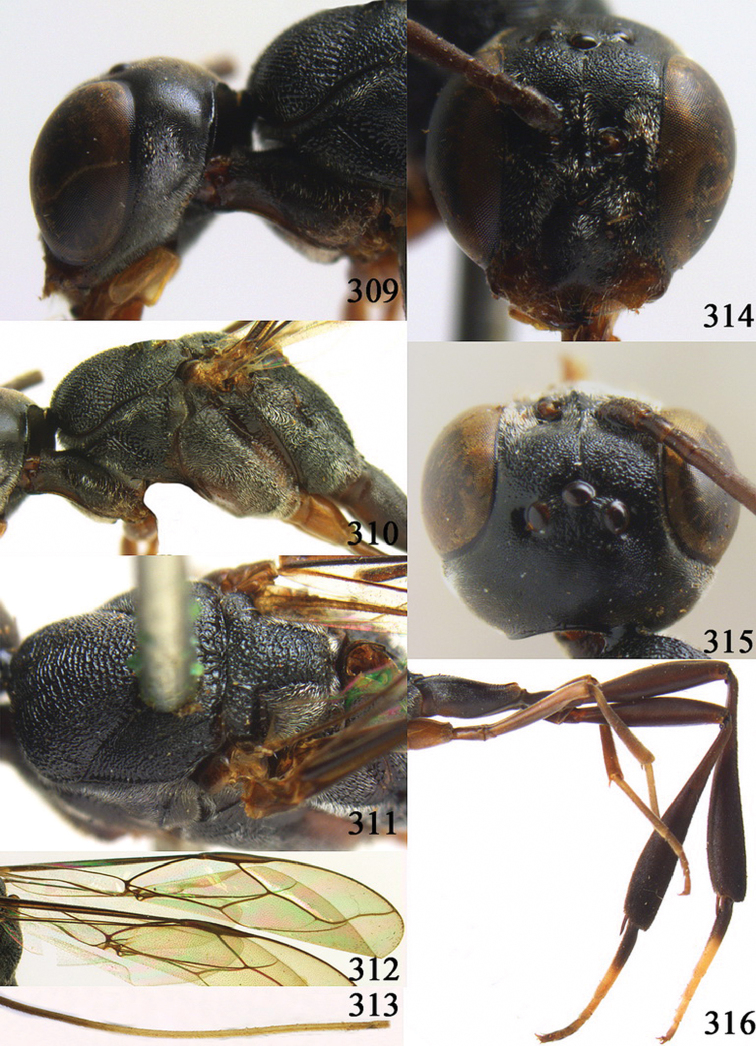
*Gasteruption transversiceps* Pasteels, 1958, lectotype, female. **309** head lateral **310** mesosoma lateral **311** mesoscutum dorsal **312** fore wing **313** apex of ovipositor sheath **314** head anterior **315** head dorsal **316** hind leg.

Apical third of ovipositor sheath ivory (Fig. 313); ovipositor sheath 1.8–2.0 times as long as body; occipital carina narrow and hardly or not lamelliform (Fig. 309); head transverse in dorsal view (Fig. 315); vertex slightly convex in front of occipital carina, rather shiny and densely finely punctate, without a medio-posterior depression; fourth antennal segment about 2.6 times as long as third segment and third segment 1.1 times as long as second segment; frons rugose; mesoscutum rugose; middle lobe of mesoscutum moderately protuberant (Fig. 311); hind tibia narrow (Fig. 316); hind basitarsus dark brown subbasally; ivory apical part of ovipositor sheath 5–8 times as long as hind basitarsus; hypopygium deep slit-shaped incised apically.

#### Description.

Lectotype, female, body length 14.4 mm, of fore wing 8.4 mm.

*Head*. Head comparatively transverse (Fig. 315); vertex rather shiny and densely finely punctate but nearly smooth near occipital carina, slightly convex and without a distinct depression medio-posteriorly; frons rather coarsely finely rugose and with distinct narrow groove in front of anterior ocellus (Fig. 315); head directly roundly narrowed behind eyes; temple 0.5 times as long as eye in dorsal view; fourth antennal segment 2.6 times as long as third segment and 1.4 times as long as second and third segments combined, fifth antennal segment 2.4 times as long as third segment, third antennal segment 1.1 times as long as second segment and 1.3 times as long as wide; occipital carina narrow, not lamelliform, straight and entirely black medio-dorsally (Fig. 309); OOL 1.4 times as long as diameter of posterior ocellus; face moderately wide (Fig. 314); minimum width of malar space 0.4 times as long as second antennal segment (Fig. 309); clypeus medially flat, medio-ventrally emarginate because of narrow triangular depression medio-ventrally, its lateral corners protruding forwards (Fig. 314); eye glabrous.

*Mesosoma*. Length of mesosoma 1.7 times its height; pronotal side high and ventrally reticulate-rugose and dorsally finely punctate but rugose posteriorly, with a small blunt antero-lateral protuberance (Fig. 310); mesoscutum not protruding anteriorly; propleuron robust (Fig. 310), 0.8 times as long as mesoscutum in front of tegulae; antesternal carina narrow and narrowly lamelliform; mesopleuron and metapleuron largely moderately regularly rugose-reticulate; mesoscutum with satin sheen, middle lobe densely and partly transversely rugose and lateral lobe densely rugulose-punctate, medio-posteriorly coarsely reticulate (Fig. 311); scutellum largely coarsely irregularly rugose.

*Wings*. Fore wing: first discal cell parallel-sided and with outer posterior corner rounded (Fig. 312), glabrous; vein SR1 distinctly sinuate.

*Legs*. Hind coxa superficially transversely rugose and shiny dorsally, but laterally mainly punctulate-coriaceous and with satin sheen; length of hind femur, tibia and basitarsus 5.1, 5.9 and 6.5 times their width, respectively (Fig. 316); middle tarsus 1.2 times as long as middle tibia; middle femur subparallel-sided and hardly slenderer than fore femur; hind femur slightly curved dorsally.

*Metasoma*. Ovipositor sheath about 1.8 times as long as body, about 7.9 times as long as hind tibia and about 2.7 times as long as metasoma; ivory part of ovipositor sheath incomplete, at least 3.5 times hind basitarsus; according to original description about 7.5 times as long as hind basitarsus (“posterior third of sheath ivory”); hypopygium slit-shaped incised apically.

*Colour*. Black or black-brown; mandible (except dark teeth), palpi, clypeus latero-ventrally, fore and middle legs (but middle femur somewhat darkened), hind coxa ventro-basally yellow-brown; hind tarsus (except basal half of basitarsus) and apex of ovipositor sheath broadly ivory; mesopleuron anteriorly and posteriorly and mesosternum largely brown; antenna, tegulae and remainder of legs largely dark brown; wing membrane subhyaline; pterostigma brown, but dark brown laterally.

*Male*. Unknown.

*Variation*. Chinese specimen: Body length 19 mm, ovipositor sheath twice as long as body, 2.8 times as long as metasoma, 5.3 times as long as hind tibia and tarsus combined and 9 times as long as hind tibia; its apical ivory part 6.7 times as long as hind basitarsus and 0.25 times length of ovipositor sheath.

#### Distribution.

Orental China (Hainan); East Malaysia (Sabah).

#### Biology.

Unknown. Collected in May.

#### Notes. 

The designation of a lectotype is necessary because in the original description it is not indicated which of the type specimens (three females from Sabah and one female from Luzon) is the holotype.

### Gasteruption varipes

(Westwood, 1851)

http://species-id.net/wiki/Gasteruption_varipes

Figs 317 333

Foenus varipes Westwood, 1851: 220.
Gasteruption varipes ; Schletterer, 1885: 289, 325, 1890: 383, 434; Kieffer 1912: 231, 269; Hedicke 1939: 26–27; Pasteels 1958: 186–188.

#### Type material.

Holotype of *Gasteruption varipes*, ♂ (BMNH), “Type, H.T.”, “China/45 65”, “B.M. Type Hym. 3.a.177”, “*varipes* Westw.”, “B.M. Type Hym. *Foenus varipes* Westwood, 1851”.

#### Additional material.

7 ♂ (ZJUH, RMNH), “[China:] Fujian, Fuzhou, 22.VIII.1988, Xiu-fu Zhao”; 1 ♂ (ZJUH), “[China:] Fujian, Fuzhou, 20.VI.1988, Sa-ping Yi”; 1 ♂ (ZJUH), “[China:] Fujian, Fuzhou, 1.VIII.1988, Xiu-fu Zhao”; 1 ♂ (ZJUH), “[China:] Fujian, Fuzhou, 2.VIII.1988”; 1 ♀ (ZJUH), “[China:] Fujian, Fuzhou, 3.VII.1990, Nai-quan Lin”; 1 ♂ (ZJUH), “[China:] Fujian, Fuzhou, 11.IX.1990, Chang-ming Liu”; 1 ♂ (ZJUH), “[China] Fujian, Fuzhou, Jinshan, 18.VII.1986, Nai-quan Lin”; 1 ♀ (ZJUH), “[China:] Fujian, Fuzhou, Jinshan, 6.VI.1984, Yu-hua Xia”; 1 ♀ + 1 ♂ (ZJUH), “[China:] Fujian, Fuzhou, Jinshan, 3–9.V.1990, Xiu-fu Zhao”; 1 ♀ (ZJUH), “[China:] Fujian, Fuzhou, Jinshan, 6.VI.1989, Xiu-fu Zhao”; 2 ♂ (ZJUH), “[China:] Fujian, Fuzhou, Jinshan, 20.VI.1990, Xiu-fu Zhao”; 1 ♀ (ZJUH), “[China:] Fujian, Fuzhou, Jinshan, 10–15.V.1990, Xiu-fu Zhao”; 1 ♀ (ZJUH), “[China:] Fujian, Fuzhou, Meifeng, 14.VII.1965, Wen-lan Xu”; 1 ♀ (RMNH), “[China:] Fujian, Shaxian, 20.VII.1980, Zhen-yong Mei”; 1 ♂ (ZJUH), “[China:] Fujian, Mt. Wuyi, 29.V.1990, Chang-ming Liu”; 1 ♂ (ZJUH), “[China:] Fujian, Jianyang, Chengguan, 23.VIII.1980”; 3 ♀ (ZJUH), “[China:] Fujian, Jiangle, Mt. Longqi, 1.VII.1991, Chang-ming Liu”; 1 ♀ (TARI), “[China] Taiwan, Airyo, Heito, 10.X.1926, J. Sonan”; 1 ♀ (CSCU), “[China:] Hainan, Mt. Jianfengling, 18.III.1999, Mei-cai Wei & Hai-yan Nie”; 1 ♂ (ZJUH), “[China:] Hainan, Baisha, Mt. Yinggeling, 1–2.V.2008, Jing-xian Liu”; 1 ♀ (ZJUH), “[China:] Hainan, Mt. Yinggeling, 23.V.2007, Jing-xian Liu”; 1 ♂ (ZJUH), “[China:] Hainan, Mt. Wuzhi, 15.V.2008, Jing-xian Liu”; 1 ♀ (ZJUH), “[China:] Yunnan, Jinggu, Weiyuan, 4.X.2004, Jing-xian Liu”.

#### Diagnosis.

**Figures 317–324.**
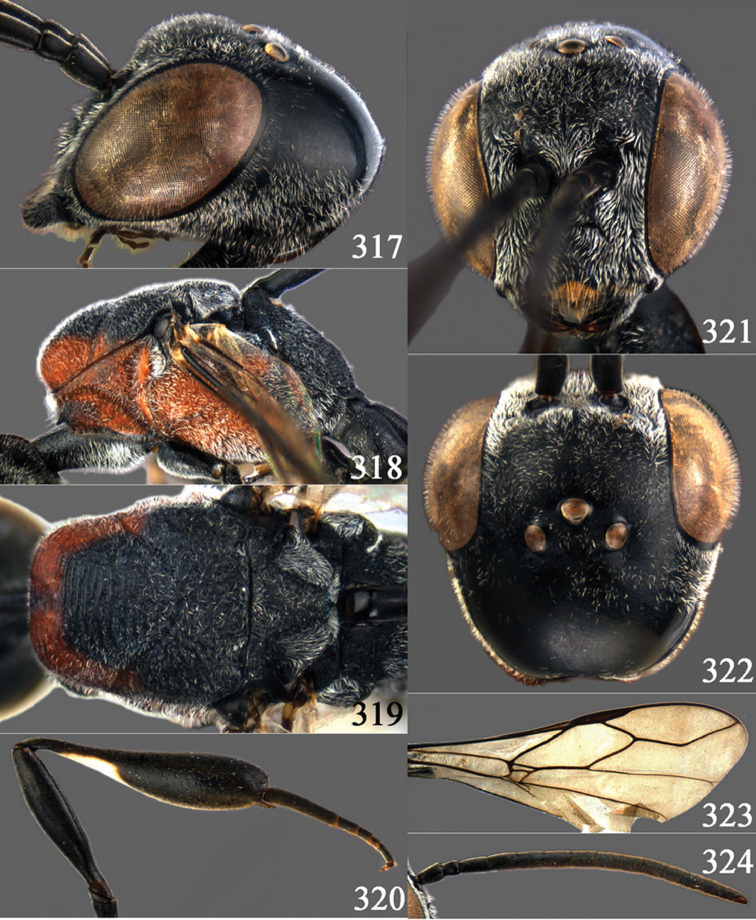
*Gasteruption varipes* (Westwood, 1851), female, Hainan. **317** head lateral **318** mesosoma lateral **319** mesoscutum dorsal **320** hind leg **321** head anterior **322** head dorsal **323**  fore wing **324** antenna.

**Figures 325–333.**
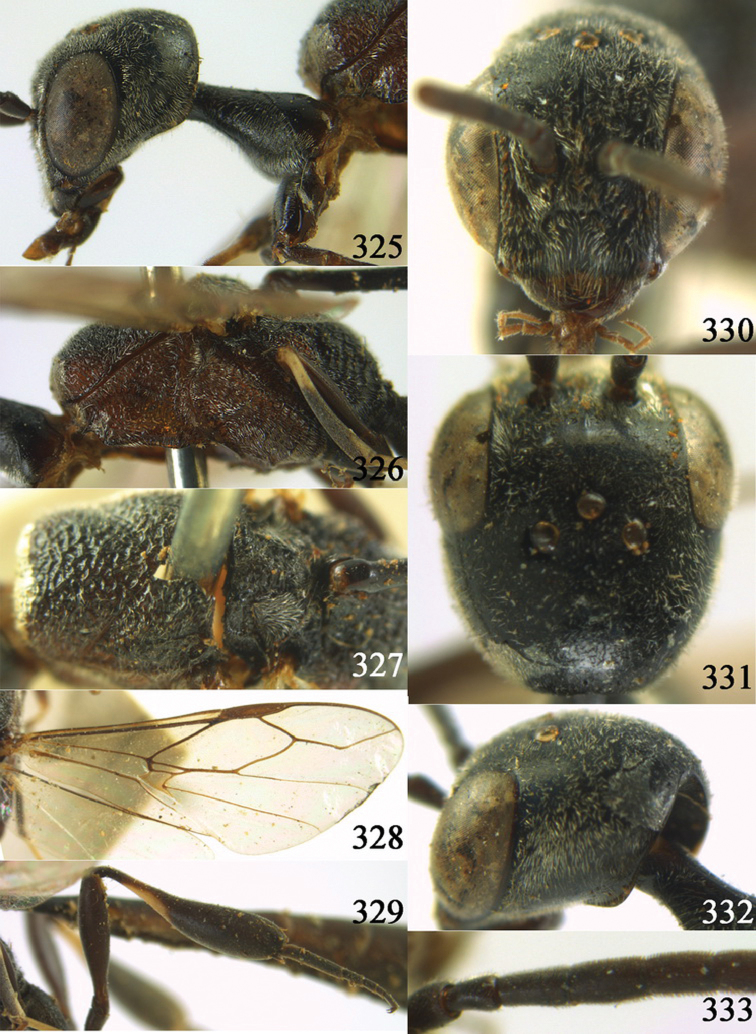
*Gasteruption varipes* (Westwood, 1851), holotype, male. **325** head lateral **326** mesosoma lateral **327** mesoscutum dorsal **328** fore wing **329** hind leg **330** head anterior **331** head dorsal **332** occipital carina dorsa-lateral **333** the second to fifth antennal segments.

Head without a depression in front of occipital carina; antesternal carina non-lamelliform and narrow (Figs 318, 326); propleuron 0.9–1.1 times as long as mesoscutum in front of tegulae and rather robust (Figs 318, 326); occipital carina obsolescent medio-dorsally (Figs 317, 325, 332); head roundly narrowed behind eyes in dorsal view (Figs 322, 331); temple longer than eye in dorsal view; third antennal segment of ♀ 1.2–1.5 times as long as second segment, fourth antennal segment 1.0–1.2 times as long as third segment, fifth antennal segment 0.9–1.1 times as long as third segment, of ♂ fourth segment 1.1 times as long as third segment and 0.6 times as long as second and third segments combined, fifth antennal segment as long as third segment (Figs 324, 333) and penultimate segments unknown; third antennal segment of ♂ normal, 1.5 times as long as second segment (Figs 324, 333); vertex largely smooth and with satin sheen; malar space short (Figs 317, 325); antero-lateral teeth of pronotum absent or indistinct; mesoscutum robust (Figs 319, 327) and very coarsely reticulate-rugose, without separate punctures; marginal cell of fore wing elongate (Figs 323, 328); hind coxa rather slender and transversely rugose; hind coxa black; pronotal side dark red-brown; hind basitarsus of male black-brown (but basally somewhat brown) and hind tibia bicoloured (dorsally dark brown, ventrally with large ivory subbasal patch (Fig. 329); female tibia unknown; apical half of paramere light brown; [ovipositor sheath 0.8–1.2 times as long as body, 1.4–1.7 times as long as metasoma and 4.0–6.0 times as long as hind tibia].

#### Description.

Holotype, male, body length 16.7 mm.

*Head*. Vertex with satin sheen and largely smooth, densely setose, distinctly convex and without depression medio-posteriorly; head gradually narrowed behind eyes; temple 1.1 times as long as eye in dorsal view (Fig. 331); fourth antennal segment 1.1 times as long as third segment and 0.6 times longer than second and third segments combined, fifth antennal segment as long as third segment (Fig. 333), third antennal segment long and 1.5 times as long as second segment; occipital carina non-lamelliform and obsolescent medio-dorsally (Fig. 325); ocelli small, OOL 1.8 times as long as diameter of posterior ocellus; face narrow (♀ Fig. 321, Fig. 330); malar space 0.2 times as long as second antennal segment (= pedicellus).

*Mesosoma*. Length of mesosoma 1.6 times its height; pronotal side normal and mainly reticulate-rugose, including ventrally; mesoscutum slightly protruding anteriorly; propleuron 1.1 times as long as mesoscutum in front of tegulae; antesternal carina narrow and hardly lamelliform; middle and lateral lobes of mesoscutum shiny and very coarsely reticulate-rugose, without separate punctures (Fig. 327); scutellum coarsely reticulate-rugose; propodeum coarsely reticulate-rugose, without median carina; entire mesopleuron coarsely reticulate-rugose.

*Wings*. First discal cell elongate triangular, slightly narrowed and no distal posterior corner (Fig. 328).

*Legs*. Hind coxa with satin sheen, coriaceous but transversely rugose dorsally; length of hind femur, tibia and basitarsus 4.2, 4.4 and 5.6 times their width, respectively (Fig. 329).

*Metasoma*. Apical half of paramere light brown.

*Colour*. Black or dark brown (including mandible); pronotal side, mesoscutum laterally and mesopleuron dark red-brown; base and apical patch of fore and middle tibiae, fore and middle basitarsi largely and ventrally hind tibia with large subbasal patch ivory; hind tibial spurs dark brown; pterostigma dark brown.

*Female* (described after a female from Yunnan, Jinggu). Body length 14.0 mm.

*Head*. Head at most slightly emarginate medio-posteriorly, gradually narrowed behind eyes and weakly curved laterally (Fig. 322); temple 0.8 times as long as eye in dorsal view; vertex and frons with satin sheen and coriaceous; vertex without depression medio-posteriorly; occipical carina non-lamelliform and obsolescent medio-dorsally (Fig. 317); third antennal segment 1.5 times as long as second segment, fourth antennal segment 1.1 times as long as third segment, fifth antennal segment 0.9 times as long as third segment (Fig. 324); eye setose; OOL 1.7 times as long as diameter of posterior ocellus; minimum width of malar space 0.2 times as long as second antennal segment; clypeus with indistinct triangular depression.

*Mesosoma*. Length of mesosoma 1.8 times as long as its height; propleuron with punctate and coriaceous, 1.1 times as long as mesoscutum in front of tegulae (Fig. 318); side of pronotum rugose and interspaces coriaceous, with a distinct medio-lateral tooth; mesopleuron coarsely vermiculate-rugose; mesoscutum with coarsely broadly transversely rugose, laterally and posteriorly coarsely reticulate-rugose (Fig. 319); scutellum rugulose; propodeum densely rugose, medio-longitudinal carina indistinct.

*Wings*. Fore wing: first discal cell parallel-sided and with outer posterior corner rounded (Fig. 323).

*Legs*. Hind coxa with satin sheen, moderately slender and dorsally transversely striae; length of hind femur, tibia and basitausus 4.1, 4.6 and 5.5 times as long as their width, respectively (Fig. 320); middle tarsus 1.1 times as long as middle tibia; hind tibia 3.0 mm.

*Metasoma*. Ovipositor sheath about 1.1 times as long as body, 1.7 times as long as metasoma and 5.0 times as long as hind tibia; hypopygium slit-shaped incised apically.

*Colour*. Black; mandible dark brown; pronotal side, mesoscutum antero-laterally and mesopleuron red-brown; fore and middle legs (except coxae black) dark brown to brown, but basal portion of tibiae pale, base of middle basitarsi ivory; hind legs (except coxae black) dark brown, subbasal portion of tibia pale; metasoma dark brown; ovipositor sheath entirely black.

*Variation*. Female: body length 14.0–15.5 mm, ovipositor sheath 0.8–1.2 times as long as body, 1.4–1.7 times as long as metasoma, 4.3–6.0 times as long as hind tibia; temple 0.8–1.0 times as long as eye in dorsal view; third antennal segment 1.2–1.5 times as long as second segment, fourth antennal segment 1.0–1.2 times as long as third segment, fifth antennal segment 0.9–1.1 times as long as third segment; length of mesosoma 1.8–1.9 times as long as its height; propleuron 0.9–1.1 times as long as mesoscutum in front of tegulae; mesoscutum coarsely reticulate-rugose; propodeum coarsely irregularly rugose, medio-longitudinal carina indistinct; mandible apically red-brown; pronotum, mesopleuron, mesosternum and apical of mesoscutum red-brown; metasoma black or dark brown, in some species second-third metasomal tergites red-brown.Male: body length 11.5–16.0 mm.

#### Distribution.

Oriental China (Fujian, Taiwan, Hainan, Yunnan). According to Pasteels (1958) *Gasteruption varipes* occurs also in South India, but this may be based on a misidentification.

#### Biology.

Unknown. Collected in March, May–October.

**Figures 334–341.**
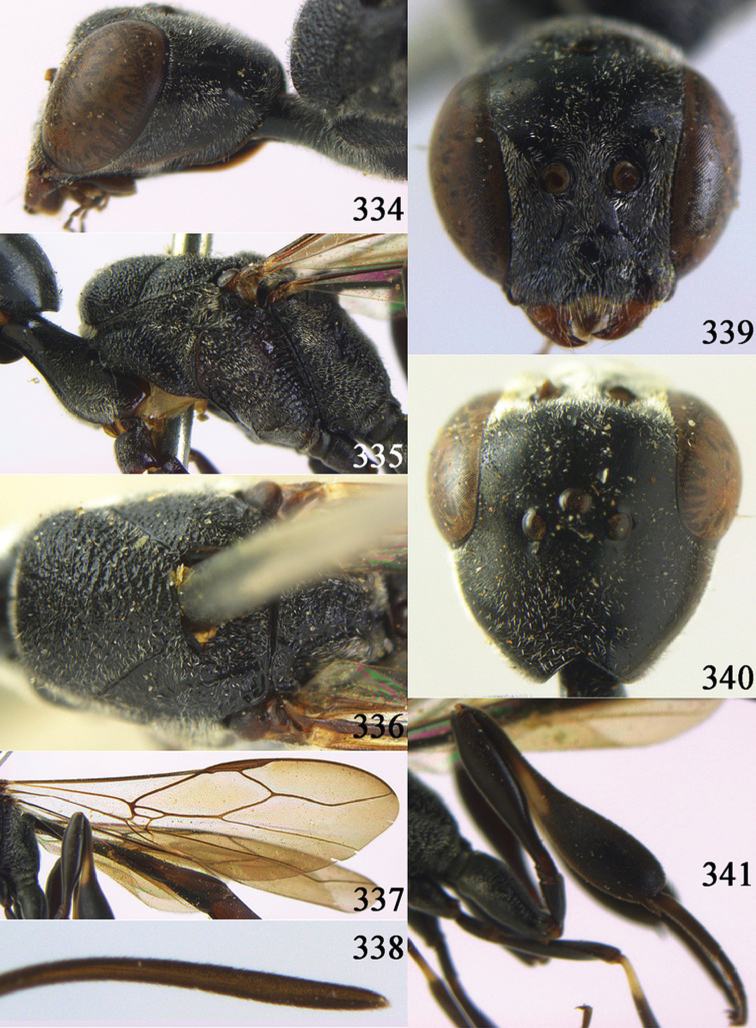
*Gasteruption obscuripenne* Pasteels, 1958, holotype, female. **334** head lateral **335** mesosoma lateral **336** mesoscutum dorsal **337** fore wing **338** apex of ovipositor sheath **339** head anterior **340** head dorsal **341** hind leg.

**Figures 342–349.**
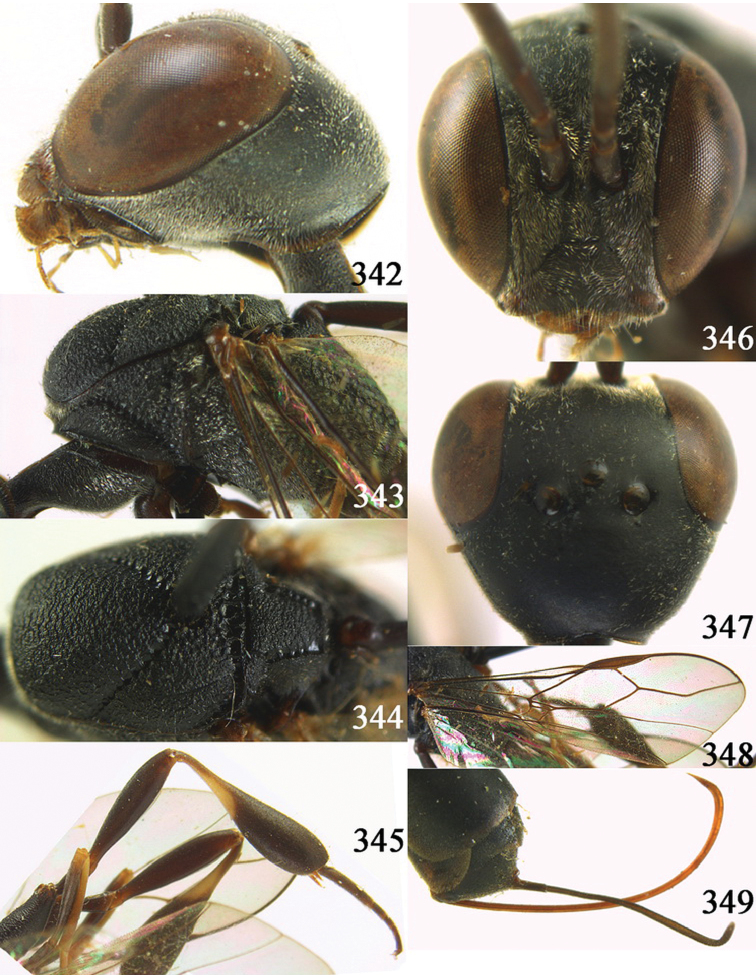
*Gasteruption terebrelligerum* Enderlein, 1913, holotype, female. **342** head lateral **343** mesosoma lateral **344** mesoscutum dorsal **345** hind leg **346** head anterior **347** head dorsal **348** fore wing **349** ovipositor and its sheath.

**Table 3.** List of species of *Gasteruption* recorded from China (valid species in bold).

*Gasteruption abeillei* Kieffer, 1912 => assectator
*Gasteruption affectator* auct. => *assectator*
***Gasteruption amoyense* Pasteels, 1958**
***Gasteruption angulatum* sp. n.**
*Gasteruption annularis* Geoffrey, 1785 => assectator
***Gasteruption assectator* (Linnaeus, 1758)**
***Gasteruption assectoides* sp. n.**
*Gasteruption austriacum* Schletterer, 1885 => tournieri
***Gasteruption bimaculatum* Pasteels, 1958**
***Gasteruption birmanense* Pasteels, 1958**
*Gasteruption borealis* Thomson, 1883 => assectator
*Gasteruption brevicauda* Kieffer, 1904 => assectator
*Gasteruption breviterebrae* Watanabe, 1934 => assectator
***Gasteruption coloratum* sp. n.**
***Gasteruption corniculigerum* Enderlein, 1913**
*Gasteruption curiosum* Pasteels, 1958 => amoyense syn. n.
***Gasteruption dilutum* Semenov, 1892**
***Gasteruption dimidiatum* Semenov, 1892**
***Gasteruption formilis* Alekseev, 1995**
***Gasteruption formosanum* Enderlein, 1913**
*Gasteruption fumipennis* Thomson, 1883 => assectator
***Gasteruption japonicum* Cameron, 1888**
***Gasteruption latitibia* sp. n.**
*Gasteruption margotae* Madl, 1987 => assectator
*Gasteruption micrura* Kieffer, 1904 => assectator
*Gasteruption nigripectus* Kieffer, 1904 => assectator
*Gasteruption nigritarsis* Thomson, 1883 => assectator
*Gasteruption nitidum* Schletterer, 1885 => tournieri
***Gasteruption oriplanum* Kieffer, 1911**
***Gasteruption parvicollarium* Enderlein, 1913**
***Gasteruption poecilothecum* Kieffer, 1911**
***Gasteruption rufescenticorne* Enderlein, 1913**
***Gasteruption sinarum* Kieffer, 1911**
*Gasteruption sinense* Kieffer, 1924 => sinarum syn. n.
*Gasteruption sinense* var. *minus* Kieffer, 1924 => japonicum syn. n.
***Gasteruption sinepunctatum* sp. n.**
***Gasteruption sinicola* Kieffer, 1924**
***Gasteruption strigosum* sp. n.**
***Gasteruption subhamatum* Pasteels, 1958**
***Gasteruption terebrelligerum* Enderlein, 1913**
***Gasteruption tonkinense* Pasteels, 1958**
***Gasteruption tournieri* Schletterer, 1885**
***Gasteruption transversiceps* Pasteels, 1958**
***Gasteruption varipes* (Westwood, 1851)**

## Supplementary Material

XML Treatment for Gasteruptiidae

XML Treatment for Gasteruption

XML Treatment for Gasteruption amoyense

XML Treatment for Gasteruption angulatum

XML Treatment for Gasteruption assectator

XML Treatment for Gasteruption assectoides

XML Treatment for Gasteruption bimaculatum

XML Treatment for Gasteruption birmanense

XML Treatment for Gasteruption coloratum

XML Treatment for Gasteruption corniculigerum

XML Treatment for Gasteruption dilutum

XML Treatment for Gasteruption dimidiatum

XML Treatment for Gasteruption formilis

XML Treatment for Gasteruption formosanum

XML Treatment for Gasteruption japonicum

XML Treatment for Gasteruption latitibia

XML Treatment for Gasteruption oriplanum

XML Treatment for Gasteruption parvicollarium

XML Treatment for Gasteruption poecilothecum

XML Treatment for Gasteruption rufescenticorne

XML Treatment for Gasteruption sinarum

XML Treatment for Gasteruption sinepunctatum

XML Treatment for Gasteruption sinicola

XML Treatment for Gasteruption strigosum

XML Treatment for Gasteruption subhamatum

XML Treatment for Gasteruption terebrelligerum

XML Treatment for Gasteruption tonkinense

XML Treatment for Gasteruption tournieri

XML Treatment for Gasteruption transversiceps

XML Treatment for Gasteruption varipes
